# “The best way we can stop suicides is by making lives worth living”: a mixed-methods survey in the UK of perspectives on suicide prevention from the autism community

**DOI:** 10.1016/j.eclinm.2026.103793

**Published:** 2026-03-03

**Authors:** Rachel L. Moseley, Sarah J. Marsden, Mirabel Pelton, Elizabeth Weir, Tanya Procyshyn, Carrie L. Allison, Tracey A. Parsons, Sarah Cassidy, Tanatswa Chikaura, Holly Hodges, David Mosse, Jacqui Rodgers, Ian Hall, Lewis Owens, Jon Cheyette, David Crichton, Darren Hedley, Simon Baron-Cohen

**Affiliations:** a School of Psychology, Bournemouth University, Dorset, UK; b Autism Research Centre, Department of Psychiatry, University of Cambridge, Cambridge, UK; c Department of Haematology, University of Cambridge, Cambridge, UK; d Autism Action of Excellence at Cambridge (now Autism Action), Cambridge, UK; e School of Psychology, University of Nottingham, Nottingham, UK; f Department of Anthropology and Sociology, SOAS University of London, London, UK; g Population Health Sciences Institute, Faculty of Medical Sciences Newcastle University, Newcastle, UK; h East London NHS Foundation Trust, London, UK; i Bournemouth, UK; j Olga Tennison Autism Research Centre, School of Psychology & Public Health, La Trobe University, Victoria, Australia; k Healthy Autistic Life Lab, School of Psychology, Deakin University, Victoria, Australia

**Keywords:** Autism, Suicide, Suicide prevention, Public health, Policy

## Abstract

**Background:**

Autistic people are at higher risk of dying by suicide than are non-autistic people, but research focused on suicide prevention in autistic individuals is lacking. We aimed to understand, from autistic people and those who support them, the pathways to suicide prevention, the balance of crisis measures vs. longer-term prevention, the importance of formal diagnosis, and the role of co-design and co-production in suicide prevention activities.

**Methods:**

We undertook a mixed-methods, two-phase online survey focused on the priorities, views and perspectives of autistic people and their supporters/allies on approaches and strategies to prevent suicide. In Phase 1 (Jan 10–July 31, 2024), via the first survey, we collected and thematically analysed ideas for suicide prevention from almost 1200 autistic people and more than 200 people who identified as supporters and/or allies of autistic people. In Phase 2 (reported herein), a larger group of participants rated and ranked these ideas via a second online survey, during which we collected qualitative and quantitative data. Participants were UK residents aged ≥16 years who self-identified as being in one or more of the following groups: autistic; someone with experience supporting an autistic person of any age or ability; and/or someone with experience of bereavement by the suicide of an autistic person they supported. The online survey included closed-ended and open-ended (qualitative) questions. We thematically analysed free-text responses, and computed descriptive statistics for closed-ended questions.

**Findings:**

Between Nov 1, 2024, and Jan 31, 2025, 2778 individuals responded to the online survey (Phase 2), comprising 2463 autistic people and 315 non-autistic people who identified as supporters/allies of autistic people, some of whom had been bereaved by the suicide of an autistic person. Although some participants reported that crisis interventions (eg, dedicated helplines) were most urgent, a greater number prioritised larger-scale preventative measures (eg, improving support in schools) to address systemic inequalities, which some participants perceived as the root of suicidal thoughts. Across their qualitative and quantitative responses, most participants recommended providing support to people awaiting autism assessment, but some were less supportive of providing dedicated autism supports to people who self-diagnose or who are questioning a possible autistic identity. The qualitative and quantitative data suggested that most participants viewed co-design and co-production of interventions and initiatives to prevent suicide as vital for ensuring these reflect the expertise and empathy that autistic people could bring to individuals struggling with similar experiences.

**Interpretation:**

This qualitative work with experts by experience supports that suicide in autistic people should be viewed in the context of pervasive and systemic injustices, rather than individualistic psychopathology. Accordingly, the extent to which crisis interventions can reduce suicide rates is contingent on additional systemic, preventative, and coordinated actions to tackle the social determinants of suicide and support enduring wellbeing in this group. Support should be produced in partnership with autistic people and their advocates, and be needs-based rather than diagnosis-based. In future research, researchers, practitioners, and policymakers should develop community partnerships to facilitate co-development of strategic, multi-level action plans and initiatives for suicide prevention.

**Funding:**

Autism Action (previously the Autism Centre of Excellence at Cambridge).

**Research in context**

**Evidence before this study**

Autistic people are at significantly increased risk of suicide compared with the general population. Consultation with experts by experience is essential to designing acceptable, appropriate approaches to prevent suicide in specific populations. We aimed to understand whether the perspectives of autistic people had ever been sought with regards suicide prevention. Using search terms “autis∗” AND “suicid∗”, OR “Asperger∗” AND “suicid∗” for relevant work published between database inception and May 2025, we identified relevant titles on PubMed (175 hits), Scopus (191 hits) and PsychINFO (119 hits). We also scrutinised UK policy documents. With very few research articles pertinent to suicide prevention specifically (11.6%), none consulted autistic people about their views on strategies for suicide prevention. A single UK policy brief (2021) sought the views of autistic people on knowledge gaps related to autism and suicide, highlighting the paucity of our understanding around acceptable actions to prevent suicide in this group.

**Added value of this study**

The present study adds essential context to a recent online survey of autism and suicide, the largest to date, which forms Phase 1 of this work and involved over 3000 autistic participants and over 600 people who support, advocate and/or were bereaved by the suicide of an autistic person. Having collected and prioritised ideas for suicide prevention, in this Phase 2 mixed-methods online survey, we sought participants’ views on the direction of suicide prevention efforts across the lifecourse, the provision of support to undiagnosed, possibly autistic individuals, and the importance of community co-production of suicide prevention strategies. Participant responses challenged traditional intrapersonal and pathologising accounts of autistic suicidality––dominant in existing literature on this topic––conceptualising this instead in response to systemic injustices, requiring coordinated, preventative and multisector responses to make lives worth living. Most participants felt strongly that support should be offered to those waiting for autism assessment, since undiagnosed individuals may constitute a large portion of annual suicide deaths. Participants validated the fundamental importance of community partnerships for effective, acceptable and emphatic responses to suicide in this group.

**Implications of all the available evidence**

Our findings from this mixed-methods survey of experts by experience highlight the need for suicide prevention efforts for autistic people to be expansive, preventative, and coordinated. While crisis interventions are desperately needed, they are likely to be insufficient if systemic inequalities continue “setting the seeds” of suicide deaths from childhood onwards. To be effective, it is vital that autistic people and their supporters/allies hold at least equal power in designing and implementing approaches. Herein, community members identified a need for identifying and supporting undiagnosed, yet possibly autistic people, and for needs-based support rather than diagnosis-based support. In future research, researchers, practitioners, and policymakers should develop effective partnerships with autistic people and their supporters/allies in order to co-design strategic plans for suicide prevention initiatives across multiple sectors and settings.

## Introduction

Autistic people live shorter lives in poorer health on average, with suicide a leading cause of death in those without learning disabilities (i.e. intellectual developmental disorder,^1^ or ‘intellectual disability’).^2^ A recent review of child deaths in the UK identified suicide as the most common cause of death in children known to be autistic,^3^ and other reports corroborate the early emergence of suicidal thoughts and behaviour in autistic people and their persistence into midlife and old age.^4^^,^^5^ Environmental and structural contributions to suicidality are prodigious,^6^, ^7^, ^8^ with premature and preventable suicide deaths linked by autistic people themselves to ostracism, victimisation, and social inequities in education, employment, health and social care.^9^, ^10^, ^11^ In the United Kingdom, recognition of autistic people as a priority group for suicide prevention^12^ and review of the Autism Act 2009 (a law to improve the lives of autistic adults) present opportunities for changes that might save lives. Progress in this area is impeded, however, by the dearth of research on actionable practices and policies to reduce the number of autistic people living with the burden of suicidal thoughts and behaviours,^13^ as well as reduce suicide deaths.^14^

In devising suicide prevention strategy, collaboration with priority groups is fundamental to developing acceptable, efficacious and tailored approaches^15^; this may be especially pertinent for autistic people, who are less likely to benefit from standard psychological interventions for mental illnesses associated with suicide.^16^^,^^17^ While autistic people and those who know them best (i.e. those who support and/or advocate for autistic people, henceforth ‘supporters/allies’) are seldom involved in decisions concerning their lives, health and wellbeing, there have been efforts to redress this. A 2021 priority-seeking exercise,^16^ identifying research gaps in the autism and suicide field alongside issues of greatest importance to this community, emphasised the need to collaboratively identify autism-specific pathways to suicide prevention, including “stopgap (i.e. short-term) solutions” and “longer term” approaches. The paucity of strategic actions within the 2023–2028 UK suicide prevention policy for autistic people^12^ underscores this need. Of the present four-point action plan, two points pertain to “develop [ing] a clearer picture” of suicide deaths in autistic people from statutory child mortality records^3^ and the non-statutory, under-resourced and incomplete equivalent for autistic adults and people with learning disabilities.^18^ The two remaining actions request that unspecified parties within the Department of Education and the Department of Health and Social Care “consider” additional support for autistic children and results from an (incorrectly described) investigation of safety-planning,^19^ respectively. With no evidence for the involvement of autistic people and supporters/allies in this proposed strategy, there was little evidence that community priorities^16^ had been heard.

This lack of strategic actions, sans clear evidence of community involvement, motivated our decision to undertake a two-phase survey which, building on the previous research-focused priority-setting exercise, focused on the priorities, views and perspectives of autistic people and their supporters/allies concerning practical approaches and strategies to prevent suicide.^20^ In Phase 1, we collected and thematically analysed 2373 ideas for suicide prevention from almost 1200 autistic people and just over 200 people who identified as supporters and/or allies of autistic people.^20^ We distilled these into 63 re-occurring concrete ideas for pathways to suicide prevention, and in Phase 2, almost 2800 autistic people, and almost 400 non-autistic supporters/allies of autistic people, rated and ranked these ideas. Across the two phases of the survey, and across both autistic and non-autistic participants, there was consistent emphasis on the need to: upskill healthcare professionals; offer autism-specific mental health interventions; expedite diagnosis; provide post-diagnostic support; challenge stigma and discrimination; increase access to financial aid and community-based social care. Though many of our findings echoed the community priorities identified in 2021 –– such as improving healthcare and access to diagnosis –– our participants also prioritised change in sectors which might appear less immediately apparent to suicide prevention, such as education and employment.

While participant selections were indicative of their priorities, Phase 2 participants were relatively constrained by the ideas of Phase 1, and their selections did not clearly address three overarching questions, distinct but interrelated and strongly pertinent to the implementation of suicide prevention strategies for autistic people. The first pertains to whether suicide prevention efforts should focus on crisis interventions, the longer-term solutions, or both.^15^^,^^21^ Indicated interventions, typically situated within mental health services and traditionally prioritised by governments, target individuals already exhibiting suicidal thoughts or behaviours^21^; problematically, their availability to the people who need them is often subject to structural barriers, such as access to a diagnosis and/or to healthcare, which disproportionately affect certain groups within the autistic (and non-autistic) community.^22^ Universal interventions decrease the risk of suicidal thoughts developing in the entire population, through for instance ensuring population health and financial security, but might not help specific groups at higher risk.^21^ Selective interventions might apply similarly broad-stroke measures, but specifically target individuals or groups at risk of *future* suicidal thoughts and behaviour.^21^ While Phase 2 participants had selected both crisis interventions and preventative measures as high priorities, the fact that participants in both phases identified far broader measures pertaining to societal stigma, education and employment as within the scope of ‘suicide prevention’ appeared to corroborate academic consensus that substantive and sustainable reductions in the burden of suicidal thoughts and behaviour require addressing the societal determinants of suicide, such as social injustice and poverty.^15^^,^^21^ Aspiring to better understand participant views from their qualitative data, our first objective was as such to understand the perspectives of autistic people and their supporters/allies towards the question: *Where should suicide prevention efforts be targeted, across the trajectory from autistic individuals who are pre-suicidal to those at crisis point?* (research question [RQ] 1).

The extent that suicide prevention efforts be proactive vs. reactive to present need has ramifications for the potential beneficiaries of these actions. Long-term universal and selective approaches, which aspire to prevent the *development* of suicidal thoughts and behaviour, are likely to be of greatest value to the youngest (and future) generations of autistic children, who are growing up in an era where appropriate recognition and diagnosis as autistic, although by no means straight-forward, is more obtainable than ever before.^23^^,^^24^ Perhaps least served by such approaches are the *many* autistic adults, worldwide, who are undiagnosed,^25^ already at greater risk of lifetime adversity, mental illness, self-harm, suicidal thoughts and behaviour,^26^ and excluded from many types of autism-specific support (such as exists). With highly frequent endorsement of ideas related to diagnosis,^20^ our Phase 1 and Phase 2 samples appeared to make the connection between the issue of suicidal thoughts and behaviour in autistic people and the ‘crisis’^27^ facing overstretched diagnostic services in the UK, and proposed improvements in diagnostic pathways as a means of longer-term suicide prevention via early identification and support.^20^ They recommended proactive screening as a means of identifying adults unaware of their autism,^20^ but otherwise, their responses were inconclusive as to whether and how current services and approaches to support should respond to the ‘lost generation’,^28^^,^^29^ who presently appear to constitute a significant proportion of suicide deaths per annum.^30^ Undiagnosed individuals who are aware they are autistic–including individuals awaiting formal assessment, those wondering if they might be autistic, and those who self-diagnose (a.k.a. self-identify) as autistic^31^—could feasibly be included in interventions targeted at autistic people in crisis. To our knowledge, however, the autism community have never been consulted regards the appropriateness of this approach. Our second objective was therefore to address a question thus far unanswered in autism research: *What were the views of autistic people and their supporters/allies towards making dedicated autism* supports *and services available to undiagnosed people who think they might be autistic?* (RQ2).

In so much as we desired to learn views of the community regarding these two issues, we also sought to understand their views regarding the importance and added value of community involvement itself. While co-production of suicide prevention efforts was emphasised in the aforementioned 2021 priority-setting exercise,^16^ participants did not expound on the underlying reasons for this need, and co-production has been rarely implemented in practice. Partnership with diverse community members, including those who support autistic children and those not (yet) formally diagnosed as autistic, may present itself as a means of expanding perspectives on suicide prevention for autistic people, ensuring that preventative, universal approaches are considered to disrupt the trajectory to suicidal thoughts, alongside needs-based crisis support not contingent on diagnosis. With this in mind, to better understand and advocate for the importance of community partnerships, our third objective was therefore to explore the question: *What were the attitudes of participants towards autistic involvement in co-designing and co-producing actions for suicide prevention*? (RQ3) In addressing these three questions, our study aimed to provide the necessary contextualising information to translate the community's ideas into reality.^20^

## Methods

### Study design and ethics

**Fig. 1:**
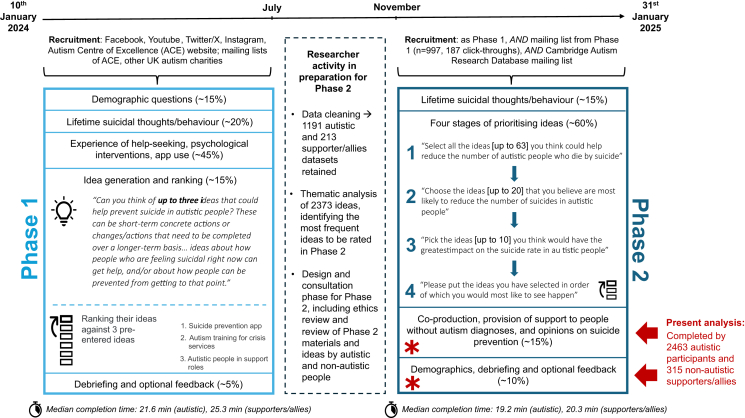
*Situation of the present data within the two-phase study*. *Note*. Fig. 1 depicts the time course of the two-phase study; researcher activity, including recruitment, analysis of Phase 1 data, and design of Phase 2; and participant activity in the Phase 1 and Phase 2 surveys. Red asterisks and text mark the sections which are the focus of this paper; other aspects of the data are reported elsewhere.

We conducted a two-phase suicide prevention study involving two online surveys (see Fig. 1). The first was active between Jan 10, 2024 and July 31, 2024 (Phase 1), and the second, from which the data herein originate, between Nov 1, 2024 and Jan 31, 2025 (Phase 2).

The two-phase study was approved by the Cambridge University Psychology Research Ethics Committee (PRE.2022.097; PRE.2024.048) and sponsored by the same institution. Participants all provided electronic consent to participate and for their data to be included anonymously in publications.

### Participants

Participants (*n =* 2778) were included in the present study if they had completed the relevant section of the second phase of our survey on suicide prevention^20^ (see Fig. 1; Supplementary Materials 1 provides additional information on quality control checks and inclusion criteria for participant datasets), which involved both closed-ended and open-ended (qualitative) questions. Participants were UK residents who were aged at least 16 years and belonged to one or more of the following groups a) autistic b) someone with experience supporting an autistic person of any age or ability, and/or c) someone with experience of bereavement by the suicide of an autistic person they supported. We differentiate in this paper between non-autistic people with experience of supporting and/or bereavement (henceforth “non-autistic supporters/allies”, n = 315) and autistic participants (n = 2463, some of whom also had experience of supporting and/or bereavement). Participants were required to be residing in the UK, given many ideas referred to UK services.

### Procedures

The Phase 2 data analysed herein were fundamentally shaped by Phase 1, where, among other information,^9^^,^^10^ we collected ideas for suicide prevention (i.e. suggested courses of action) from autistic people and their supporters/allies (Fig. 1, Supplementary Materials, 3). In the Phase 2 survey, the participants of this paper selected which of these ideas were their priorities for suicide prevention.^20^ The survey items analysed herein were immediately after the ranking exercise in the following order:

#### A closed-ended question on the importance of co-design and co-production

In a question specifically pertaining to RQ3, we asked participants to indicate the importance of autistic people being “involved in the design and running of support, services and products for autistic people”, explaining this was also known as co-production and co-design. Response options were ‘very important’, ‘moderately important’, ‘slightly important’, ‘not important at all’, or ‘unsure or prefer not to say’.

#### Six closed-ended questions concerning provision of autism-specific support to people who think they might be autistic

In relation to RQ2, we asked participants whether their prioritised ideas for suicide prevention should be available to ‘people awaiting an autism assessment’, ‘people who self-diagnose as autistic’, ‘people who think they might be autistic’ or ‘all of these groups’. In five following questions, we posed the same question regarding support in education, employment, social care, health care (specifically the ‘National Health Service’ [NHS], the publicly-funded health system of the UK), and criminal justice sectors.

#### Two opportunities for free-text responses

Participants were subsequently offered a free-text opportunity to explain their choices in the ranking exercise they had just completed. Another free-text opportunity followed, after some brief demographic questions, and offered participants the chance to share any remaining thoughts or opinions. Responses to each question were limited to 200 characters, could be relevant to any of the three RQ, and were combined in thematic analysis.

### Qualitative analysis

Our analyses pertained to 1) the staging or direction of suicide prevention efforts towards crisis intervention vs. prevention (RQ1), 2) the provision of autism-specific support to people awaiting autism diagnosis, those who self-diagnose and those who believe they may be autistic (RQ2), and 3) the involvement of autistic people in co-designing and co-producing suicide prevention efforts (RQ3). Findings in relation to these topics were chiefly informed by qualitative analyses in an inductive thematic approach albeit with pre-defined questions.^32^ Of 859 responses to the first qualitative question, 773 (90%) were from autistic participants. Most (73.7%) responses constituted attitudes about suicide prevention, ranging from reiteration of the importance of certain ideas; reference to participants' own suicidal experiences (or those of the person they supported/advocated for); and explicit reflections on the staging of suicide prevention efforts as pertaining to RQ1. The remaining responses included or pertained to provision of autism-specific support to people who may be autistic (26.9%, RQ2), and/or to co-design and co-production (13.3%, RQ3). We excluded from qualitative analyses responses to the first question which did not clearly pertain to any of these topics, were difficult to interpret, and/or very specifically described how individual participants completed or experienced the ranking exercise (for instance, “I found the ranking difficult because the sentences didn't stay in my head long enough”)—altogether 8.1% of responses.

Of 700 responses to the second qualitative item, 604 (86.3%) were from autistic participants: the majority (62%) pertained to attitudes about suicide prevention and/or personal experiences (RQ1), while a small proportion included or solely reflected attitudes about provision of autism-specific supports to people who may be autistic (1.6%, RQ2) and/or co-design and co-production (2.1%, RQ3). We excluded from analysis responses to the second question which were remarks on the design and/or implementation of the survey (eg “Thank you for asking us what we need”)—these constituted 42.7% of responses to this question.

Having initially coded the perceived relevance of responses to the three topics above, the first author identified more fine-grained codes in each category of response. Following iterative review and refinement, they interpreted higher-order themes and subthemes related to these three topics, which are presented sequentially in the Results section. Please see Supplementary Materials (4) for steps taken the ensure trustworthiness of the data, and Supplementary Materials (5) for full thematic tables, in which are included the percentages of autistic (formally diagnosed and possibly autistic) and non-autistic participants who contributed to each theme and subtheme. All analyses were planned (i.e. pre-specified).

### Statistical analysis

For additional insights related specifically to provision of autism-specific support to people not formally diagnosed as autistic (RQ2), and to co-design and co-production (RQ3), we computed descriptive statistics from the closed-ended items with pre-entered answers. From the six questions assessing provision of autism-specific support in different sectors, we calculated the percentage of autistic participants and supporters/allies who endorsed availability of support to people awaiting autism assessment, those who self-diagnose, and those who thought they might be autistic (combining instances where participants selected each group specifically and where they selected ‘all of these groups’). Using the question about co-design and co-production, we calculated the percentage of autistic people and supporters/allies who endorsed these as ‘very’ to ‘not important at all’. These descriptive analyses are displayed alongside qualitative findings pertaining to provision of autism-specific support to people not formally diagnosed as autistic and to those pertaining to co-design and co-production. As the principal focus of analyses was qualitative, we performed no additional statistical analyses, sensitivity analyses or post-hoc analyses related to our research questions.

### Community involvement

While the research team incorporates autistic individuals, those with lived experience of suicidal thoughts and/or behaviour, of supporting someone through suicidal thoughts/behaviour, and/or bereavement by suicide, we endeavoured to embed community participation throughout the two-phase study. We sought the views of autistic people and their supporters/allies as pertains to the design and analyses of Phase 1 and Phase 2, and invited community members from both groups to read the manuscript for acceptability. Community involvement is detailed in Supplementary Materials (6).

### Role of the funding source

Online promotion of both survey phases was funded by the charity Autism Action (previously the Autism Centre of Excellence at Cambridge [ACE]). Authors TAP and JC (current and past employees of Autism Action) and SBC, IH and LO (trustees of Autism Action) contributed to the design of the study, but not data analysis (while not being precluded from the data); they reviewed the written manuscript, agreed to and accept responsibility for publication.

## Results

### Study population

Between Nov 1, 2024, and Jan 31, 2025, 2778 participants responded to the online survey (Phase 2) and met quality control criteria so as to be included in present analyses (see Supplementary Materials, 1). These 2778 respondents included 2463 autistic people and 315 non-autistic supporters/allies. Demographic data from these autistic and non-autistic participants are presented in Table 1. Not all participants who completed this section of the survey took up the opportunity to provide qualitative data in response to open-ended questions; Table 1 therefore also reflects information about those participants who did. Additional information is given in Supplementary Materials (2).

**Table 1:** Participant demographic information.

	Autistic participants		Non-autistic supporters/allies	
	Whole sample (n = 2463)	Qualitative sample (n = 989)	Whole sample (n = 315)	Qualitative sample (n = 130)
**Average age** (SD, *range*)	40.2 (14.4, 16–89)	42.7 (14.1, 16–89)	52.5 (11.7, 16–89)	54.2 (10.9, 20–86)
% Missing data	0.3%	0.1%	0.6%	0.8%
**Gender**				
% Cisgender men	15%	16%	8.6%	6.9%
% Cisgender woman	64.4%	62.9%	87.3%	90.0%
% Trans, gender-divergent or gender-questioning	19.7%	20.0%	2.9%	1.5%
*Within this group*				
% Transgender men	14%	12.6%	33.3%	0%
% Transgender women	3.9%	5.1%	11.1%	0%
% Non-binary identities (including agender, genderfluid)	57.6%	56%	22.2%	50%
% Unsure right now	24.5%	26.3%	33.3%	50%
% Missing data	0.9%	1.1%	1.3%	1.5%
**Sex**				
% Male	17.9%	18.9%	8.9%	6.9%
% Female	80.8%	79.8%	88.3%	91.5%
% Intersex or other	0.6%	0.6%	0.6%	0.6%
% Missing	0.7%	0.7%	2.2%	0.9%
**Ethnicity**				
% White	89.8%	88.5%	94.0%	94.6%
% Person of colour [POC]/Missing	9.7%/0.5%	11.2%/0.4%	4.4%/1.6%	3.8%/1.6%
**Highest educational attainment**				
% GCSE/high school diploma/equivalent or lower	8.9%	7.2%	8.9%	6.2%
% AS/A Levels/Access to HE/equivalent	11.1%	10.6%	11.7%	12.3%
% Diploma, degree or higher	66.3%	69.3%	65.1%	68.5%
% Rather not say/Missing	13.5%/0.3%	12.6%/0.3%	13.7%/0.6%	12.3%/0.8%
**Employment/occupation**				
% Any kind of employment	55.4%	54.2%	64.4%	65.4%
% Studying	13.4%	10.2%	2.2%	0.8%
% Rather not say/Missing	0.9%/0.1%	0.8%/0.1%	2.2%/0%	2.3%/0%
**Self-reported autistic status**				
% Formally diagnosed	66.6%	68.7%		
Average age at diagnosis (SD, range)	34.2 (15.1, 2–74)	36.3 (15, 2–70)		
% Did not provide age at diagnosis	34.8%	32.5%		
% Possibly autistic (awaiting assessment or self-diagnosed)	32.4%	31.2%		
% Missing data	1%	0.1%		
**Experience of suicidal thoughts/behaviour**				
% Never suicidal	2.0%	1.6%		
% Passing thoughts of suicide	9.2%	10.6%		
% Suicide ideation	19.3%	17.7%		
% Suicide plans	26.6%	28.1%		
% Attempted suicide at least once	42.9%	42.0%		
**Recency of suicidal ideation/attempts if relevant**				
% Within past 12 months	73.2%/19.8%	70.5%/17.3%		
% Within past 5 years	13.4%/31.9%	14.8%/28%		
% Over 5 years ago	11.4%/48.2%	13.1%/54.7%		
% Missing	2%/0.01%	1.6%/0%		
**% Without “supporter” status (experience of supporting an autistic person and/or bereavement by the suicide of autistic person)**	75.7%	74.3%	0%	0%
**% With “supporter” status**	24.3%	25.7%	100%	100%
**Contextualising information for participants with supporter/ally status**				
***% Supporting/advocating for an autistic person without known experience of suicidality***	25.9%	22%	13.3%	10.8%
% As their parent/guardian	62.5%	64.3%	76.2%	71.4%
% In another familial relationship	18.7%	14.3%	11.9%	7.1%
% As a friend or partner	16.8%	21.4%	4.8%	7.1%
% In another kind of relationship	1.9%	0%	7.1%	14.3%
*Average age of autistic person (SD, range)*	23.8 (18.6, 1–64)	22.7 (15.4, 5–88)	18.2 (8.2, 6–38)	19.7 (7.8, 9–35)
*Gender identity of autistic person*				
% Cisgender male/% Cisgender female	51.6%/31%	57.1%/28.6%	59.9%/26.2%	64.3%/21.4%
% Transgender or gender-divergent	3.9%	12.5%	7.1%	0%
% Not sure how they identify/% Missing	6.5/0%	1.8%/0%	7.1%/0%	14.3%/0%
*Diagnostic status of the autistic person*				
% Formally diagnosed	70.3%	67.9%	95.2%	100%
Average age at diagnosis (SD, range)	11.5 (10.9, 1–64)	13.1 (13.2, 2–58)	8.8 (4.8, 2–22)	8.5 (6.1, 2–22)
% Believed by participant to be autistic	7.7%	5.4%	2.4%	0%
% On the waiting list for assessment	10.2%	10.8%	0%	0%
% Self-identifies as autistic and/or in the process of seeking assessment (not yet on waiting list)	11.8%/0	15.9%	2.4%	0%
***% Supporting/advocating for an autistic person known or suspected to have experienced suicidal thoughts/behaviour***	64.1%	65.3%	74.6%	71.5%
% As their parent/guardian	58.3%	54.2%	100%	100%
% In another familial relationship	13.8%	16.9%	0%	0%
% As a friend or partner	22.7%	24.1%	0%	0%
% In another kind of relationship	5.2%	4.8%	0%	0%
*Average age of autistic person (SD, range)*	24.9 (13, 6–85)	26.1 (13.8, 6–85)	24.5 (11.8, 8–72)	25.9 (12.9, 8–68)
*Gender identity of autistic person*				
% Cisgender male/% Cisgender female	44.8%/32.6%	48.2%/29.5%	44%/37.1%	46.5%/37.4%
% Transgender or gender-divergent	14.8%	12%	12.9%	11.1%
% Not sure how they identify/% Missing	2.6%/5.2%	5.4%/4.8%	3.2%/2.8%	4%/1%
*Diagnostic status of the autistic person*				
% Formally diagnosed	78.1%	77.1%	83.9%	87.9%
Average age at diagnosis (SD, range)	16 (11.2, 2–64)	17 (13.2, 2–64)	15.3 (8.7, 2–50)	15.7 (9.1, 4–50)
% Believed by participant to be autistic	2.9%	3%	2.8%	4%
% On the waiting list for assessment	7.9%	6.6%	6.4%	5%
% Self-identifies as autistic and/or in the process of seeking assessment (not yet on waiting list)	11.1%	13.3%	6.9%	3.1%
**% Bereaved by the suicide of an autistic person**	9.7%	11.8%	9.5%	13.9%
% As their parent/guardian	17.2%	16.7%	46.7%	55.6%
% In another familial relationship	24.1%	3%	16.7%	16.7%
% As a friend or partner	51.7%	46.7%	33.3%	27.8%
% In another kind of relationship	6.9%	6.7%	3.3%	0%
*Average age of autistic person when they died (SD, range)*	32.3 (16.4, 7–73)	36.2 (17.1, 7–73)	32.1 (14.7, 11–63)	31.1 (15.8, 11–63)
*Gender identity of autistic person*				
% Cisgender male/% Cisgender female	56.9%/24.1%	66.7%/20%	60%/30%	50%/33.3%
% Transgender or gender-divergent	10.3%	10%	6.6%	11.2%
% Not sure how they identified/% Missing	3.4%/5.2%	3.3%/0%	0%/0%	0%/5.6%
*Diagnostic status of the individual who died*				
% Formally diagnosed	31%	16.7%	50%	61.1%
Average age at diagnosis (SD, range)	16 (11.1, 1–50)	22.5 (19.1, 6–50)	20.4 (16.7, 6–63)	20.3 (16.7, 6–63)
% Believed by participant to be autistic	43.1%	53.3%	23.3%	22.2%
% On the waiting list for assessment when they died	3.4%	3.3%	9.9%	16.8%
% Self-identified as autistic and/or was in the process of seeking assessment when they died (not yet on waiting list)	22.5%	26.7%	16.8%	0%

Note. Demographic information of autistic participants and non-autistic supporters/allies. For descriptive purposes, we utilised questions on sex assigned at birth and gender identity to categorise participants as per the groups in the table; in the qualitative data, participant quotations are accompanied by their own preferred terms. Notably, with a single question assessing identification with different ethnic groups, the survey did not enquire about race and ethnicity as distinct constructs. More detail of our assessment of sex and gender, ethnicity, and self-reported autistic status is provided in Supplementary Materials 2, along with sample details related to these variables.

### RQ1: attitudes about the staging of suicide prevention

We interpreted two higher-order themes in the qualitative data related to this topic (Table 2; Supplementary Materials, 5). The first reflected explicit consideration of balancing indicated interventions with selective or even universal approaches. The second, reflecting need for systemic change, implicitly speaks to the necessity of expansive, preventative approaches.

**Table 2:** Themes and subthemes related to the staging of suicide prevention efforts.

Themes, *subthemes*	Quotations from autistic participants and non-autistic supporters/allies
**1. The “urgent” vs. the “root causes”** This theme reflected participants' deliberations on the staging of suicide prevention efforts between indicated interventions, helping people already experiencing suicidal thoughts and/or behaviour, and selective and/or universal approaches focused on preventing the development of suicidal thoughts and/or behaviour. Some participants explicitly reflected on the difficulty of the choice between approaches.	“Some proposals addressed root causes of mental ill health, whereas others were more ‘urgent’ (e.g. crisis support). I struggled to choose between most effective and most urgent” (autistic cisgender woman, 35, undiagnosed, England) “Difficult to prioritise between measures which are of broad benefit & will mean fewer people go far up Stress and Desperation Mountain, and those which … stop people jumping off!” (autistic cisgender man, 62, undiagnosed, England) “I tended to prioritise crisis services, while recognising that prevention (in various ways and at different life-stages) is at least as important” (autistic cisgender woman, 70, diagnosed, Wales) “I prioritised early intervention over crisis care because I was going for ideals. However crisis care may be more important primarily until better systems are established” (autistic cisgender woman, 31, undiagnosed, England) “Some of these are really important for supporting autistic people who are actively suicidal, but it's also important to achieve wider societal change to prevent people reaching that point” (autistic non-binary individual, 22, diagnosed, England) “Short term the lost generation are still being let down by long waiting lists, lack of pre and post dx [diagnostic] support. Long term, education and awareness and understanding across the population is key” (autistic gender-questioning individual, 42, diagnosed, England) “Lack of support in childhood set me up for problems for rest of my life. Support with education and career is vital. Those of us that is too late for understanding and safe place are vital” (autistic cisgender woman, 43, diagnosed, England) “I am part of the lost generation of autistic women and I suspect that what we need may be different from younger women who have not had a lifetime of trauma and masking. Different solutions needed” (autistic cisgender woman, 61, undiagnosed, England)
***i. “The seed of all autistic suicide death is in childhood and early adulthood”*** This subtheme reflected comments from participants who prioritised longer-term, preventative (universal or selective) approaches to suicide prevention over those for people in crisis.	“Although I believe the peak age for suicide is the 30s, the greatest waste of life and the seed of all autistic suicide death, is in childhood and early adulthood. Hence my emphasising schools & unis” (autistic cisgender man, 54, diagnosed, England) “I feel school is where it all starts. there needs to be better support for autistic people in schools, all staff should have mandatory training and support needs to be so much better than it is now” (autistic cisgender woman, 38, diagnosed, England) “I have worked in the NHS for over 25 years. My work has shown that early intervention is overlooked and crisis management is prioritised. It starts in childhood, we must start from the beginning” (autistic non-binary individual, 51, diagnosed, England) “It begins with bullying in schools. Interestingly it takes many years for a ‘professional’ to identify that you're autistic, but a child of 7 can spot you and start making your life hell from then on” (autistic cisgender woman, 60, diagnosed, England) “Bullying in school first as if children hadn't bullied and shunned him and understood autism my son would not have felt suicidal in the first place. He was a happy boy before he went to school” (non-autistic supporter/ally, cisgender woman, 53, England) “I am a broken person & acutely suffering because of not having special needs support, or intervention, especially in early childhood, teens or adulthood” (autistic cisgender woman, 55, diagnosed, England) “Children and teenagers need to understand themselves and their brains. I spent my childhood feeling alone, like a freak. I wasted my life. We need support earlier for young people” (autistic cisgender woman, 41, diagnosed, England) “Reduce suicide by reducing trauma. That starts in society b4 MH [mental health] services needed” (autistic cisgender woman, 49, diagnosed, England) “Address the root problem: Education, bullying/school life, diagnosis–reduce the number of autistics traumatised before they reach adulthood, and ill-equipped for independent life. Financial help” (autistic gender-questioning individual, 31, diagnosed, England) “Mental health support will fall short if an autistic's financial, material or physical needs remain unmet. We must ensure autistic's have a foundation of safety, addressing the causes of despair” (autistic cisgender woman, 25, undiagnosed, England) “The priority should be to helping them in their normal life. These day-to-day struggles are what accumulates to the major feeling of loss of hope & desire to continue living. And parents need this too” (non-autistic supporter/ally, cisgender woman, 20, England) “All these ideas were good. It was very difficult to choose what to prioritise. In an ideal world I would rather prioritise improving everyone's quality of life to help prevent mental illness” (autistic cisgender woman, 34, diagnosed, England) “I feel like the route to stopping people getting to crisis is helping them understand themselves and their reactions/emotions better earlier in life so the bad stuff doesn't add up” (autistic cisgender woman, 50, undiagnosed, England) “Autistic children need skills to thrive that take them to adulthood, especially Asperger children. They are clever but not socially which leads to social isolation and suicide” (autistic cisgender woman, 51, diagnostic status not provided, England) “The best way we can stop suicides is by making lives worth living–friends, lovers, community, financial security via adapting the world of work to accommodate autism” (autistic non-binary individual, 29, undiagnosed, England)
***ii. “Crisis* 1st*, preventing crisis 2nd, quality meaningful satisfying life 3rd”*** This subtheme reflected comments from participants who prioritised indicated crisis measures foremost, relegating longer-term preventative approaches to a later point in time.	“Crisis 1st. Preventing crisis 2nd. Quality Meaningful Satisfying Life 3rd” (autistic transgender woman, 68, undiagnosed, England) “1. Immediate help for those most at risk. 2. Help that is designed for autistics (with possible alexithymia). 3. GP Support for all autistics *[author note: GPs, General Practitioners, are primary care doctors who are typically the first point of patient contact for all services within the NHS]*. 4. Better life experience for upcoming generations” (autistic cisgender woman, 45, undiagnosed, England) “1 Helping person in crisis. 2 foundations to prevent crisis (breaks from education, help w benefits). Less important: clubs, self-advocacy classes, help in small way but don't address core issues” (autistic non-binary individual, 24, diagnosed, England) “As someone who has made attempts many times–the vital component is quicker access to MH [mental health] care. 6–24 month wait lists are insane and causing deaths. Crisis care is also very poor/barely existent” (autistic cisgender woman, 34, diagnosed, England) “I chose the autism-friendly safe space and helpline during suicidal crisis as particularly important because being able to stay at the Edinburgh Crisis Centre has saved my life on several occasions” (autistic transgender man, 44, undiagnosed, England) “When you are suicidal, quick help is needed, hence being able to contact someone without speaking or battling a GP receptionist, who does not think your case is urgent enough if vital” (autistic cisgender woman, 45, diagnosed, England)
**2. “Brutalised, broken systems”—the logic of suicide and the need for “radical social change”** This theme incorporated comments where participants remarked on the scale and breadth of efforts needed to address the problem of suicide with and for autistic people. In some quotations, participants expressed a sense that suicide was a logical or rational response to systemic inequalities.	“There are so many gaps in understanding and provision that even the ten I picked are the tip of a very large iceberg of need” (autistic cisgender woman, 46, diagnosed, England) “Until we change the system, which wasn't built for us, we're stuck playing whack-a-mole” (autistic cisgender man, 23, diagnosed, England) “The world needs to get a lot better about not crushing the souls out of autistic folk” (autistic non-binary individual, 36, undiagnosed, England) “School and the NHS have done irreparable harm to me and they can never be trusted again. I hope that these brutalised, broken systems are improved but I do not foresee this happening in my lifetime” (autistic gendervoid individual, 34, diagnosed, Scotland) “The current system isn't fit for purpose. My son went into crisis and was high risk of suicide we had no help. I had to get him through it myself” (non-autistic supporter/ally, cisgender woman, 40, England) “I lost my sister, and the cracks in the systems were very noticeable” (non-autistic bereaved supporter/ally, cisgender woman, 23, Scotland) “Adults with autism who need only support with day to day tasks are not supported in any way only by family” (non-autistic supporter/ally, cisgender woman, 57, England) “This is so relevant for my daughter at the moment. She is quite often suicidal and her life is so limited that I totally understand why. She is 17 and has no friends. She struggles to go out” (non-autistic supporter/ally, cisgender woman, 57, England) “My suicide attempt wasn't a mental health crisis. It was just I knew I couldn't be who society wanted me to be. I suspect there's a lot of suicides like my attempt, where no crisis is involved” (autistic cisgender woman, 50, undiagnosed, Wales) “I now fear for the future and how I would cope if my partner died … There is no other support available so I have an exit plan” (autistic cisgender woman, 65, diagnosed, Wales) “I work in schools … I see it every day. I hear about early intervention every day … but it doesn't happen. My kids were failed. I was failed. Suicide in autistic people is logical. Living is hard” (autistic cisgender woman, 42, undiagnosed, Scotland) “Suicidal ideation/intent must be recognised as a rational response to an existence of suffering &struggle” (autistic cisgender woman, 45, diagnosed, England) “Every time the government says we're lying I get a bit closer to the edge. This world isn't for us. We can't be different, but they don't believe us, what's the point?” (autistic cisgender woman, 62, diagnosed, England) “Why protect us from suicide when we are made actively vulnerable by NHS Policy and Government politics?” (autistic cisgender woman, 53, diagnosed, Scotland) “Suicide ‘prevention’ must go hand in hand with respecting autonomy and validating chronic suicidal ideation without resorting to carceral measures” (autistic non-binary individual, 28, diagnosed, England)
***i. Acceptance, belonging and community*** This subtheme reflected unmet needs for social contact, reciprocal accepting relationships and (in several participants' words) ‘community’. Some quotations within this subtheme reflected on the contributions of alienation, loneliness, and unbelonging, alongside instances of explicit rejection and exclusion, to suicidal thoughts and behaviours. Others explicitly pointed to the importance of measures combatting stigma and social ostracism of autistic people as a priority for suicide prevention.	“I believe isolation and loneliness to be the greatest hardships suffered by my son who has autism” (non-autistic supporter/ally, cisgender woman, 73, England) “My choice was primarily based on my personal experience. Finding a peer group was the most profound support for my suicidal ideation and losing that peer Group was the most profound knock back” (autistic cisgender man, 42, diagnosed, England) “To reduce suicide ideation, autistics need to feel much less alienated. We need help to allow us to function more fully as part of society. Isolation is the hardest thing …” (autistic cisgender woman, 72, diagnosed, England)
***ii. Accessible, appropriate and trustworthy healthcare and crisis services*** This subtheme reflects comments pertaining to the UK's National Health Service (NHS) and to crisis services. Participants frequently commented on insufficient understanding of autism in healthcare professionals, some positioning the NHS as ‘actively harmful’ for autistic people. Others spoke of barriers to healthcare access (e.g. phoning for a GP appointment); lack of adapted services and approaches for autistic people; and of experiences of being rejected due to being autistic and/or too ‘complex’. Some quotes also mentioned insufficiency of state and charity sector crisis services. Some directly called for specialist services for neurodivergent people, or at the least, the need for existing services to ‘gear up’ to work appropriately with and be accessible for autistic people.	“It is appalling to me that I can be sectioned for being autistic under the MHact [Mental Health Act], but the clinicians responsible have no idea how to help in a crisis. They make it worse, make you feel no one can help” (autistic cisgender woman, 33, diagnosed, Wales) “A phone line is only helpful if it actually helps, endless signposting contributes to suicide. Current ‘help’ is fractured, unco-ordinated smoke and mirrors, none of it is autism or carer friendly” (autistic cisgender woman, 50, undiagnosed, England) “At the moment, the NHS is actively harmful to autistic people looking for mental health support. Early intervention and genuine support needs to happen” (autistic cisgender woman, 54, diagnosed, England)
***iii. Access to diagnosis and post-diagnostic care*** This subtheme reflected numerous comments where participants reflected on the detrimental impacts of being undiagnosed, and/or the contributions of being missed for diagnosis to their suicidal experiences; or, conversely, the ameliorative or beneficial effects of finally being diagnosed or realising an autistic identity. Some also reflected on the inadequacy of post-diagnostic support and emphasised how sorely they had needed it to recover from suicidal thoughts and/or behaviour. The subtheme also included direct calls to improve and increase the capacity of diagnostic pathways, focus on early identification, and identify undiagnosed, missed adults.	“I was diagnosed at 44 but have had no follow up support. I think if you're ‘good’ at managing your own mental health and suicidal ideation then the NHS lets you get on with it on your own” (autistic cisgender woman, 48, diagnosed, England) “I never had any support or help to understand my Autism. And this led inpart to suicide attempts. Finding information has been near impossible and I have learnt to cope or not most of the time” (autistic cisgender woman, 65, diagnosed, England) “Every turn is difficult when trying to get a diagnosis and support. As a parent, you have to fight for everything and you shouldn't have to. I was told to keep my child away from anything sharp” (non-autistic supporter/ally, cisgender woman, 54, England)
***iv. Suitable educational provision and transition support*** This subtheme reflected comments from participants who attributed their suicidal experiences, at least in part, to a lack of support and accessibility within the education system. Some noted that with children unable to attend school, punitive measures taken towards parents and families by the education system resulted in a desperate, helpless situation. Others particularly highlighted the insufficiency of support at transition points, such as the end of formal education. This subtheme also included quotations calling for reform of the education system, which participants suggested needed to become fundamentally more flexible and individual-centred, responsive to and respectful of neurodivergent needs.	“Me and so many autistic people I know feel absolutely useless and without hope at this age (I'm 21, my friends are 17–22) because we feel burnt out and lost after leaving school/college” (autistic transgender man, 21, undiagnosed, England) “Autistic children's families are being threatened with fines and prison because they've made mainstream school inaccessible for autistic children” (autistic cisgender woman, 44, diagnosed, England) “We need an education system that is flexible and can meet the needs of autistic kids without requiring them to exhibit severe signs of trauma before providing accommodations and alternative routes” (autistic cisgender woman, 42, diagnosed, England)
***v. Suitable employment and social care in the community*** This subtheme reflected comments from participants highlighting the inadequacy of current social care provision and/or community-based support ‘beyond’ the healthcare system. Some participants highlighted their need for regular support with daily life tasks and participation in the community. Some spoke about challenges obtaining and maintaining employment, with some speaking of inaccessibility and bullying in the workplace. Some participants explicitly highlighted need for better resourcing and training in the social care sector such that would translate to ‘helping them [autistic people] in their normal life’. Others directly called for employment-focused support, increasing awareness among employers, and for law to make workplaces fairer and accessible.	“I think the most important thing is to improve the ability for autistic people to participate in society and use their skills to benefit their community while still allowing for rest” (autistic cisgender woman, 25, diagnosed, England) “I feel it is important to ensure neurodivergent individuals are recruited into different job roles aligned to capability and clear process have to be followed support positive work placements” (autistic cisgender woman, 62, diagnosed, England) “I ranked it based on what I would need to live a healthy and fulfilling life. I'm waiting on a social worker and I barely make it through day to day” (autistic transmasculine genderqueer individual, 24, diagnosed, Scotland)
***vi. Financial security*** This subtheme reflected comments from participants pertaining to poverty and/or financial insecurity as a contributing factor to suicidal thoughts and/or behaviour. Some participants highlighted that financial insecurity forced them into untenable or unbearable employment and/or residential circumstances. Some participants highlighted the inaccessibility of the processes necessary to claim disability benefits, the insufficient knowledge and/or negative attitudes of benefits assessors. This subtheme also contained direct calls for reform of the disability benefits assessment process and training for its staff, measures to tackle poverty and increase financial aid for autistic people.	“I ranked financial support #1 because too many are stuck in a cycle of recurring autistic burnout (often with suicidality) because of pushing themselves past their limits to make enough money to live” (autistic cisgender woman, 38, diagnosed, England) “Many kill themselves after losing their support network and financial support. They struggle to work or pay rent. The fear is overwhelming. Most live with others and don't work. They need money” (autistic cisgender woman, 28, diagnosed, England) “The DWP *[author note: Department of Work and Pensions, the UK Government department responsible for policies concerning financial support—‘benefits’–for those with disabilities or health conditions]* and the benefits system has been one of the biggest causes of my mental decline, as well as the current demonisation of mental health and benefits” (autistic cisgender woman, 40, diagnosed, England)
***vii. Safety from victimisation and equity within the criminal justice system (CJS)*** This subtheme reflected comments from participants reflecting pervasive victimisation (‘bullying’) -during school, in the workplace, from society generally, and/or perpetrated by authorities (such as health- and social care professionals)–as a contributing factor to suicidal thoughts and behaviour. Others reflected on harmful encounters with and persecution by the criminal justice system, and/or highlighted insufficient autism awareness in police. Some participants explicitly called for stricter legal sanctions to protect autistic people from victimisation and penalise those who perpetrate it. Others called for improved training and processes within the criminal justice system, such that would benefit autistic people who have contact with the police and those who are incarcerated.	“My son has a 2:1 Law degree and is experiencing huge and extensive disability discrimination in the workplace which has lead to his second attempt at suicide” (non-autistic supporter/ally, cisgender woman, 57, England) “The CJS needs serious work as well. I was accused of being a gifted hacker & stalker … [redacted locality] police said because i can talk I'm not a vulnerable person. Apparently it was reasonable” (autistic cisgender man, 37, diagnosed, England) “Bullying wrecks lives and autistic people are a target for this. Autistic people can only reach their full potential if bullying is stamped out at school and work” (autistic cisgender woman, 50, diagnosed, England)
***viii. Equality for diverse identities and needs*** This subtheme reflected comments pertaining to differing intersectional needs that are insufficiently catered for in services and support. The frequency of missed and misdiagnoses for girls, women and people assigned female at birth, and poorer understanding and support for the same, was the most commonly mentioned area of need. However, some participants also highlighted the unmet needs of autistic people of colour, those with co-occurring ADHD and/or varying profiles of support needs, those who are trans or with other non-binary and/or non-heterosexual identities, and those from poorer backgrounds. This subtheme also included comments calling for changes to specifically help these groups, or draw attention to their needs.	“For suicide prevention we need radical social change with a redistribution of resources and disability, racial, queer, climate and all other forms of justice” (autistic gender-questioning individual, 24, undiagnosed, England) “Access to diagnosis more available and awareness to marginalised groups (POC, immigrants, high functioning [sic], women)” (autistic cisgender woman, 18, diagnosed, England) “So many people go undiagnosed for too long–especially females and ethnic minorities. Going through life undiagnosed is mentally, emotionally, physically exhausting and traumatising = suicidal” (autistic cisgender woman, 45, undiagnosed, England)

Note. Quotations from autistic participants and non-autistic supporters/allies as pertaining to the staging of suicide prevention efforts. Quotations from autistic participants are inclusive of formally diagnosed and possibly autistic individuals. Acronyms used by participants are explained by italicised author notes.

#### Theme 1: the “urgent” vs. the “root causes”

This theme reflected comments where participants deliberated between prioritisation of indicated vs. selective or universal approaches. Many participants stated the difficulty of choosing between crisis and preventative measures, recognising as the former as important to “the lost generation” who require immediate support, and the latter to the younger generation not yet “far up Stress and Desperation mountain”. While 14.8% of these comments reflected more balanced prioritisation of these approaches, two subthemes represented seemingly more confident preferences:

**“The seed of all autistic suicide death is in childhood and early adulthood”.** Most comments within Theme 1 (68.9%) were from participants expressing a preference for long-term actions which would prevent suicide in future. Many explained that irreparable harm caused by bullying and unmet needs in their formative years had set the “seeds” of suicidal thoughts: suicide prevention therefore necessitated breaking the trajectory of trauma, every-day struggles and hopelessness which would otherwise render crisis interventions ineffective. Other participants framed long-term actions in relation to increasing resilience in young autistic people and increasing their opportunities to access things that “mak[e] life worth living”.

**“Crisis 1st, preventing crisis 2nd, quality meaningful satisfying life 3rd”.** Relatively fewer participants (16.4%) expressed clear preference for indicated interventions, relegating preventative actions until those in crisis had been helped. They often mentioned interventions which had helped them, including non-verbal crisis support and safe retreats.

#### Theme 2: “brutalised, broken systems”—the logic of suicide and the need for “radical social change”

The diverse comments interpreted within this superordinate theme bore similarities to experiences and ideas reported by participants from Phase 1 of the survey,^9^^,^^10^^,^^20^ but this larger sample's completion of the ranking exercise afforded them different opportunities for reflection. Comments represented in this theme conveyed the scale of societal change needed to prevent suicide, highlighting failures of “the system”, “structures”, “infrastructure”, “services”, “the world” and “society”. Suicide was frequently positioned as a logical, rational and inevitable consequence to these circumstances; some participants expressed apparent hopelessness, anger at government inaction and hypocrisy. While they did not explicitly weigh indicated, selective or universal approaches, these comments are implicitly supportive of actions beyond the mental health sector which target autistic people broadly and conceptualise their suicide in relation to social inequities rather than psychopathology.

In addition to reflections on the ‘system’ broadly (14.2%), the remaining comments under this theme (85.8%) mentioned one or more specific supports, services or needs. These were interpreted as subthemes reflecting presently unmet needs: for acceptance, belonging and community; accessible, appropriate and trustworthy healthcare and crisis services; access to diagnosis and post-diagnostic care; suitable educational provision and transition support; suitable employment and social care in the community; financial security; safety from victimisation and equity within the criminal justice system; and equality for diverse identities and needs, such as those of autistic girls, women and gender-divergent individuals, people of colour, and people with co-occurring ADHD (Attention Deficit Hyperactivity Disorder). Within these subthemes were responses which emphasised that support and services must be *proactive*, *flexible*, *accessible* (particularly without speech), and *specific* for autistic people.

### RQ2: attitudes towards provision of autism-specific support to people who think they might be autistic

**Fig. 2:**
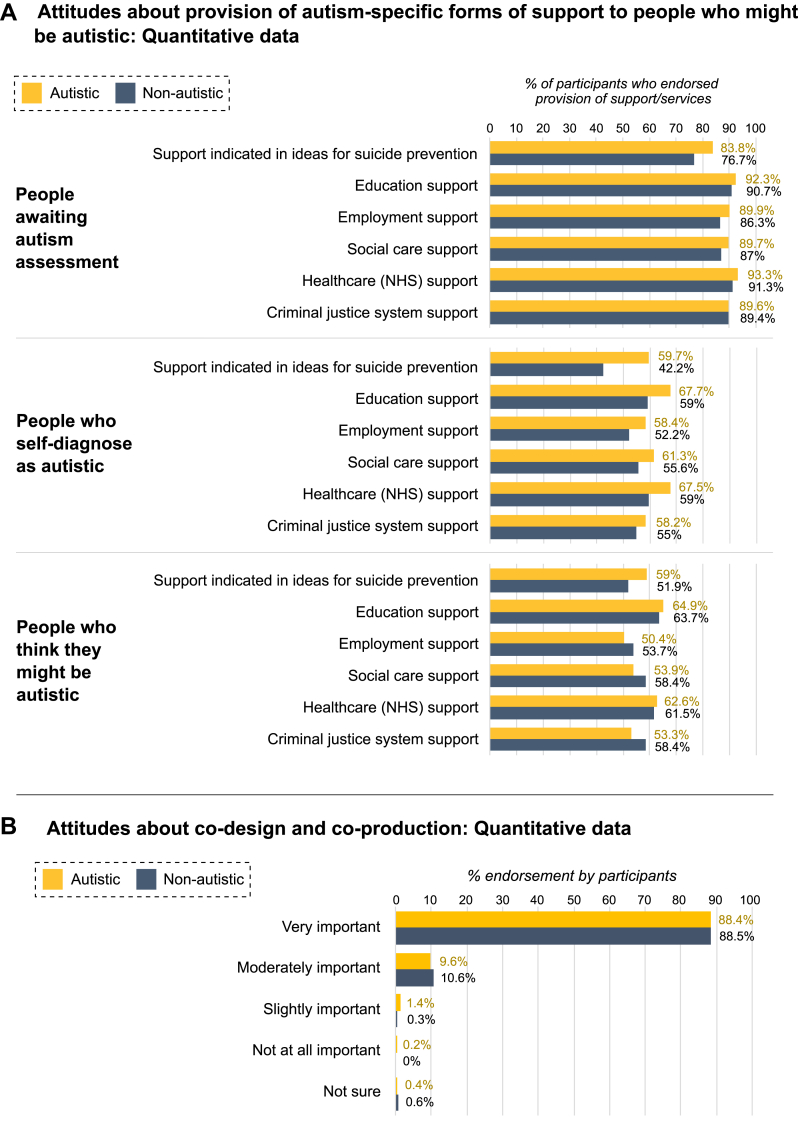
*Attitudes about provision of autism-specific support to people who might be autistic (A), and co-design and co-production (B)*. *Note*. Fig. 2 Part A depicts participant responses to closed-ended questions about provision of autism-specific support to people awaiting autism assessment, people who self-diagnose as autistic, and people who think they might be autistic. Note that to obtain endorsement for each category, percentages include the opinions of participants who selected ‘all of these groups’ (a response choice was chosen by almost 50% [47.9%] of autistic and 32% of non-autistic participants in relation to ideas for suicide prevention; by 55.6% of autistic and 51.9% of non-autistic participants in relation to education; by 44.5% of autistic and 43.8% of non-autistic participants in relation to employment; by 47.6% of autistic and 48.4% of non-autistic people in relation to social care; by 53.9% of autistic and 51.9% of non-autistic people in relation to healthcare; and by 47.3% of autistic and 48.8% of non-autistic participants in relation to the criminal justice system). In the instance of ideas for suicide prevention, an additional option, expressing uncertainty or preference not to disclose, was chosen by 4.2% of autistic and 6.5% of non-autistic participants. Part B depicts responses to the closed-answer question about co-design and co-production.

While participants typically endorsed provision of autism-specific forms of support to individuals awaiting autism assessment, they were more divided in relation to individuals who self-diagnosed as autistic or thought they might be autistic (see Fig. 2, Part A).

Diverse opinions in the qualitative data were interpreted as two themes (see Table 3; Supplementary Materials, 5).

**Table 3:** Themes and subthemes related to provision of support to undiagnosed individuals.

Themes, *subthemes*	Quotations from autistic participants and non-autistic supporters/allies
**1. “Until access to diagnosis is universal, discrimination against self-diagnosis is unethical ”** This theme reflected statements endorsing the need to make all supports and services for autistic people inclusive of those at *every* stage of the diagnostic process (including those querying an autistic identity and/or self-identifying as autistic).	“I chose all, ‘cos “thinks they might be autistic, self diagnosed, awaiting assessment, diagnosed” is a natural sequence followed by any adults who end up diagnosed” (autistic non-binary individual, 58, diagnosed, England) “Until access to diagnosis is universal discrimination against self diagnosis is unethical” (autistic cisgender man, 49, diagnosed, England) “I think support should be available on symptoms basis, not just diagnosis basis. Do you have these symptoms, and would this service help? Then you should be entitled to help” (autistic cisgender woman, 26, undiagnosed, Scotland) “I think having some things available to anyone who may need it is infinitely better than not having them at all. Accessibility can help everyone (like the drop curb effect)” (autistic cisgender woman, 26, undiagnosed, England) “I know undiagnosed autistics not on a waiting list. A lot of them are (eg) scared of doctors, or were denied being put on a list, or fear it would affect child custody so avoid diagnosis” (autistic cisgender woman, 50, diagnosed, England) “Because of delays in diagnosis and lack of diagnoses in women and minority groups, anyone who thinks they have autism and that it is impacting their ability to cope must be able to access resources” (autistic cisgender woman, 44, diagnosed, England) “I understand clinical diagnosis confirms presentation. ADULTS that receive late diagnosis late are born autistic and have faced many difficulties without the right support. Neuro diverse needs shared” (autistic cisgender woman, 47, diagnosed, England) “We can know we are autistic, whether formally diagnosed or not. We can't make people wait 4 years for a certificate That wait can change functioning to actively suicidal simply due to lack of support” (autistic cisgender woman, 56, diagnosed, England) “I believe too much emphasis is placed on a piece of paper to prove diagnosis. My brother told mental health services that he thought he may be autistic but this was simply ignored” (non-autistic bereaved supporter/ally, cisgender woman, 44, England)
**2. “A diagnosis should be the gateway to services”** This theme reflected statements wherein participants recommended that formal diagnoses remain the point of access to receiving any support or services designed for autistic people.	“With diagnosis or on waiting list assumes a definite or potential ASD *[author note: autism spectrum disorder]*. Other folks could have other conditions that would not necessarily be helped the same” (autistic genderqueer individual, 64, diagnosed, Scotland) “If someone meets the criteria to be referred then they should be seen as on a waiting list if their GP takes them seriously or not. Self diagnosis could be dangerous” (autistic genderfluid individual, 35, diagnosed, England)
***i. “Nobody should be in a position where they can't obtain a formal diagnosis”*** This subtheme reflected comments where participants prioritised quick and accurate identification, assessment and diagnosis of individuals missed for diagnosis in childhood (and subsequently supporting them), rather than providing support to undiagnosed individuals who might or might not be autistic.	“A lot of these questions relate to the slow NHS diagnose of autism. A diagnose should be the gateway to these services, if only older people could get one” (autistic cisgender man, 69, undiagnosed, England) “Better access to diagnosis [would] remove need for self diagnosis” (autistic intersex individual, 71, diagnosed, England) “Self diagnosis is valid, but for society to understand we need more diagnoses. Disabled people are so often vilified so I have little faith we can make a difference unless it's strategic” (autistic cisgender woman, 31, undiagnosed England) “I think we should focus on radically improving diagnostic processes rather than redesigning entire institutions to accommodate undiagnosed people” (autistic gender-questioning individual, 33, diagnosed, England) “Focus on reducing waiting times for diagnosis, rather than stretching support for all the above to everyone who thinks they MIGHT be autistic” (autistic cisgender woman, 42, diagnosed, England)
***ii. “Services are already stretched”*** This subtheme reflected comments where participants expressed concerns over service capacity as a reason for limit support to individuals without formal autism diagnoses.	“I think access should be restricted to provide focus and make sure it is available for those with a diagnosis, rather than trying to do it for too many and services getting overwhelmed” (autistic cisgender male, 53, diagnosed, England) “Conflicted about question referring to whether services should be available to those not formally dx'd *[author note: diagnosed]*. With limited resources I put formal DX only, but recognise there are barriers to formal DX” (autistic cisgender woman, 68, diagnostic information not provided, England) “I believe that given limited resources (Either NHS, Education, Work, etc) people who are formally diagnosed or are awaiting a diagnosis should be prioritised. Otherwise systems could get overwhelmed” (autistic cisgender man, 25, diagnosed, England) “The ONLY reason I think self-diagnosed/“might be” shouldn't get Employment/CJS support is saturation would stop those who DO need it getting it” (autistic transgender woman, 50, diagnosed, England) “Formal diagnosis more likely to happen when someone is really struggling. I was diagnosed via nhs. There is however a lot of misinformation on social media about ND *[author note: neurodivergent]* & services are already stretched” (autistic cisgender woman, 40, diagnosed, England)
***iii. Self-diagnosis “muddies the water”*** This subtheme reflected comments where participants expressed anxiety and scepticism towards the practice of self-identifying as autistic, for instance due to fears of exploitation, or that the needs of diagnosed autistic people might be underestimated.	“I just feel there are people who will and have abused the system claiming ASD to behave inappropriately and get away with things. So I believe it's a delicate area” (autistic cisgender woman, 39, diagnosed, England) “Self diagnosis … makes those of us with a diagnosis seen as skivers because we “made it up” Yes I have been told that, many times” (autistic cisgender woman, 59, diagnosed, England) “People should not be allowed to self diagnose it muddies the water and makes it more difficult for those of us who are diagnosed” (autistic cisgender woman, 59, diagnosed England) “I feel the term autistic is becoming meaningless, or at least no longer represents people with severe autism. The very people that need the most help are not having their needs considered” (non-autistic supporter/ally, cisgender woman, 50, England) “As someone who waited 3 years for my diagnosis through the NHS I've also noticed some people believe autism is fashionable. I do not like the idea of self diagnosis. There is a process !” (autistic cisgender man, 42, diagnosed England)

Note. Quotations from autistic participants and non-autistic supporters/allies as pertaining to provision of support for undiagnosed individuals. Quotations from autistic participants are inclusive of formally diagnosed and possibly autistic individuals. Acronyms used by participants are explained by italicised author notes.

#### Theme 1: “until access to diagnosis is universal, discrimination against self-diagnosis is unethical”

Approximately one third of comments on this topic (36.6%) stated the need to make support designed for autistic people available on a needs-basis, inclusive of people at all stages of the journey to a formal autism diagnosis, including self-diagnosis. Inclusivity was necessitated by long wait times, barriers to diagnoses affecting certain groups, fears that might prevent individuals seeking diagnoses, and the lifetime struggles and suicide risk of undiagnosed autistic people.

#### Theme 2: “a diagnosis should be the gateway to services”

Remaining (63.4%) comments related to this issue were from participants expressing views that diagnosis should remain the point of access to autism-specific support. Frequently, participants extended access to individuals awaiting diagnostic assessment, with some indicating that professional referrals bestowed some legitimacy to their suspected autism. Three subthemes reflected different reasons for preventing access to those who self-diagnosed or thought they might be autistic.

**“Nobody should be in a position where they can’t obtain a formal diagnosis”.** Approximately a quarter (26.6%) of comments within Theme 2 were from participants who prioritised “radical improv[ement]” and expedition of autism assessments over extension of services to undiagnosed people. Some expressed that self-diagnosis was valid but less than ideal, with one suggesting that formal diagnoses helped society better understand autistic people. Others suggested that improved assessment would remove the need for self-diagnosis completely.

**“Services are already stretched”.** Comments in this subtheme (14.3% of comments within Theme 2) reflected concerns about service capacity, often while expressing “mixed” or “conflicted” feelings about setting limits. Some noted that provision of support to undiagnosed people would detract from that available for diagnosed individuals.

**Self-diagnosis “muddies the water”.** The remaining (59.1%) comments within Theme 2 were those expressing anxiety or scepticism towards the validity of self-diagnosis. Some expressed fear that self-diagnosis might facilitate exploitation or abuse, which could also smear public perceptions of autism and autistic people. Others worried that the “fashionable” claiming of autistic identities might obscure and minimise the challenges of diagnosed individuals; and that inclusion of self-diagnosing individuals would detract from the benefit of autistic spaces and resources. Inherent in many responses was the perception of “a/the process” (formal diagnosis) which self-diagnosing individuals should “be prepared to go through”. A small number of participants expressed concerns *for* self-diagnosing individuals who might receive inappropriate support because of “mis-diagnosing”.

### RQ3: attitudes towards co-design and co-production

The quantitative and qualitative data indicated that most participants felt co-design and co-production were very important (see Fig. 2B). We interpreted two themes in comments related this topic (see Table 4; Supplementary Materials, 5).

**Table 4:** Themes and subthemes related to co-design and co-production of suicide prevention efforts

Themes, *subthemes*	Quotations from autistic participants and non-autistic supporters/allies
**1. “Nothing about us without us”** This theme reflected comments where participants highlighted the importance of co-design and co-production in straight-forward terms, without necessarily expounding on why these were important.	“I am aware of the power of peer support. I believe autistic people should be involved at every stage including design and running services. This is genuine co-production not tokenistic involvement” (autistic cisgender woman, 58, undiagnosed, England) “Autistic people should be involved in the creation of improvements and creation of services for autistic people at every step” (autistic transgender man, 26, undiagnosed, England) “Being autistic I need to be involved in design of anything that is going to affect how I do something, it enables me to process the information better, allows me to do in a way I understand” (autistic cisgender woman, 50, diagnosed, England) “The autistic experience varies between individuals, having an input from all reaches of the spectrum would help develop more holistic strategies” (autistic non-binary individual, 27, diagnosed, England)
***i. “Nobody knows better than we do about ourselves”*** This subtheme reflected comments where participants highlighted that the knowledge and ‘insider perspective’ of autistic people was an essential ingredient in suicide prevention strategies.	“NT *[author note: neurotypical]* people have no idea what life is like for an autistic or ND person can they understand and develop things that can work for a ND person. Systems need to be made by the users or it just won't work” (non-autistic bereaved supporter/ally, cisgender woman, 45, England) “Absolutely no point a NT person giving NT advice to a ND person … our brains don't work the same way” (autistic cisgender woman, 47, diagnosed, Scotland) “You need autistic people involved else how do neurotpicals know they are doing it right? They may understand the theory, the practical is very different” (autistic cisgender man, 54, undiagnosed, England) “Even those who are neurotypical and have a son who is autistic are not ever going to be entirely tuned into daily experience of being autistic and will always have an outsider's perspective” (autistic non-binary individual, 24, diagnosed, England) “Autistic individuals are extremely good with understanding complex needs and situations and should be involved at every level about deciding what happens next” (autistic cisgender man, 64, diagnosed, England) “I don't really feel you can understand Autism unless you experience a lifetime of discrimination, not heard, seen or noticed” (autistic cisgender woman, 63, undiagnosed, England)
***ii. “We need to feel understood in crisis”*** This subtheme reflected comments where participants highlighted that autistic people could offer a level of emotional understanding and empathy which non-autistic people might not otherwise be able to provide.	“I want help from people who get it. Last year I got bullied by someone from [specific service redacted]. It was meant to be a triage interview he made my situation worse due to lack of understanding” (autistic cisgender woman, 31, diagnosed, Wales) “Non-autistic people will never understand despite training, we need autistic people trained to help so we really feel heard” (autistic cisgender woman, 55, diagnosed, England) “It takes one to know one–who better to deal with our complex needs than our own kind? We need to feel understood in crisis” (autistic cisgender woman, 60, diagnosed, England) “We need the empathy and understanding we can only really get from other neurodivergent people” (autistic cisgender woman, 34, undiagnosed, England) “It is important to provide support from those that autistic people identify most with” (autistic cisgender woman female, 36, undiagnosed, England)
**2. “Caution”—the need for diverse representation** This theme reflected comments where participants expressed limits to the principles of co-design and co-production, and/or expressed concerns about the same. The majority of concerns related to certain views and needs being prioritised over others. Some pointed out the added value of, and need for, non-autistic perspectives.	“Having a great input from autistic people in the design and running of the support is inevitably going to result in it being geared towards more higher functioning [sic] individuals” (non-autistic supporter/ally, cisgender woman, 50, England) “I have limited my support for co-production and peer-mentoring because ‘activists’ who get involved in this may not always represent other autistic people well” (autistic cisgender woman, 45, diagnosed, England) “I think that those who have experience are the best sources of information, however caution. Because there are many who only seek their own benefits, we must support our community needs wisely.” (autistic cisgender woman, 37, diagnosed, England) “There is such a wide range o [f] autism severity and experiences that in fact professionals who have seen a wider range of autistic expressions and challenges might be more helpful to design” (autistic cisgender woman, 42, undiagnosed, England) “Having a suicidal person with autism affects everyone in family. I feel that everyone who is affected should have a say. My son is very ill and it's hard for him to have insight” (non-autistic supporter/ally, cisgender woman, 52, England) “I think things shouldn't always be led by those close to it. I also had to have IVF. You couldn't trust infertile people to run the ethics committees–they're just too close to it to step back” (autistic cisgender woman, 51, diagnosed, England)

Note. Quotations from autistic participants and non-autistic supporters/allies as pertaining to co-design and co-production of suicide prevention efforts Quotations from autistic participants are inclusive of formally diagnosed and possibly autistic individuals. Acronyms used by participants are explained by italicised author notes.

#### Theme 1: “nothing about us without us”

Most comments about co-design and co-production (92.3%) spoke strongly of the importance of both, highlighting the need to involve diverse representatives of the autistic community and avoid tokenism. Some comments indicated the *nature* of the benefits yielded by co-design and co-production, such as feelings of trust and investment in the support, services or intervention. Two kinds of benefits were present in the data as two subthemes:

**“Nobody knows better than we do about ourselves”.** The most frequently mentioned strength of co-production was the knowledge and understanding of autistic people, sometimes framed in contrast to a *lack* of knowledge and understanding in non-autistic people, sometimes framed in relation to *advantages* bestowed by autistic thinking styles and shared experiences.

**“We need to feel understood in crisis”.** Less frequently, participants identified socioemotional connection and empathy afforded by autistic involvement in suicide prevention efforts, reflected in expressions that participants would “feel heard”, “feel understood” and be able to identify with autistic people involved in service delivery.

#### Theme 2: “caution”—the need for diverse representation

The remaining comments related to co-design and co-production (7.7%) raised concerns that outcomes might not reflect the needs of subgroups who are often excluded from co-production, such as autistic people with substantial support needs, those who are non-speaking and/or have learning disabilities. Two participants expressed concerns about the capacity for insight in people experiencing mental health and/or suicidal crises, and/or the objectivity of those with personal experience of suicide. One pointed out that affected family members should also have a voice.

## Discussion

Since suicide prevention strategies should be informed by the needs and desires of the priority groups they aim to serve,^15^ we sought to understand the attitudes of autistic people and their supporters/allies with regards the balance of crisis interventions to preventative measures (RQ1); provision of autism-specific supports to people without formal diagnoses (RQ2); and the importance of co-design and co-production (RQ3). We contextualise their responses in relation to data presented in our accompanying paper^20^ and other published work.

Participants had been exposed to diverse ideas for suicide prevention, spanning universal (eg tackling mental health stigma) to indicated approaches (eg safe retreats for autistic people in crisis).^20^ Our first research question (RQ1) pertained to their preferences with regards to suicide prevention actions along this continuum of approaches. Crisis interventions of the ‘indicated’ type have traditionally been the mainstay of government approaches to suicide prevention,^21^ but systemic inequalities and structural barriers may prevent their reaching people who need them. This is particularly pertinent in an autism context, given barriers to healthcare and diagnosis faced by women, gender and ethnic minority groups.^22^^,^^33^ Perhaps in relation to such experiences, the selected priorities of our participants appeared to be slightly weighted towards longer-term ‘selective’ interventions spanning education, employment and social care, aimed at disrupting the early origins of suicidal thoughts and feelings. While their qualitative data confirmed the necessity of crisis interventions, preference for longer-term actions was corroborated and explained in relation to the “irreparable harm” accrued from childhood into emerging adulthood, for instance, through bullying at school, insufficient educational and employment support, and growing up undiagnosed. Many placed particular weight on childhood, corroborating the early emergence of suicidal thoughts and behaviour in this group.^3^^,^^4^ Where autistic and non-autistic participants alike linked suicidal thoughts and behaviour to well-documented, life-long social and economic inequalities faced by autistic people,^8^^,^^17^ their responses mirrored those of autistic participants in our earlier work,^9^^,^^10^ as well as autistic people interviewed by other research groups.^11^ Our participants' focus on pre-suicidal stages in the suicidal trajectory was also consistent with the emphasis participants in the 2021 priority-setting exercise^16^ placed on identifying risk and protective factors across the lifespan. Our findings expand these concordant notes with a specific focus on policy and practice. While recognising the necessity of indicated approaches for people in crisis, both autistic and non-autistic participants ultimately corroborated public health approaches^15^^,^^21^ with regards the need to focus suicide prevention efforts upstream from crisis point, addressing the systemic inequalities which contribute to suicide and excess morbidity in this population.^6^, ^7^, ^8^, ^9^, ^10^^,^^17^

A shift towards longer-term preventative actions is already realised in national and international policy,^12^^,^^34^ but is thus far lacking in an autism context. In UK suicide prevention policy for autistic people,^12^ for instance, the most focal and concrete action concerns one indicated intervention for safety planning,^19^ without implications for policies affecting broader aspects of autistic lives. This highlights a fundamental need to challenge prevailing narratives about mental illness and suicide in autistic people, which have traditionally been “individualised, ‘internalised’, pathologised [and] depoliticised”.^35^ In contrast, this and the earlier Phase 1 sample^9^^,^^10^ both situated suicidal thoughts and behaviour in the context of chronic societal exclusion, injustice and inequality. Some participants in the present analysis, moreover, explicitly asserted that suicide was rational in the context of their or their autistic loved one's lives. This perspective is not new. Suicidal thoughts and behaviour outside of the context of mental illness was a strong theme of the 2021 priority-setting exercise with autistic people and their supporters/allies.^16^ Other disabled and lived experience voices similarly challenge the automatic assumption that suicidal thoughts and behaviour occur in circumstances of diminished mental capacity.^36^

This message provides an opportunity to do precisely what participants in the 2021^16^ and our own priority-setting exercise^20^ requested: *listen* to autistic people. Only by acknowledging this strong, consistent and disquieting message can we adopt more effective mindsets and approaches to the burden of suicidal thoughts and behaviour in this group. In fact, where our participants explicitly linked the “logic” of suicide to systemic inequalities, this highlights the relevance of critical suicide frameworks,^35^^,^^37^ which call attention to the sociocultural and economic conditions under which suicide disproportionately affects marginalised communities. Critical suicide scholars^37^ concur with those in public health^21^ that indicated interventions in the clinical-psychiatric domain are unlikely to yield the most significant reductions in suicide deaths, given insufficient capacity of services and access barriers affecting the marginalised groups at highest risk. Responses from participants in our study similarly suggest that automatically situating suicidal thoughts and behaviour in autistic people in the associated context of individual mental health crises, perhaps as per historic medicalisation of autism,^17^ unfairly robs them of their socioeconomic and political context, and may preserve the status quo of “playing whack-a-mole” (reacting to crises when they occur, rather than preventing them). Indubitably, the varied circumstances of autistic people who experience suicidal thoughts and behaviour mean that a focus on mental illness will be appropriate for some. However, even this demands scrutiny of their circumstances and environment,^6^^,^^7^ so moving past narratives of individual psychopathology, and narrow focus on indicated approaches, is vital.

In our second RQ, we sought to understand the perspectives of our participants as regards the availability of support and resources to people who think they might be autistic. In light of “devastating”^38^ waiting times for assessment and the possibility of over one million undiagnosed individuals in England alone,^25^ the case for extending autism-specific support to individuals who think they might be autistic had appeared to us unassailable. Personal narratives are indicative of extreme distress in undiagnosed individuals,^39^ who are over-represented in populations who consider, attempt or die by suicide.^26^^,^^30^ Self-diagnosis is a common step on the trajectory to an adult diagnosis,^31^ and exclusion of these individuals would perpetuate inequalities for those less likely to receive diagnosis.^22^ As such, we were initially surprised, in the quantitative data, that while autistic and non-autistic participants generally endorsed providing autism-specific supports to individuals awaiting diagnostic assessment, both groups were far more divided regarding extending this provision to self-diagnosing individuals and those querying possible autism.

This finding appears contradictory to the solidarity undiagnosed individuals report in autistic spaces,^40^ and the role of undiagnosed autism in the suicidal experiences of the present and the Phase 1 sample.^10^^,^^20^ The qualitative data revealed that while just over a third of participants asserted the aforementioned reasons for supporting people who self-diagnose or think they might be autistic, just over one quarter (27%) preferred expediting diagnosis for these individuals, viewing this as the appropriate route through which they should receive help. Problematically, keeping support contingent on diagnosis would exclude individuals with different, valid reasons for avoiding formal assessment, as well as those who face barriers to diagnosis.^22^^,^^31^

Other attitudes about self-diagnosis, expressed by autistic and non-autistic people alike, appear similarly driven by fear and anxiety, sometimes overt in relation to service capacity or abuse, sometimes implicit in perceptions of personal detriment to participants' own identities and needs (or those of their loved ones). As per recent findings about self-diagnosis of mental health conditions,^41^ some participants felt that “fashionable” self-diagnosis “invalidated” their own reality and affected how others treated them. These attitudes also appear reflective of internationally-expressed anxieties which have, among other consequences, led to proposed divisions like ‘profound autism’.

These findings are the first, to our knowledge, concerning community attitudes towards people who self-diagnose. It is essential that they are contextualised by sample demographics and participant experiences: those who self-identified were a minority here. The majority of our ‘possibly autistic’ group were on the diagnostic pathway, and the majority of formally diagnosed autistic people were diagnosed in adulthood, having presumably traversed this often lengthy and challenging route. The majority of supporters/allies, too, were associated with formally diagnosed autistic people (though formal diagnoses were, sadly, considerably lower in the individuals described by those bereaved by suicide). This may partially explain participants' adherence to formal pathways for diagnosis. As such, these views may not be wholly representative of the wider autism community.

Beyond the issue of representativeness, the divisions we observed among our participants must also be vitally contextualised by resource scarcity. Responses seem clearly reflective of the hypervigilance of a marginalised group against individual and resource threat in the context of limited resources, and may demonstrate the circumstances under which community solidarity frays. Participants acknowledged that, given high risk of suicide in undiagnosed individuals,^26^^,^^30^ suicide prevention measures targeting people at different stages of the diagnostic journey are imperative, and must go beyond bestowing an autism diagnosis to providing post-diagnostic (or post-realisation) support.^20^ Extension of autism-related support to undiagnosed individuals must be sensitively implemented to avoid real and perceived detriments within the community, and is another argument for partnership with the community.

Our third RQ pertained to the role of co-design and co-production in interventions and initiatives to prevent suicide in autistic people. Given that actions within the continuum of approaches to suicide prevention will differentially benefit subgroups within the autistic community, inclusion of diverse viewpoints appears imperative to ensure consideration of expansive, multilayered strategies. In the first phase of this survey, a key role for the autistic community in suicide prevention was indeed indicated by prioritisation of formal and informal peer support over other strategies.^20^ The present, Phase 2 participants almost unanimously agreed the importance of autistic involvement in efforts towards suicide prevention, corroborating broader calls for co-produced health interventions for autistic people.^16^ Our third RQ pertained to understanding their attitudes towards community involvement in greater depth, and revealed diverse opinions about the extent to which power should be balanced between community and non-community members: some spoke of more minor “consultation” with autistic people, others to efforts being “led by” autistic people. Nevertheless, some participants noted valid concerns, voiced elsewhere,^42^ that warrant inclusion of non-autistic perspectives and thoughtful planning to ensure representation of diverse needs.

Given that misunderstood and unmet needs affect the mental health of autistic people in education, employment, social care and healthcare settings,^8^^,^^10^^,^^17^ our participants highlighted the value of unparalleled knowledge through experience, coupled with an empathic relatedness perhaps akin to that autistic people find from autistic peers.^43^ Co-produced or community-led approaches may also facilitate social connectedness and belonging in individuals via identification with the community.^44^ This suggests that authentic autistic experiences of suicidal thoughts and behaviour must be at the heart of service delivery and adapted interventions.

There are a number of important limitations or caveats to this work. Participants’ attitudes inherently reflect UK culture and may not reflect international, and particularly low-resource, contexts. Even in high-income settings, they may not reflect the perspectives or opinions of autistic people with minority and/or disadvantaged racial, ethnic, socioeconomic and cultural backgrounds; autistic cisgender men, sexual and gender minorities; autistic samples with lower educational attainment and employment rates; and those with learning disabilities, who are often excluded from efforts towards co-design and co-production.^42^^,^^45^ Indeed, the situation of the questions relevant to this analysis at the end of an otherwise complex task^20^ may have biased our sample: certain participants, such as those with lower computer literacy and/or reading levels, poorer physical health, and/or co-occurring forms of neurodivergence (like ADHD and dyslexia), may have been less likely to reach and complete this part of the survey. In that most of our participants were indeed highly-educated individuals diagnosed in adulthood, these facts are strongly pertinent to their experiences of current services and systems, and their perspectives regarding each of our RQ. Many would have navigated education, employment, and health and social care while undiagnosed; while these environments appear inhospitable to diagnosed and undiagnosed autistic people alike, the experiences of diagnosed and undiagnosed individuals are likely to differ in significant ways. Moreover, seeking diagnosis in adulthood, participants may have needed to navigate a more complex, individually demanding process, and had greater awareness of its deficiencies; at the same time, the likely value of such diagnoses, and the challenge of seeking them, may, as mentioned, have influenced views on provision of services and support to undiagnosed people who might be autistic. As such, on this and other issues, the perspectives of formally diagnosed participants herein may not be shared by other formally diagnosed autistic people, such as those diagnosed in early childhood.

As pertains to the supporters/allies in this study, this group was fairly homogeneous–predominantly parents, almost all describing relationships with formally diagnosed autistic people without learning disabilities. With few representatives of individuals bereaved by suicide and specific stakeholder groups, like professionals, friends and partners, their perspectives are therefore likewise unlikely to be shared by all who identify as supporters and/or allies of autistic people. The relative smallness of the supporter/ally group prevented differentiation of autistic and non-autistic views, and nor did we systematically or statistically investigate, in autistic people or supporters/allies, factors likely to have influenced attitudes towards the topics of our RQ. For example, educational attainment, socioeconomic and employment status could plausibly influence attitudes surrounding fears of resource scarcity (pertaining to undiagnosed people who might be autistic), as could the age and gender of the participant, in addition to their own diagnostic experience (or that of the person they advocated for). Similarly, participant exposure to different services and their experiences of the same likely influenced their views in ways not captured here: for example, negative experiences of crisis interventions may have driven our participants’ preference for longer-term preventative actions. This underscores the need for caution as regards the transferability of these findings to other autistic people and supporters/allies, as well as highlighting important considerations for further study of these issues.

Participant views were sought within the constraints of a longer survey, where participants had thus already been exposed to diverse ideas spanning indicated to universal approaches.^21^ We inferred their attitudes about the staging of prevention efforts through comments influenced by these ideas; relatively greater ease of identifying longer-term preventative actions vs. specific indicated approaches may have influenced the weighting of their responses towards the former. While we asked directly about provision of autism-specific support to people who might be autistic, and about co-design and co-production, additional content might have been yielded by separate qualitative questions addressing the three topics. There is need for more nuanced research that explores the ideal roles of community and non-community members in specific actions, as well as optimal means of monitoring and evaluating these.

Finally, while trying to ensure data integrity, we were unable to ratify information provided herein. Our anonymous survey facilitated openness about potentially contentious issues, but varied approaches should be taken to hear views from individuals less likely or able to respond to online surveys.^42^

These limitations notwithstanding, we conclude our work with its implications for policy and practice. The high incidence of suicidal thoughts and behaviour in our participants, against the backdrop of previous empirical work and population data,^2^^,^^3^^,^^9^^,^^10^^,^^14^^,^^16^^,^^18^^,^^26^^,^^30^ demonstrate incontestable need for urgent action.

Chiefly, our findings indicate the need for policymakers to understand and address autistic suicide risk in a broader, de-individualised context. Participants highlighted known social determinants to suicide which are highly relevant in an autism context, including poverty, marginalisation, and insufficient health and social care.^6^, ^7^, ^8^, ^9^, ^10^^,^^17^ They also highlighted factors more specific to neurodivergent and disabled people, including inaccessible workplaces, the “dehumanising” benefits system, barriers to diagnosis and lack of post-diagnostic support^10^^,^^20^—factors which justify tailored approaches to this and other priority groups.

Specific, actionable strategies for suicide prevention, as identified by this sample, are detailed in our accompanying paper^20^ and other forthcoming work.^46^ With the present paper offering a more detailed qualitative investigation of participant priorities, it is clear that they deemed both crisis interventions *and* longer-term preventative measures imperative. Specifically, participants highlighted that crisis measures are needed for individuals in or approaching crisis, many of whom will be late- or undiagnosed. Rather than specialised services contingent on diagnosis, increasing the confidence and capacity of mental health professionals to recognise and work in a needs-based way with autistic people, undiagnosed autistic people (regardless of awareness of their autism), people with autistic traits and broader sociocommunicative differences could effectively support those without formal diagnoses while addressing community anxieties about service resources.

Notwithstanding the necessity of such measures, the longer-term preventative actions favoured by participants demand long-term vision, committed resources, and expansive, coordinated responses across sectors to quickly identify autistic children and provide sustained, appropriate support across their lives, reducing excess morbidity and mortality in this population.^2^ From a different perspective, participants' ideas and reflections challenge key decision-makers to create conditions in which autistic people can live equally rewarding, meaningful and dignified lives. The present paper, and the accompanying paper documenting participants’ ideas,^20^ demonstrate the inarguable competency and readiness of the autism community to contribute to problem-solving in this area. We urge researchers, practitioners and policymakers to take next steps towards developing partnerships in which diverse community members, including those often excluded from co-production activities, can take leading roles in developing specific action plans for suicide prevention. Accessible and expansive approaches to co-production are imperative to ensure as diverse a range of contributors as possible, and thus develop multilayered strategies.^42^^,^^45^

In conclusion, a sea change in thinking is required to achieve substantial reductions in the number of autistic people who consider and die by suicide; a de-individualising and recontextualising recognition of suicidal distress in the context of systemic injustices, and a related shift towards coordinated, preventative measures, properly resourced, which disrupt the trajectory to suicide across the lifespan. Partnership with the community is vital to ensure that needs-based support is enriched by insider knowledge and empathy, and that measures and approaches are appropriate for autistic individuals.

## Contributors

Conceptualisation and design (SBC, CLA, TAP, JC, RLM, SC, DM, IH, LO, DC, JR); data acquisition, analysis and/or interpretation (all authors; however, RLM and SJM accessed and verified the underlying data); online advertising (TAP); drafting (RLM) and reviewing the work (SJM, MP, EW, TP, CLA, TAP, CS, TC, HH, DM, JR, IH, LO, JC, DC, DH, SBC). All authors read and approved the final version to be published and accept responsibility to submit for publication.

## Data sharing statement

We did not seek consent from participants to make our data publicly available. However, we welcome reasonable requests for access. We also note that the quotations used in our analyses are available, albeit without identifying information, within our Supplementary Materials.

## Declaration of interests

RLM declares no competing interests. SJM declares no competing interests. MP declares current employment supported by charity Autism Action, received research grants from MindEd Trust and Edinburgh Mental Health Networks in 2024, and, in 2025, received grants to attend IASP World Congress from both the British Psychological Society and British Psychological Society West Midlands division. EW declares no competing interests. TP declares no competing interests. CLA declares no competing interests. TAP is an employee of Autism Action (previously the Autism Centre of Excellence at Cambridge), who initiated the project and funded the online promotion of the survey used in this research. SC reports previous grants from NIHR, Autistica, ESRC, Chief Scientist Office, International Society for Autism Research; occasional honoraria for lectures/workshops at conferences/educational events; Occasional expert witness for courts and coroners offices during inquests; and being a participant on DMEC for NIHR funded RCT trial, and currently chairing an advisory board for a current NIHR grant. TC declares no competing interests. HH declares no competing interests. DM declares no competing interests. JR declares no competing interests. IH is a trustee of Autism Action (previously the Autism Centre of Excellence at Cambridge), who initiated the project and funded the online promotion of the survey used in this research. LO is a trustee of Autism Action (previously the Autism Centre of Excellence at Cambridge), who initiated the project and funded the online promotion of the survey used in this research. JC is an employee of Autism Action (previously the Autism Centre of Excellence at Cambridge), who initiated the project and funded the online promotion of the survey used in this research. DC declares no competing interests. DH declares no competing interests. SBC is trustee of Autism Action (previously the Autism Centre of Excellence at Cambridge), who initiated the project and funded the online promotion of the survey used in this research. SBC received funding from the Wellcome Trust 214322∖Z∖18∖Z. SBC also received funding from the Innovative Medicines Initiative 2 Joint Undertaking under grant agreement No 777394 for the project AIMS-2-TRIALS. This Joint Undertaking receives support from the European Union's Horizon 2020 research and innovation programme and EFPIA and AUTISM SPEAKS, Autistica, SFARI. SBC also received funding from Autism Action (previously the Autism Centre of Excellence [ACE] at Cambridge), SFARI, the Templeton World Charitable Fund and the MRC.
