# Supplementary material for: “The best way we can stop suicides is by making lives worth living”: a mixed-methods survey in the UK of perspectives on suicide prevention from the autism community
Source: eClinicalMedicine. 2026 Mar 3;93:103793. doi: 10.1016/j.eclinm.2026.103793 (PMC13043318; doi:10.1016/j.eclinm.2026.103793)
Supplement: Supplementary Materials [file mmc1.docx]

**Supplementary Materials for**

***“The best way we can stop suicides is by making lives worth living”: a mixed-methods survey in the UK of perspectives on suicide prevention from the autism community***

1. [Supplementary item 1: Data cleaning process](#S1_datacleaning)
2. [Supplementary item 2: Additional information about sample](#S2_additionalinfo)
3. [Supplementary item 3: Demographic information about Phase 1 participants, and their ideas which were rated and ranked in Phase 2](#S3Phase1Info)
4. [Supplementary item 4: Trustworthiness of qualitative analysis](#S5_Trustworthiness)
5. [Supplementary item 5: Thematic tables with supporting quotations](#ThematicTables)
6. [Supplementary item 6: Community involvement](#S6_Community)

**Supplementary item 1: Data cleaning process**

The Phase 2 survey had 5066 ‘hits’, but not all of these progressed past reading the information sheet and consenting to participate. Datasets retained for inspection (n=3825) were those where consenting participants had completed enough of the survey to be useable in any analyses (>25%). Qualtrics metrics used to identify duplicate responses and bots were “Q_BallotBoxStuffing”, “Q_RecaptchaScore”, “Q_RelevantIDDuplicate”, “Q_RelevantIDDuplicateScore”, “Q_RelevantIDFraudScore”, and “Q_RelevantIDLastStartDate” – the latter four use meta data from participants including their IP addresses and approximate locations (which were deleted once checked). The survey contained numerous opportunities for free-text responses, including the two free-text questions analysed in the main text. Participants who were flagged up by Qualtrics’ measures were retained if visual inspection revealed meaningful, sensible responses to one or more of these free-text questions (for instance, recounting their experiences of seeking help for suicidality). This process resulted in exclusion of 117 duplicate datasets and 73 suspected bots (277 datasets flagged by Qualtrics were retained after visual inspection), as well as international participants (n=116)^[[1]](#footnote-1)^ – and retention of 3167 datasets from participants (2771 autistic) who had completed *at least* the first stage of the prioritisation process (approximately one third into the survey)^[[2]](#footnote-2)^.

While results of the four-stage prioritisation process are reported elsewhere,^1^ attrition between the point of beginning the four-stage process and reaching the end of the survey (reached by 2805 participants) meant that only 2813 participants (2489 autistic) reached the part of the survey relevant for the present analyses (around four fifths of the way into the survey). Our final exclusion, taken to guard against possible inattentiveness, was that of 35 participants (26 autistic) who neglected to respond to at least 6 of 7 (i.e. 85%) selectable-answer questions in the relevant section (and provided no qualitative data). As such, present analyses include 2778 participants, of whom 2463 were autistic.

Compared with the larger autistic sample who *began* the Phase 2 four-stage prioritisation process but dropped out prior to completing the section analysed in this paper (n=308 of 2771), the 2463 autistic participants described herein tended towards greater degrees of lifetime suicidality (χ2 [4] = 10.08, p = .039). Unfortunately, nothing more is known about the 308 autistic or 81 non-autistic participants who dropped out between starting the prioritisation process and completing the relevant part of the study.

**Supplementary item 2: Additional information about key variables and the sample**

Here, we provide additional information about our measurement of key variables and the sample analysed in this paper.

**Sex and gender**

We assessed sex and gender via three questions. First, participants were asked “What sex were you assigned at birth?” and could respond with ‘Male’, ‘Female’, ‘Intersex or other’. They were subsequently asked “Is your gender identity the same as your sex assigned at birth?” and could respond ‘Yes’ or ‘No, my gender identity is different to my sex assigned at birth’. Those who selected the latter could choose multiple terms to describe their gender or enter their own response.

For descriptive clarity in the ‘Gender’ variable of the main text (Table 1), we used both questions to categorise autistic participants and non-autistic supporters/allies as cisgender men, cisgender women, or as transgender, gender-divergent or gender-questioning. Within the latter group, further breakdowns are provided for transgender men, transgender women, people with non-binary identities, and people currently unsure of their gender. On participant feedback that self-identified gender should precede sex, gender is presented first in the Table. Notably, in the qualitative data, we use participants’ own preferred terms rather than the simplifying categorisation we employed here.

**Ethnicity**

We asked participants the question “Which ethnic groups do you identify with?”. Among those participants who were not white were those who identified as Black (0.2% of the whole autistic group, and 0.3% of the autistic qualitative group; 0.3% of the whole non-autistic sample, and 0.8% of the non-autistic qualitative sample); Asian (0.9% of the whole autistic sample, and 1.3% of the autistic qualitative sample; 1% of the whole non-autistic sample, and 1.5% of the non-autistic qualitative sample); Arab (0.08% of the whole autistic sample, and 0.2% of the autistic qualitative sample; 0% of either non-autistic sample); Hispanic or Latino (0.2% of the whole autistic sample, and 0.4% of the autistic qualitative sample; 0% of either non-autistic sample). The remaining non-white participants expressed diverse multi-ethnic backgrounds (8.3% of the whole autistic group, 9% of the autistic qualitative group; 3.1% of the whole non-autistic sample, 1.5% of the non-autistic qualitative sample).

Notably, with a single question assessing identification with different ethnic groups, the survey did not enquire about race and ethnicity as distinct constructs.

**Self-reported autistic status**

At the start of the survey, participants were asked to confirm whether they were autistic (‘self-diagnosed, seeking assessment, or already formally diagnosed’). A later question asked autistic participants, more specifically, to which of these three categories they belonged.

The ‘possibly autistic’ group comprised those who were awaiting assessment and those who self-diagnosed. Individuals awaiting autism assessment comprised 65.7% of the ‘possibly autistic’ sample (65.5% of the possibly autistic group in the qualitative sample); the remainder of the ‘possibly autistic’ group were individuals who self-diagnosed (a.k.a. self-identified) as autistic.

**Differences between participants who did and did not provide qualitative data**

Of the 2463 autistic participants included in this paper, 989 responded to the opportunity to provide qualitative data (the others only responding to the closed-ended questions).

To understand possible differences between participants who did and did not provide qualitative data, we performed a series of comparative analyses of variance (ANOVA) to compare groups for age, age at diagnosis (autistic group only), recency of suicidal thoughts and attempts; and a series of chi-squared tests to examine differential distribution across different degrees of suicidal experiences, different gender groups, and the likelihood of being formally-diagnosed or possibly autistic (autistic only). For these we used IBM SPSS Statistics software (version 29).

Compared to the full autistic sample (n=2463), the subgroup who provided qualitative data were more likely to be older, *F*(1) = 50.38, *p <* .001, including at age of diagnosis (F [1] = 22.96, p < .001), and with less recent experience with suicidal thoughts (F [1] = 8.13, p = .004) and attempts (F [1] = 12.99, p < .001), though they did not differ in relation to overall experience with suicidality (χ^2^ [2] = 8.50, p = .075), their distribution across gender groups (χ^2^ [2] = 1.64, p = .441), or their likelihood of being formally diagnosed or possibly autistic (χ^2^ [2] = 2.69, p = .260).

Of 315 non-autistic participants included in this paper, 130 responded to the opportunity to provide qualitative data. The non-autistic qualitative group were likewise equally distributed across gender groups (χ^2^ [2] = 2.23, p = .327), though typically older than those who did not provide qualitative data (F [1] = 4.38, p = .037).

**Supplementary item 3: Demographic information about Phase 1 participants, and their ideas which were rated and ranked in Phase 2**

These participants are *not* those whose data is analysed herein. However, they are the Phase 1 participants who generated the ideas that were rated and ranked in Phase 2, whose choices therefore shaped the study for the Phase 2 participants analysed in this paper.

Their data is shown in Supplementary Table 3A. Unfortunately, limited data was collected about the demographic information of supporters/allies themselves. Note the table includes a small number (125 autistic and 13 supporters/allies) who chose to rank the pre-entered ideas without entering their own, but whose views therefore also influenced the design of Phase 2.

**Supplementary Table 3A**

| **Phase 1** | |
| --- | --- |
| **Autistic (n=1191)** | **Supporters/allies (n=213)** |
| *Experience of suicidal thoughts/behaviour*  % Never suicidal: 1.9  % Passing thoughts of suicide: 9.2  % Suicide ideation: 23.1  % Suicide plans: 29  % Attempted suicide at least  once: 36.8  *Average age* (SD, range): 36.6 (14.8, 16-89)  *Gender identity*  % Cisgender man: 23.7 % Cisgender woman: 52.9  % Trans, gender-divergent or gender-questioning: 23.7  *Ethnicity*  % White: 89.7 % Black: .2  % Mixed or multiethnic: 6.1 % Asian: 1.3  % Other or undisclosed: 2.7  *Highest educational attainment*  % GCSE/high school diploma /equivalent: 23.7  % AS/A Levels/Access to HE/equivalent: 14.3  % Diploma/certificate HE/degree: 36.1  % Postgraduate qualifications: 24.1  % Rather not say / missing: 1.7 / .08  *Employment/occupation**  % Any kind of employment: 49.2 % Studying: 18.5  % Engaged in voluntary work or internship: 2.1  % A carer, or raising children: 4.7 % Unemployed/unable to work: 25.5  % Retired: 4.5 % Rather not say / missing: 1.7 / .08  *Autistic status*  % Formally diagnosed: 64.2  % Possibly autistic: 35.8  % Self-identifying as autistic: 35.9  % Awaiting assessment: 64.1 | *Relationship to supportee and their relationship to suicide*  % Supporting someone without known experience of suicidal  thoughts/behaviours: 15.5  % As their parent/guardian: 66.7 % As another relative: 21.1  % As their partner or friend: 9.1 % In another kind of relationship: 3  % Supporting someone known or suspected to have experienced suicidal thoughts/behaviours: 75.1  % As their parent/guardian: 77.5 % As another relative: 5  % As their partner or friend: 13.1 % As their friend: 2.5  % In another kind of relationship: 4.4  % Bereaved by the suicide of autistic supportee: 9.4  % As their parent/guardian: 55 % As another relative: 40  % As their partner or friend: 5 |
|  | *Demographic information about supportee*  *Age of autistic supportee* (SD, range): 24.2 (11.72, 9-92)  % Aged 12 or under: 9.9 % Aged 13 to 18: 31.4  % Aged 19 to 25: 23.6 % Aged 26 to 40: 26.7  % aged 41 and above: 8.4  *Bereaved supporters/allies: age of autistic person at their death* (SD, range): 28.9 (16.39, 15-67)  *Gender identity of supportee:*  % Cisgender male: 54.5 % Cisgender female: 32.4  % Trans, gender-divergent or gender-questioning: 9.3  % Not sure how they identify / identified: 3.8  *Could they have participated themselves?*  % No, due to learning disability*:  11.3  % No, due to another disability*:  5.2 |

*Note*. Percentages reflect percentage of group as reflected in second row. Categories marked with asterisks were not mutually exclusive.

The ideas identified in their data, and included in Phase 2 to be rated and ranked by participants, are displayed in **Supplementary Table 3B**. For accessibility, these were grouped under the headings in the first column; participants were assured that the headings were used only to present ideas in a more manageable way and that they did not need to pick an idea from every heading. The order in which these groups of ideas were presented was randomised, so that no particular group of ideas was prioritised. Ideas are numbered herein for clarity, but were not when participants saw them.

***Supplementary Table 3B: Final list of ideas presented for prioritisation in Phase 2***

| **Heading** | **Ideas** |
| --- | --- |
| *Ideas for when autistic people are in crisis* | 1. A phoneline for autistic people in crisis  2. A text-based (SMS or online chat) service for autistic people in crisis  3. A phoneline for anyone supporting autistic people (such as family, carers or friends) in crisis  4. A text-based (SMS or online-chat) service for anyone supporting autistic people (such as family, carers or friends) in crisis  5. Quicker access to NHS services for autistic people in crisis  6. Safe spaces for autistic people to visit and/or stay when they are in crisis  7. An app without live chat for autistic people in crisis or to prevent crisis (to record information, signposting, keep notes or a diary) |
| *Ideas for identifying and diagnosing autism* | 8. Shorter waiting times for autism assessment/diagnosis  9. NHS autism assessment and diagnosis for all ages  10. Check for autism when children and teenagers are struggling in school  11. Check for autism when a student seeks support in higher education  12. Check for autism when people ask for or receive mental health services  13.  Check for autism when people are arrested  14.  Check for autism when people are struggling with major life events - such as becoming a parent, menopause, or suffering bereavement  15.  Support for people waiting for autism assessment/diagnosis  16.  Tell all parents about the signs of autism and what they can do if they see them  17.  Improve assessment/diagnosis of autistic girls and women  18. Post- diagnostic support when people are diagnosed as autistic |
| *Ideas to improve the NHS* | 19. Mental health treatment, therapy, and support designed for autistic people  20.  Mental health support for autistic people earlier and when they need it, to help prevent them reaching crisis  21.  NHS mental health services just for autistic people  22.  Autistic people trained and paid to support other autistic people with mental health, suicidal thoughts, and when in crisis  23.  NHS communicating in an autism-friendly way - such as being more direct and clear about what to expect and when, reasons for rejecting referrals, or what to do next  24.  Better communication between NHS services so that autistic people get joined-up support  25.  Provide a care coordinator or case worker for autistic people, to support, advocate, and help them when passed between services  26.  Find and test ways to ensure autistic people do not ‘fall through the cracks’ in the NHS  27.  Ways to use NHS services without speaking out loud (online forms or text messages) |
| *Ideas for education* | 28.  More support for autistic and suspected autistic children in mainstream schools, for autism-related difficulties such as friendships, sensory issues, feelings, and academic work  29.  Reduce bullying in schools  30.  Make mainstream education more flexible for autistic children (such as adjusted hours or curriculum)  31.  Allow autistic children and students to temporarily leave education for their mental health  32.  More support for autistic and suspected autistic students in higher education, such as college and university |
| *Ideas for improving autism awareness and acceptance* | 33.  A campaign to improve understanding and acceptance of autistic people and the difficulties they face  34.  Famous autistic people sharing their stories to help more people understand and accept autistic people (such as their experiences of mental health difficulties)  35.  A campaign to reduce stigma about mental health problems and suicide, which also tells people where they can get support  36.  A campaign to make employers and businesses more autism-friendly  37.  Teach children more about neurodiversity at school  38.  Help more people understand that autistic people sometimes need time alone |
| *Ideas for social care* | 39.  Make applying for benefits more autism-friendly  40.  Make the forms for applying for benefits more autism-friendly  41.  More financial support for autistic people  42.  Support to help autistic people understand and apply for benefits  43.  A service to help autistic adults with day-to-day living (such as managing bills, coping with housework, cooking and other everyday tasks that people find difficult, and understanding social expectations from other people)  44.  More activities for autistic people in their communities (such as accessible sport sessions, clubs, and sensory rooms)  45.  A coaching service to help autistic adults have a fulfilled and meaningful life  46.  More support for autistic people leaving education, to help them find the right next step  47.  Allow autistic people to take a break from work when they need it for their mental health (could be hours, days or weeks)  48.  Funding to help autistic people get further education or training in subjects that interest them  49.  Opportunities for autistic people to work or volunteer in areas related to their hobbies, passions or interests |
| *Ideas for peer support and self-care* | 50.  Groups or clubs that autistic people would enjoy, to help with making and keeping friends  51.  Peer support groups for autistic adults (where autistic adults support each other)  52.  Peer support groups for autistic children (where children support each other with some adult help)  53.  An autistic ‘buddy’ or ‘mentor’ for autistic people  54.  A website to help autistic people better understand their autism, look after themselves, and find support  55.  A website co-designed by autistic people to help autistic people better understand their autism, look after themselves, and find support  56.  Groups or classes for autistic adults focused on self-advocacy and mental health  57.  Groups or classes for autistic children focused on self-advocacy and mental health |
| *Ideas for training* | 58.  Train staff and volunteers in non-NHS crisis services (such as Samaritans, Shout, Papyrus) to understand and work with autistic people (e.g. the basics of autism, communication, increased risk of mental health difficulties and suicide, trauma and burnout)  59.  Train teachers and other education staff to recognise the signs of autism and know what to do next  60.  Train staff in mental health services to recognise the signs of undiagnosed autism and what to do next  61.  Train GPs and their staff to understand and work with autistic people (e.g. basics of autism, communication, increased risk of mental health difficulties and suicide, trauma and burnout)  62.  Train staff in NHS mental health services to understand and work with autistic people (e.g. basics of autism, communication, increased risk of mental health difficulties and suicide, trauma and burnout)  63.  Train everyone working in education to recognise mental health problems, trauma, and suicidal thoughts in autistic people |

**Supplementary item 4: Trustworthiness of qualitative analysis**

We followed a number of steps to ensure trustworthiness of the data, as recommended by qualitative researchers.^2,3^ These were as follows:

1. **Ensuring transparency in methods**

While summarising the nature of these questions for brevity in the main text, here we provide the wording of the questions as participants saw them.

- Qualitative question 1: “*If you would like to explain your choices in the ranking exercise, you can do so here. This is optional – you do not need to write anything if you would rather not. Because our team is limited in how much capacity we have to process the data from this survey, we are afraid that we have limited the characters to 200.”*
- Qualitative question 2: *“Is there anything else you wanted to tell us? We would like to give you the chance to have your say about our survey. Feel welcome to share your thoughts here. You do not need to write anything – but this is your chance to briefly mention anything you would like to. Because our team is limited in how much capacity we have to process the data from this survey, we are afraid that we have limited the characters to 200.”*

We also provide the [Phase 2 survey](https://livebournemouthac-my.sharepoint.com/personal/rmoseley_bournemouth_ac_uk/Documents/Files%20from%20Dropbox/ACE/Phase2_PDF.pdf) in full, so readers can view these questions in the context of their surrounding items. The first of these questions was preceded by the ranking exercise, where participants were exposed to a range of ideas varying in concreteness and indicated to universal nature; it was, moreover, immediately preceded by questions about co-design and co-production, and provision of services and support to people awaiting autism assessment, people who self-diagnose as autistic, and people who think they might be autistic, and so subsequently some participants responded in relation to these questions despite the question stating that it was about “your choices in the ranking exercise”. The second question, as the penultimate item in the survey, may have been received by participants who were already fatigued by the cognitively demanding ranking task, and this may have influenced who provided a response. Additional reflections on the influence of the survey design on participant responses are presented in the ‘Limitations’ section of the main text.

1. **Ensuring transparency in researcher positionality**

The qualitative analysis was undertaken by author RLM and ratified by author SJM, who are embedded in a team which includes neurotypical and neurodivergent (including autistic) people. Lived experience within the team includes experience of supporting someone through suicidal thoughts/behaviour, and/or bereavement by suicide.

- *Author RLM’s reflexive considerations:* In relating to the participants and their data, author RLM is an “insider”^4^ in terms of being autistic, having lived experience of late diagnosis, of suicidal thoughts and behaviours, and of difficulties accessing some of the UK services referred to in this survey. While RLM is open about her autistic status and has shared this in the context of her previous research on this topic, the extent that participants knew this is unclear: participants were informed the team comprised neurotypical and neurodivergent members with different forms of lived experience (as above), but nothing more specific about individual team members. As a white, cisgender, highly educated and full-time professional in academia, the author recognises that she has class and social privilege relative to some participants within this (albeit highly educated, majority female) autistic sample. Moreover, her experiences and views are likely to differ from those of the ‘average’ autistic person within the UK, given that the 70% of autistic people within the UK are unemployed,^5^ and many do not attend or complete higher education.

As an academic in Psychology, RLM’s professional relationship with the topic of suicidal thoughts and behaviours concerns quantitative and qualitative investigations of intraindividual risk factors, such as non-suicidal self-injury, and major theoretical approaches to suicidality; and engagement with policy-makers and professionals on this and related topics, such as difficulties in education and employment, barriers to diagnosis, and mental illness. About 6-8 months prior to writing this paper, she analysed two relevant pieces of data from Phase 1: one related to challenges seeking NHS help for suicidal thoughts and feelings,^6^ and the other related to the factors underpinning suicidal thoughts and feelings,^7^ which indicated that broad societal factors (such as lack of health and social care, poverty) were essential factors in suicidal thoughts and related theory-derived constructs, ‘entrapment’ and ‘thwarted belongingness’. These professional experiences are relevant to her perspectives on suicide prevention in that she has observed the interrelatedness of these issues in relation to suicidal thoughts and behaviour in autistic people, and has previously experienced some frustration at siloed thinking around these topics. She was moreover highly influenced, during the preparation of this and the accompanying paper^1^, by public health approaches to suicide.^8-10^ At the same time, her lack of direct experience delivering or experimentally validating or investigating indicated approaches to suicide prevention, and her personal experiences of trying to use UK services, meant that she is inclined to emphasise addressing bigger-picture systemic issues, while recognising the essential need for crisis interventions for those who need them right now.

The ideas in the Phase 2 survey, which were prioritised by our participants and influenced their responses to the subsequent qualitative (and quantitative) questions, were selected by RLM and authors SJM, TC, and TP. To avoid undue influence of any one author on the ideas progressed to Phase 2, selection was on the basis of frequency in the Phase 1 data. These four authors triangulated their interpretations of the data, maintained rigorous records of their procedure and provided thick description for the same in the accompanying paper.^1^ These were means through which RLM tried to reduce their influence on the contents of the Phase 2 survey. To document her influence and relationship to the Phase 2 data reported in the present paper, RLM kept paper notes (unfortunately unreproducible here) during the process of coding, while she was initially identifying and refining themes and subthemes. She noticed her own emotional reactions to some of the stories and emotions expressed by participants (e.g. hopelessness), and noticed that many participants appeared to share difficult experiences with services and to endorse large-scale systemic approaches to suicide prevention. To some extent, RLM felt that systemic, wide-lens views of suicide prevention were likely given that autistic people in the UK experience inequalities in numerous domains including education, employment, health and social care,^11^ and hence are likely to feel these injustices as interrelated, as she did herself.

- *Author SJM’s reflexive considerations:* Author SJM is a white cisgender female and is also an “insider researcher”, with lived experience of late autism diagnosis and associated mental health challenges. As an academic with previous experience conducting qualitative research on the topic of autism, self-harm and suicidal thoughts,^12^ she is familiar, like RLM, with literature and theory in this area; her previous experience allowed her to engage critically with these sensitive topics (as we do in this paper) and challenge dominant thinking around, for instance, the nature of self-harm. Author SJM was previously involved in the aforementioned qualitative analysis of Phase 1 data about the factors underpinning suicidal thoughts and feelings,^7^ which appeared to indicate the need to address broad societal factors underpinning suicidal thoughts and feelings.
- *Positionality of the team:* Individuals within the team draw on academic backgrounds in Psychology, Medicine, Anthropology and Sociology, and Population Health Sciences – and hence have a wide-scoping view of suicide prevention. Several have contributed to autism and/or autism and suicide prevention policy in their countries. Notably, author SC led the 2021 priority-setting exercise referred to in the text, exploring issues of greatest importance to the community concerning autism and suicide. Authors DH, SC and JR have developed, investigated and/or published on indicated interventions for aiding autistic people at crisis point. The team also includes individuals with clinical experience of working with autistic people in suicidal crises and/or severe psychological distress (TAP, IH, SBC). This, and the lived experiences of team members in supporting autistic people through suicidal thoughts and behaviour and/or bereavement by suicide, in addition to their own experiences with services, mean that they are familiar with the challenging landscape of care within the UK. The team broadly endorse a public health approach to suicide prevention in autistic people, while championing the development and evaluation of effective means of helping those at crisis point.

1. **Code-recode approach**

After author RLM had completed her initial analyses and written a first draft of the Results section, she took a break from the data for 5 weeks. On returning, she recoded a subset of the data (300 quotations, almost 20% of the entire qualitative dataset): instances where the second coding differed from the first were rare. To calculate intra-rater reliability, RLM marked each instance of divergence between the first and second round of coding with a 1, while instances of sameness were marked with 0. Averaging across these ‘scores’ led to estimated intra-rater reliability of 0.03, indicating that RLM’s first and second interpretations of this data were highly similar.

1. **Stepwise replication of qualitative analyses**

Author SJM independently familiarised herself with the data, coded it, made notes on the themes that they interpreted in the data, and only at that point examined RLM’s codes and suggested themes and subthemes. She independently confirmed the presence of themes such as “suicidality and poor mental health begins in childhood/school” (realised in final subtheme “the seed of all autistic suicide death is in childhood or early adulthood”), and pointed out aspects of intersectionality not explicitly mentioned in descriptions, such as co-occurring ADHD, while recognising an influence of her recent reading about ADHD (RLM went back through the data, on receiving this feedback, to confirm references to ADHD).

1. **Investigator triangulation and peer debriefing/examination**

Prior to these authors reading the paper, RLM discussed her interpretations of themes and subthemes with authors MP, EW, DH and SC, none of whom had seen the data, and who were relatively distant to specific details about the design of the study and analytic approach for this paper. Author RLM also presented findings informally to colleagues in Psychology working in clinical (qualitative) research. These were all ways of ensuring the trustworthiness of her process and her interpretations in the data, which were consistent with observations that authors MP, EW, DH and SC had made in independent autistic populations.

In addition to investigator triangulation, a degree of methodological triangulation of the findings lends credibility of the findings reported in the paper. The aforementioned analysis of Phase 1 data,^6,7^ while following similar methods (online survey) and conducted by members of this team (RLM, SJM, TP), corroborated experiences such as those reported by the Phase 2 sample herein: instances where, for instance, services were inaccessible and antagonistic. The findings are also triangulated to an extent by independent observations by other researchers, using different methods, in independent autistic and non-autistic samples: for instance, observations by multidisciplinary professionals, gathered through semi-structured interview, which corroborated the inflexible and inefficient nature of UK mental health services^13^; interviews with parents of who corroborated the severe impact of inaccessible school environments, lack of educational support and bullying on their autistic children^14^, and focus groups with autistic young people who confirmed the same experiences.^15^ Unfortunately, there are multitudinous empirical examples corroborating key themes of this work, such as ‘broken, brutalised systems’.

**Supplementary item 5: Thematic tables with supporting quotations**

Full thematic tables for

1. [views about the direction of suicide prevention efforts;](#Thematictable_staging)

b) [views about provision of support](#Thematictable_undiagnosed);

c) [views about co-production](#Thematictable_coproduction)

**Full thematic table for views about the direction of suicide prevention efforts**

Quick navigation:

**Theme 1:** [The “urgent” vs the “root causes”](#Thematictable_staging_T1); *Subthemes* [“The seed of all autistic suicide death is in childhood and early adulthood”](#Thematictable_staging_T1s1), [“Crisis 1st, preventing crisis 2nd, quality meaningful satisfying life 3rd”](#Thematictable_staging_T1s2)

**Theme 2:** ["Brutalised, broken systems" – the logic of suicide and the need for “radical social change”](#Thematictable_staging_T2); *Subthemes* [Acceptance, belonging and community](#Thematictable_staging_T2s1); [Accessible, appropriate and trustworthy healthcare and crisis services](#Thematictable_staging_T2s2); [Access to diagnosis and post-diagnostic care](#Thematictable_staging_T2s3); [Suitable educational provision and transition support](#Thematictable_staging_T2s4); [Suitable employment and social care in the community](#Thematictable_staging_T2s5); [Financial security](#Thematictable_staging_T2s6); [Safety from victimisation and equity within the criminal justice system](#Thematictable_staging_T2s7); and [Equality for diverse identities and needs](#Thematictable_staging_T2s8).

| **Themes,** *subthemes* | All quotations |
| --- | --- |
| 1. **The “urgent” vs. the “root causes”**   Contributed to by 8.7% of comments related to this topic. Within this theme, 14.7% of comments reflected more balanced ‘weighing’ of crisis responses vs. long-term preventative measures; these were mainly from formally-diagnosed autistic participants (52.6%), then from possibly autistic (31.6%) and non-autistic participants (15.8%). The remaining comments within this theme (85.2%) fell into the two subthemes below.  [Back to the top](#Thematictable_staging) | “Some proposals addressed root causes of mental ill health, whereas others were more 'urgent' (e.g. crisis support). I struggled to choose between most effective and most urgent”  “Difficult to prioritise between measures which are of broad benefit & will mean fewer people go far up Stress and Desperation Mountain, and those which…stop people jumping off!”  “I found the ranking exercise difficult. I tended to prioritise crisis services, while recognising that prevention (in various ways and at different life-stages) is at least as important”  “I prioritised early intervention over crisis care because I was going for ideals. However crisis care may be more important primarily until better systems are established”  “Some of these are really important for supporting autistic people who are actively suicidal, but it’s also important to achieve wider societal change to prevent people reaching that point”  “Short term the lost generation are still being let down by long waiting lists, lack of pre and post dx support. Long term, education and awareness and understanding across the population is key”  “Lack of support in childhood set me up for problems for rest of my life. Support with education and career is vital. Those of us that is too late for understand[ing?] and safe place are vital”  “I am part of the lost generation of autistic women and I suspect that what we need may be different from younger women who have not had a lifetime of trauma and masking. Different solutions needed”  “Greater understanding of the difficulties faced by young autistic adults is needed throughout society along with both crisis and timely pre-emptive support in order to bring suicide rates down.”  “I think there are two separate aspects - dealing with crisis situation we have now and preventing these rises from happening. This survey tries to do both and it is not adequate.”  “Hard to eliminate/rank as so much needs to change. Addressing NHS^[[3]](#footnote-3)^ numerous shortfalls for immediate effect. Quality of life for long term effect. Rounded approach needed.”  “I’ve prioritised suggestions which will be most likely to prevent imminent suicide based on how the questions were worded, but I think that prevention is just as important.”  “Wellbeing of children at a particular age will influence their wellbeing as adults. We need to get support right from early on, however, support for adults is just as important.”  “some of these are really important for supporting autistic people who are actively suicidal, but it’s also important to achieve wider societal change to prevent people reaching that point”  “I think support to live better lives that are meaningful is prevention, but in crisis it is also important to have support that is accessible in place. This is why I have interleaved the two.”  “I was tempted to put the app idea higher because it works for me. I use the Stay Safe app. But I think the biggest thing is to reduce the causes of suicidal thoughts. That is a lot harder though.”  “Tough exercise due to such great ideas which would all help. In an ideal world there would be so much awareness and everyday support that crisis would be less likely. As we stand crisis is a big issue”  “I placed importance on improvements in services available for acute mental health episodes, but a priority was flexibility in work/school as social attitudes are a huge barrier to accessing support”  “It was difficult to consider the impact without having a timeframe to consider it in. E.g. I think reducing bullying could have a huge impact, but probably very delayed.”  “Prevention of a crisis is better than dealing with the crisis but the crisis is more dangerous” |
| 1. *“The seed of all autistic suicide death is in childhood and early adulthood”*   Contributed to by 68.9% of comments within the parent theme. 51.2% of comments in subtheme were from diagnosed autistic participants, 39.3% from possibly autistic participants, and 9.5% from non-autistic participants.  [Back to the top](#Thematictable_staging) | “Although I believe the peak age for suicide is the 30s, the greatest waste of life and the seed of all autistic suicide death, is in childhood and early adulthood. Hence my emphasising schools & unis”  “I feel school is where it all starts. there needs to be better support for autistic people in schools, all staff should have mandatory training and support needs to be so much better than it is now”  “I have worked in the NHS for over 25years. My work has shown that early intervention is overlooked and crisis management is prioritised. It starts in childhood, we must start from the beginning”  “It begins with bullying in schools. Interestingly it takes many years for a 'professional' to identify that you're autistic, but a child of 7 can spot you and start making your life hell from then on”  “Bullying in school first as if children hadn’t bullied and shunned him and understood autism my son would not have felt suicidal in the first place. He was a happy boy before he went to school”  “I am a broken person & acutely suffering because of not having special needs support, or intervention, especially in early childhood, teens or adulthood”  “Children and teenagers need to understand themselves and their brains. I spent my childhood feeling alone, like a freak. I wasted my life. We need support earlier for young people”  “Reduce suicide by reducing trauma. That starts in society b4 MH [mental health] services needed”  “Address the root problem: Education, bullying/school life, diagnosis - reduce the number of autistics traumatised before they reach adulthood, and ill-equipped for independent life. Financial help"  “Mental health support will fall short if an autistic's financial, material or physical needs remain unmet. We must ensure autistic's have a foundation of safety, addressing the causes of despair”  “The priority should be to helping them in their normal life. These day-to-day struggles are what accumulates to the major feeling of loss of hope & desire to continue living. And parents need this too”  “All these ideas were good. It was very difficult to choose what to prioritise. In an ideal world I would rather prioritise improving everyone's quality of life to help prevent mental illness”  “I feel like the route to stopping people getting to crisis is helping them understand themselves and their reactions/emotions better earlier in life so the bad stuff doesn't add up”  “Autistic children need skills to thrive that take them to adulthood, especially Asperger children. They are clever but not socially which leads to social isolation and suicide”  “The best way we can stop suicides is by making lives worth living - friends, lovers, community, financial security via adapting the world of work to accommodate autism”  “I think it's better to do more to prevent things getting to crisis point.”  “Presently crisis intervention is priority but prevention should be the aim. NHS support resources are nonexistent, assessment situation is even worse. Many cannot get diagnosis or mental health care”  “Focusing more on prevention. I ranked things such as more training in various settings as higher because if that foundation is in place hopefully there would be less people in crisis to begin with”  “I think preventative things and squandering loneliness are the most important to lessening it, ATKEAST from my own experiences and causes.”  “I dont believe self-diagnosis, it must be medical. Prevention of suicidal thoughts key, hence work+life opportunities critical: secondary is rapid diagnosis: last is crisis management.”  “Start early, train teaching staff, ALL nhs staff & police to recognise autism in children (& adults until it’s normal) INC WOMEN so everyone has access to help asap & suicide goes down in ASD patients”  “ I put things that lead to crisis first. Autistic people should be involved in decisions.”  “The scars from childhood determine outcomes in adulthood. Deal with education and bullying first. It will significantly improve outcomes.”  “ To get mental health support at the moment, a crisis has to be reached: by then, it is too late. Why do we assume autistic people can’t have mental health issues in our system?”  “It was very hard to make a decision because all options felt important but the lack of mental health support catered to autistic support needs is pivotal in preventing suicide and crisis.”  “Educating people who work in schools is important because early issues could be resolved reducing trauma. Financial and life skill support is also important because burnout could cause more suicides.  “There are links between being autistic and trauma often from an early age. This is huge gap. There are guidelines for sensory friendly and autistic provision already but these aren't followed.  “Early Id of autism is essential in so that coping mechanisms and aids can be learnt and practised from a young age so they are more habitual, which will naturally be better engrained in adulthood.”  “I can't access healthcare and there is no appropriate mental health care for me. I've been denied benefits. Social care doesn't offer anything useful. Early support would mean fewer traumatised adults"  “early identification, diagnosis and support could help prevent autistic children from depression and suicidal ideation later in life.”  “i think the most important thing we can do is to help autistic people BEFORE they reach any kind of crisis point - preventative measures that support us to never reach this point matter greatly”  "stats will look better if diagnosis doesn't occur. care will be greatest when diagnosis does occur. Focus should be on care not stats. Principal of prevention rather than 'cure' should be implemented."  “It is crucial that early intervention is possible in order to identify and potentially de-escalate any issues as soon as possible after they are present.”  “Wider reaching and quicker diagnosis is paramount. Early in life, crucial. Prevent child hood issues of alienation and bullying and stop them spilling into and affecting adult life. Financial support”  “Early diagnosis & support is important to help prevent a MH [mental health] crisis.”  “Education, awareness and support, when young to understand how to cope with differences by those , who may be autistiç: other people to understand, that some do think differently, but still 'normal'.”  “Dealing with these issues at school age will future proof support of those people who grow up to be autistic adults.”  “A lot of teenagers make suicide attempts so help needs to be there at school age. Too many medical professionals have an outdated idea of what autism is and need retraining.”  “I think that improving the environment is most important. It's also really important to identify people who are undiagnosed and provide appropriate mental healthcare for all autistic people."  “At the moment, the NHS is actively harmful to autistic people looking for mental health support. Early intervention and genuine support needs to happen.”  The biggest factor for depression in autistic people is rejection. So reducing bullying in schools is the most essential as it may prevent autistic people needing therapy in the first place.  "I think starting with supporting children would help them to need less support (emotionally) as adults. Many professionals don’t understand autism in females. Waiting times are too long for adults.”  "I was constantly bullied throughout education, it has left mental issues that I am still struggling with today. For me, suicide is more worrying over time as I keep losing hope I'll ever feel better.”  “Early diagnosis and support is key, to improve self-esteem, coping, and friendships early in life”  “My rationale is that trauma begins in how autistic people experience the world and are stigmatised and mistreated from a young age. Then they need financial support throughout life.”  “Ranked to highlight the need to support at all stages, pre and post diagnosis and to help people BEFORE crisis. Text based crisis support v Important for those of us who may feel non verbal in crisis.”  “i think the correct mundane support when not in crisis is the key to preventing crisis."  "The waiting time for diagnosis is much much too long.”  “Early intervention is needed - Camhs^[[4]](#footnote-4)^ are overwhelmed and schools are allowed only one ED [Educational] Psych visit per term. Please help change this"  “I think spotting autism in children and getting them diagnosed early is key to preventing significant mental health issues as an adolescent and adult.”  “Early ID [identification] is key to reducing long term trauma = mental health issues. Unadapted therapy can do harm but is needed for trauma. Work capacity is lower so help is needed. Community improves mental health.”  “Due to family history of suicide attempts, I believe in early identification/diagnosis and access to timely and suitable support across all sectors for mental health issues for suicide prevention.”  “I have first hand experience of being failed by the edudction system and the NHS. My son has been failed by education. This is where we can make the biggest difference, early on!”  “If more support was provided in schools autistic children may never reach the point of crisis. Too often a child reaches crisis point but hasn't even been assessed by camhs. Everything is too slow.”  “Mental health issues start at young age. Better support needed for adults currently but for long term reduction of suicide risk need to tackle root causes in childhood.”  “Lack of diagnosis is often due to lack of access to diagnostic services. I was not diagnosed till my 30’s & both of my attempts were when I was an undiagnosed child/YA [young adult]. Early support is vital.”  “30+ years of being undiagnosed and not knowing what the problem was did the damage to me. Educate/diagnose kids/teens early, get them the support/care early, stop them reaching crisis as an adult.”  “Start with schools, school was hell for me as an undiagnosed but obviously autistic girl. The teachers missed all the signs and the students ridiculed me. 12 years later I'm still struggling from it.”  “As someone diagnosed in adulthood, I feel early diagnosis and consistent support for students to understand themselves and succeed would have made such a difference to my life.”  “It all starts with correct childhood diagnosis and support followed by long-term support and opportunities”  “Improvements should be aimed at children then this should decrease trauma from things such as bullying and will give them the recognition, information and the tools needed to navigate life”  “I have chosen the options that I think will help people not reach crisis point. If supports are put in place to help navigate a NT [neurotypical] world, autistic people will perhaps be less likely to feel suicidal.”  “The outlines were very brief, but I tried to focus on awareness first, support second and then emergency intervention third. With more awareness and support there should be less need for intervention.”  “Prevention is better than cure. Since I realised I'm probably autistic I feel my mental health is so much better. I wish I'd been diagnosed as a child and people had supported me and understood me.”  "Prevention of suicidal ideation is better than treatment. Mental health difficulties first start in childhood. Target strategies there."  “Extra autism support is sorely needed in the 16-25 age bracket. Please help the next generation be the first not to suffer.”  “My rankings are given as a diagnosed adult without a learning disability. The best suicide prevention is crisis prevention, that is done by creating feelings of a hopeful future and social connection”  “Ranked for potentially the biggest impact at the earliest opportunity - in childhood. Many inicators of future mental health issues can be seen in childhood - own experiences. Identify/help - faster"  “Some of the options seemed to almost duplicate others. Although diagnosed at 57, and wanting more awareness for "mature" autistics, I believe suffering can be more effectively reduced by early diagno”  “How about working on their self esteem as children? I have approached many sports clubs to see if my autistic child could join and have been told t look elsewhere. There is no elsewhere for our kids.”  "Early recognition is vital to mental health. Most NHS staff even in mental health settings don't recognise it, especially in women/girls/adults in general! Support sooner could stop suicide.”  “Priory above all should be in supporting young autistics in understanding themselves and their condition”  “Damage was done for me by the end of primary school. I accepted the fact I had to stay alive because suicide was illegal and would make my parents sad. Still mostly why I'm alive.”  “I believe earlier intervention could save lives. Treating autistic burnout could prevent suicide rather than being misdiagnosed as depression.”  “I think early intervention and continuous, well coordinated care and case management/ownership is critical. Access to psychiatrists who are autism aware is also critical.”  “Autism tool box ,don't wait till someone has a formal diagnosis to offer it, we waited 2 years, we may have avoided crisis if we had toolbox at the start instead of post diagnosis.”  “The survey addresses provision not looking at aggravating factors such as trauma, isolation, exclusion and misunderstanding. Building autistic agency and enabling people to follow their passion. Do it”  “All these ideas were good. It was very difficult to choose what to prioritise. In an ideal world I would rather prioritise improving everyone's quality of life to help prevent mental illness.”  "As a parent of a young person who is AuDHD and has experienced mental health crisis which resulted in self-harm and suicidal thoughts, I believe early intervention and prevention is crucial.”  "Quality of Life would have prevented Crisis.”  “Children and teenagers need to understand themselves and their brains. I spent my childhood feeling alone, like a freak. I wasted my life. We need support earlier for young people.”  "Services are in a dire state at the moment, so it does seem like some of these suggestions are unachievable but also good to see what people have considered. Changes need to start with schools."  “Early, supported services and identification for young people are key to healthy adult lives, reducing incidence of crisis in youth and adulthood.”  “Help needed as early as possible in a child’s life as this will affect the whole path we will take with regards to mental health/ suicidal tendencies.”  “I think obtaining support and recognition without judgement at an early age would have led to a happier more fulfilled life.” |
| 1. *“Crisis 1st, preventing crisis 2nd, quality meaningful satisfying life 3rd”*   Contributed to by 16.4% of comments within the parent theme. 57.9% of comments in subtheme were from diagnosed autistic participants, 36.8% from possibly autistic participants, and 5.3% from non-autistic participants.  [Back to the top](#Thematictable_staging) | “Crisis 1st. Preventing crisis 2nd. Quality Meaningful Satisfying Life 3rd”  “1. Immediate help for those most at risk. 2. Help that is designed for autistics (with possible alexithymia). 3. GP Support for all autistics. 4. Better life experience for upcoming generations”  “1 Helping person in crisis. 2 foundations to prevent crisis (breaks from education, help w benefits). Less important: clubs, self-advocacy classes, help in small way but don't address core issues”  “ As someone who has made attempts many times - the vital component is quicker access to MH care. 6-24 month wait lists are insane and causing deaths. Crisis care is also very poor/barely existent”  “ I chose the autism-friendly safe space and helpline during suicidal crisis as particularly important because being able to stay at the Edinburgh Crisis Centre has saved my life on several occasions”  “When you are suicidal, quick help is needed, hence being able to contact someone without speaking or battling a GP receptionist, who does not think your case is urgent enough if vital”  “Instant, safe text or online chat access for crisis support most important. You need to know they won’t call the police e.t.c as v dark feelings can be normal and they pass.”  “my choices in the ranking with the main objection of initial support for the autistic person,then wing people.suicide prevention priority then mental stimulation/ isolation relief. Beyond the cracks”  "texting on crisis lines is high priority. i find verbal communication very difficult in those situations and it would majorly help. + educating those on crisis lines about autism is priority"  “I think the priority has to be harm reduction and suicide prevention. If those providing services do not understand or value autistic people how can they support them?”  “Diagnosis and crisis treatment comes first for me as a diagnosis can help avert crisis and asd people are often in crisis. Next is others understanding NHS then school. Last is access to care"  “Personally I think the Samaritans training and education is most important cause I can't call them as an autistic person cause they make everything worse and I was kicked out of school.”  “I ranked based on ability to implement. There are many small things we can do that. Quite cheap too. It is a shame not to do it. A text support service for autism sounds like a lifesaver. I would use”  “I have focused on those in crisis already. But many of the lower priority things could prevent the crisis and so some of these things would be less essential”  “Poor NHS support. Little is done to prevent autistic people from falling through the cracks and being unheard. Text lines would help autistic people who struggle to talk on phones”  “Survival 1st, stop fear(benefits).Stop the bullying,ALSO in institutions and PRESS!Not 'on list'SEEKING diagnosis support desirable.INSTANT support is a must.Peer support skilled YES 121 trained,paid!”  “Feel priority should be services autistic people can access in crisis in terms of suicide prevention but also timely diagnosis, training and support in education & workplace can make a huge different”  “When you are suicidal, quick help is needed, hence being able to contact someone without speaking our or battling a GP receptionist, who does not think your case is urgent enough if vital.”  “Personal preference as a "High Functioning" autistic, though in relation to the other end of the spectrum with the communications issues (like my grandkids) a crisis line for carers would be top.” |
| 1. **"Brutalised, broken systems" – the logic of suicide and the need for “radical social change”**   Contributed to by 91.3% of comments related to this topic. Within this theme, 14.2% of comments spoke about the ‘system’ broadly (59% of these comments were from formally diagnosed autistic participants, while 26.8% were from possibly autistic participants, and 14.2% from non-autistic participants). The remaining comments under this theme (85.8%) pertained to one or more of the subthemes listed below (comments sometimes pertinent to more than one subtheme).  [Back to the top](#Thematictable_staging) | “There are so many gaps in understanding and provision that even the ten I picked are the tip of a very large iceberg of need”  “Until we change the system, which wasn’t built for us, we’re stuck playing whack-a-mole”  “The world needs to get a lot better about not crushing the souls out of autistic folk”  “School and the NHS have done irreparable harm to me and they can never be trusted again. I hope that these brutalised, broken systems are improved but I do not foresee this happening in my lifetime”  “The current system isn’t fit for purpose. My son went into crisis and was high risk of suicide we had no help. I had to get him through it myself”  “I lost my sister, and the cracks in the systems were very noticeable”  “Adults with autism who need only support with day to day tasks are not supported in any way only by family”  “This is so relevant for my daughter at the moment. She is quite often suicidal and her life is so limited that I totally understand why. She is 17 and has no friends. She struggles to go out”  “My suicide attempt wasn't a mental health crisis. It was just I knew I couldn't be who society wanted me to be. I suspect there's a lot of suicides like my attempt, where no crisis is involved”  “I now fear for the future and how I would cope if my partner died… There is no other support available so I have an exit plan”  “Life with autism and mental health problems is lonely, bleak and meaningless. I'm 50 and don't want to live into old age. I'm very worried about life without my parents.”  “Autists tend to be pragmatic/sensible, we kill ourselves if our assessment is that the future does not look to be worth living.”  “Therapy needs to support managing existential grief and being considered disordered by society. Too much gaslighting of autistic individuals’ accurate view of human condition & traumatic experiences.”  “I work in schools... I see it every day. I hear about early intervention every day... but it doesn't happen. My kids were failed. I was failed. Suicide in autistic people is logical. Living is hard”  “People with autism do not live meaningful or fulfilling lives. This will not change. Medical assistance in dying should be made a legislative priority to alleviate the undue suffering of people.”  "I'm sorry this survey is impossible! How could anyone.choose from ALL those fictional options - with no support! I have suffered so much! All my life alone. This is not life, this is hell!"  “Suicidal ideation/intent must be recognised as a rational response to an existence of suffering &struggle”  “Every time the government says we're lying I get a bit closer to the edge. This world isn't for us. We can't be different, but they don't believe us, what's the point?”  “Why protect us from suicide when we are made actively vulnerable by NHS Policy and Government politics?”  “Suicide ‘prevention’ must go hand in hand with respecting autonomy and validating chronic suicidal ideation without resorting to carceral measures”  “Thank you. When you feel like your book has no more chapters. All you can think about is leaving this world.”  “i have tried to find out why suicide is 'wrong' without considering either religious beliefs or how if might affect you friends / relatives but have been unable to. why?”  “Hard to eliminate/rank as so much needs to change. Addressing NHS numerous shortfalls for immediate effect. Quality of life for long term effect. Rounded approach needed.”  “Honestly, we need all of these things and more. It took multiple mental health crises for me to be diagnosed autistic at 20 years old as a woman, after going through many NHS mental health services.”  “Very difficult to prioritise the options during the ranking exercise. The fact I chose 59 from the original 63 ideas is a damming indictment of how the system fails autistic people every day.”  “Limiting my choices to 10 was impossible, they are all vital”  “I wanted to end my life twice, one time because of school and one time because of work. The world is not designed for autistics, especially the ones with high sensory issues.”  “All of the original set of ideas seem desirable. I have tried to pick out those that I consider most likely to combine effectiveness and practicality.”  “I think this project is a really good initiative. Thank you so much for it. Many of these ideas would make a real life-enabling and literally life-saving difference for autistic people. Thankyou!”  “So many of those choices would help, so it was very difficult choosing.”  “Thank you so much for creating this survey. There were so many great ideas included, but do think that multiple forms of support need to be implemented to make a real change.”  “Please change something. We are the most disparate, disempowered group and least likely to be able to co-ordinate to change things ourselves. Please look at Spectrum Gaming, they give us hope”  “I am 44, a woman, lives with my dad and lost my mum in 2022. We need help, support, kindness and more than anything, hope.”  "Could not select enough that would make a big difference. With overlap, more than 10 initial options HAVE helped me avoid a near-miss.”  “There is a lot of misunderstanding and a high suicide rate - all areas need better support”  “It is important to help society to have a general understanding the Autism is not an illness, we just have a different operating system - we should not feel afraid to be who we are. Equality for all.”  “I just want to add I feel like all 10 are hugely important, putting one down as “least important” felt wrong."  “It comes down to a more understanding society. Procedures are put in place for structure, but there seems to be huge financial and effort/work cost for little to no positive results currently.”  “An Autism crisis is hell to navigate, easy to fall into and like trying to climb Everest to get out of. So much more neds to be done. We are quiet and therefore easy to ignore.”  “I appreciate this is a thing I hope the system can improve for us right now so many people are struggling because of the lack of support right now”  “Excellent initiative. It is vital we improve services and support for Autistic services.”  “We are good people and want to be included in your world, with a little support we are of value, utilise our specialness to save lives, thank you.”  “Hard to narrow down to 20 choices and then organise 10 into importance because all felt equally important in reducing or preventing autistic suicide”  “I had recurrent crisis because I don't have support, even though I am diagnosed. This invisible disability is very stereotyped and people don't recognise it as such, especially in females.”  “I tried to rank them in a way that reflected the idea that existing services need to be work better for autistic people, alongside developing more specialist support specifically for autistics.”  “Based on what I felt would be most impactful. However I feel there is little to differentiate the 1st and 10th most important factor. Therefore the 10th placed may only narrowly be less important”  “Right now so much of the system has been designed for neurotypical people that getting the right help and support impossible for many autistic people as well as the lack of support for autistic adults”  “There needs to be more financial and structural support for autistic people. Societal infrastructure, biases and attitudes are heavily to blame for the alienation and hopelessness autistics face.”  “All the ideas presented were very good and would be of great help.”  “To get mental health support at the moment, a crisis has to be reached: by then, it is too late. Why do we assume autistic people can’t have mental health issues in our system?”  “This was harder than I thought to complete, narrowing down the options as I thought they were all helpful. Thank you for this study which will give support to future autistic people and share awareness”  "Currently - there is zero help...And no opportunity to give views on anything. Autistic people are invisible.”  “This survey was a little difficult because I feel like all the ideas were very good. In an ideal world all ideas would be implemented. Thank you for doing this survey.”  “Actually I think if we just had a healthier education and work culture, and if difference was more widely understood and tolerated - or even celebrated - that would make the biggest impact.”  “So much needs to be done it was hard to limit the choices. In short - we are made to feel 'wrong' all day, everyday, and support is sparce and often damaging, we want to give up.”  “Services are severely underfunded. The waiting listctimes for diagnosis is far too long. Mainstream secondary schools are not designed for neurodiverse children and teachers lack knowledge”  “There is not enough support for autistic adults, especially those deemed to have low needs.”  “It was very hard to make a decision because all options felt important but the lack of mental health support catered to autistic support needs is pivotal in preventing suicide and crisis.”  “We need to spread more awareness of what being autistic is like & that it can present differently. All services need in depth training and should treat people flexibly even if someone is not diagnosed”  “There are links between being autistic and trauma often from an early age. This is huge gap. There are guidelines for sensory friendly and autistic provision already but these aren't followed.”  “It was extremely difficult to narrow down the answers at stage 2 as all the options would be very helpful, making deselecting very hard. Need more help”  “if the world was set up to support autistic individuals then it would work better for everybody. It is about recognising individual needs and ensuring that everybody can be their best self. “  “All these ideas were good. It was very difficult to choose what to prioritise. In an ideal world I would rather prioritise improving everyone's quality of life to help prevent mental illness.”  "I can't access healthcare and there is no appropriate mental health care for me. I've been denied benefits. Social care doesn't offer anything useful. Early support would mean fewer traumatised adults"  “This is a really difficult thing to live with without support in adulthood. I realise now that my entire life has been a struggle not because I am a ‘weirdo’ but because I have this condition.”  “Being autistic in this current society is unbelievably hard. Even with a diagnosis I still have zero support. On top of long covid and my epileptic mum, things are hard”  “Suicidality is associated with unmet needs related to autism. I tried to think about what the problems are re unmet needs & how they might be best addressed given my knowledge of the current systems.”  “Need somewhere safe when in crisis (not currently NHS). ND-affirmative treatments needed. Relieving everyday burdens of living incl engaging with services would have greatest impact on qual of life.”  “My child has had an awful time and due to diabolical lack of funding / support/ knowledge/ wait time we had to go private. I suspect I also have it but mask.”  “Understanding masking, links between autism & mental health, less able to access support & services, not being asked the right questions, unconscious bias & stereotyping, communication difficulties”  “Lack of communication & training in nhs/social work/ education is POOR excuse is lack of funds Eduction employ staff after completion of first year with little experience because their pay is less”  “My daughter would be alive had she had even 5% of the care in her plans. The focus is all on assessments /reviews but there is no delivery. Nobody believed her/us, as professionals and parents”  “I volunteer with a charity that supports families of autistic adults and we have been campaigning for all these for last 20 years but apart from consultations nothing happens! Hope this helps!!”  "It’s so important to tailor services to meet autistic people’s needs. We don’t for u see normal services. We need joined up services. It’s all so exhausting. Menopause and autism is horrific.”  “focus on undiagnosed support. Making sure people don't get forgotten by systems and revive care without self/family advocacy and understand timelines of this with regular checks and updates.”  “Services in [redacted] have always been IQ based so my son received no help when needed it. As an adult struggling and again batted about until reaches crisis. They don’t matter here. Heartbreaking.”  “Better services designed for Autists by Autists delivered in a timely manner. In a world not built for us compassion, grace and understanding give us hope for the future. No hope = risk of suicide”  “Services are in a dire state at the moment, so it does seem like some of these suggestions are unachievable but also good to see what people have considered. Changes need to start with schools."  “When you work and "cope ok" this can be dangerous. People don't understand how much work impacts us and the system doesn't allow for breaks or change very easily. Not enough understanding or support”  “At the moment autistic people mostly don't get what they need unless they have someone good at advocating for them and even then maybe not. This needs to be different.”  “I think practical in person support for people who diagnosed or awaiting one should be top priority followed by organised and streamlined care then education and raising awareness of autism”  “Autistic adults commit suicide for one of two reasons 1) They are undiagnosed, cannot cope, and don't know why. 2) They are diagnosed and there is no 'suitable' support for them to access.”  “There are no meaningful services for high functioning autistic adults.”  “I have seen many u diagnosed people with Asd reduced to a life of misery and poverty due to lack of support and understanding.”  “I've lost a lot of friends due to preventable suicide. Being autistic in modern society is so hard. It feels like things will never change to allow us to thrive - but we're the inflexible ones?”  “Sometimes it’s not suicide, the world just overwhelms them and they can only see one way to stop everything feeling so demanding. Everything is a fight for autistic people. It shouldn’t be that way”  “Thank you :) please tell the government to stop traumatising disabled people because it’s not even saving them money”  “Tough exercise due to such great ideas which would all help. In an ideal world there would be so much awareness and everyday support that crisis would be less likely. As we stand crisis is a big issue”  "From experience, I feel that education limits the problems experienced by autistic people to a learning disability, when in my family, it is primarily, an environmental disability. I see my family's autism to be an environmental disability, and the modern environment continually makes life more difficult”  “It all sounds lovely but it seems hopeless that any of this would come to reality. It feels useless and a burden to have autism in this climate. Nobody wants to deal with me”  “The violence enacted on disabled people by false scarcity kills. If you're trying to reduce deaths stop creating false scarcity, it's inhumane”  “Very few of the autistic people I now work with get through education without trauma. There is very little support for autistic adults without learning disabilities.”  “I don’t even have the words to advocate for myself. I have never been supported with autism. There is no support and it’s not surprising we want to die. Society hates us. It hates disabled people.”  “Falling through the cracks and not getting autism appropriate support in a timely manner has greatly increased both mine and my peers' suicide risks so sorting this is of the utmost importance”  “My eventual serious suicide attempt was preventable. I am an intelligent, problem solving person. I needed support from those who understood and would HEAR what support I needed to manage this society”  “The main issues are receiving diagnosis, so that life starts to make sense, and support, so it feels like there's a positive way forward.”  “Once you are diagnosed officially, there is no support and then you self explore help but then try to understand mask but the world won't allow you too. It feels like a massive internal war everyday.”  “The world is very autism unfriendly .the lack of understanding is awful”  “Once a diagnosis is received, esp as an adult you just have to get on with it. Just keep working, keep surviving. No support or understanding about how difficult work and life are for us”  “There's no support for autistic carers. Nobody considers that the carer could be autistic too and every request for help is met with "he's autistic, you have to do it for him". I'm autistic too.”  “1 training and awareness in health/social care, education & judiciary 2 Well designed autism friendly services & programmes 3 Accessibility & communication differences across all services”  “I feel like the options were repetitive and fed into each other so that was hard to rank. The main issues are the wait times, the lack of any support especially for adults, and the benefit criteria.”  “Access to services is hard the way things are currently set up for example Managing phoning for a phone call GP appointments is very stressful and there are no alternatives.”  “Bullying is massive, work/school/in life. Support is scarce and for visible disabilities.mate crime"  “The sooner there is total neuroaffermative system change and understanding the better. Don’t forget ADHD.”  "The waiting time for diagnosis is much much too long. Early intervention is needed - Camhs are overwhelmed and schools are allowed only one ED Psych visit per term. Please help change this"  “the system is broken, and autistic people are burning out more than ever. what is supposed to support us is now hell to navigate- every time we need help it's a fight. why is accessing help torture?”  “I personally would have chosen all options available. However I prioritised based on both lived experience and practicality (to an extent).”  “I have selected options that I think empower and inform autistic people. Also I selected for both formal support services, and opportunities for social support and finding their tribe.”  "They are all very important. I have experienced no support whatsoever throughout my life and I’m a 48 year old woman. I’m also profoundly deaf. I cannot express the impact this has had on my health."  “People in general want to succeed in living. Autistic people are no different. Dignity,respect, belonging and self advocacy matter. Meaningful support towards this is key. Not all families are helpful”  “All autistic need advocacy of their choice . Mentoring. Access to living options without group living , sensory, counselling and daily living, financial support .subs open 24hours villages built.”  “Its so important that support is accessible, that there are options other than death, and that there are more opportunities for undiagnosed autism to be picked up so people can see there might be hope”  "All students at school should be assessed for autism, (AQ Test) even the ones 'without problems'. It's not rocket science. Then actually provide services that can be accessed at all stages of life.”  "Due to family history of suicide attempts, I believe in early identification/diagnosis and access to timely and suitable support across all sectors for mental health issues for suicide prevention.”  “The existing system isnt working, mostly because autistic people are ignored. I only got a diagnosis because i had to stay at an inpatient home (unrelated) for monitoring and they saw unmasked me”  “Capitalism must end - we cannot thrive under the weight of the regime. It will kill us all - autiistic or not.”  “To prevent suicide you need to change attitudes in society so we don't face discrimination and bullying. You need to train staff to understand us. You need to give us ways to cope and support to cope.”  “I think the 5 Ways to Wellbeing are a good guide - services that help autistic people maintain those 5 things, targeted at life events where they get disrupted, like becoming a parent/redundancy etc”  “Having accessed "autistic services" it is often clear that these exist just as a box ticking exercise and are not in any way useful or practical and only act as to further isolate”  “Ranking does not take into account current laws that protect neurodiverse individuals including education & health. The NHS & Local Authorties, NHS ICB^[[5]](#footnote-5)^ & social care behave unlawfully. Fix that!”  “Fulfillment and meaning are so important to autistic people, and if we feel fulfilled and that our lives are meaningful then it will make a huge difference in regulating mental health.”  “There are so little services that cost nothing and it's hard when you can't work but need help. I believe you should be formally diagnosed to get help or it gets overwhelmed”  “It all starts with correct childhood diagnosis and support followed by long-term support and opportunities”  “I made my choices because I am a late diagmosed female and there is nothing in the way of support and it has been very very hard. If it wasn't for my college being helpful I would have nothing.”  “Ranking was hard because some feed into others, and some may negate others. Eg better awareness and intervention would increase diagnosis but diagnosis will only help if support is available.”  "My son was diagnosed at 28 and previously had a severe mental health condition diagnosis. We have fought for support for the last 10 years and the system has failed us. No coordination of services"  “Experience is that people employed to run these services are one dimensional, and incapable/unwilling to tailor support based on need. Autistics with high level intelligence are ignored.”  “Utterly impossible to narrow down to 20 then less so this survey doesn't truly reflect what I think because they need everything listed within this survey.”  “Being autistic I avoid going places I don't know the etiquette, I find it hard to be in a community or have people understand why. You just need to bridge the gaps for life to be livable for us.”  “I was misdiagnosed and in and out of therapy for 15 years before being diagnosed. There’s no support out there that caters to autistic individuals, more generic support for mental health etc”  “My choices were ranked in this way through painful years of fighting and struggle to get him the right help and support when I knew very little! I’m now aware at 62 I am also ADHD and Autistic!”  “I have ranked preventing bullying lowly. It is too vague. I think educating adults with authority and influence - parents, teachers, police, doctors and health and social workers should be a priority.”  “Children and adults from poor backgrounds (maybe due to multi-generational autism in the family) are far less likely to be diagnosed and supported by the aporaphobic society we live in.”  “There are no services and support for people awaiting diagnosis or afterwards. People, including professionals still don't understand autism and that needs to change so that support is available.”  “I was diagnosed at 23 and I believe the services available when growing up would still caused mental harm based on my older brothers experience. Diagnosis does not mean understanding either.”  “I feel it’s basically impossible for an autistic person to survive society working full time if you even get a job . It’s extremely overwhelming. I include full time students / children in this.”  “I have found support entirely lacking in terms of what I can get help for as a formally diagnosed autistic person. Because I “appear” capable and verbal, I am expected to live without support.”  “I am currently in crisis and support is sparse, disconnected and nit fit for purpose. There is more useful support on social media and through ND peers. Luckily I am an intelligent, mature person.”  “It's very difficult to rank because all of them are so important. But without access to diagnosis, appropriate support and the financial means to make choices then everything else is irrelevant.”  “The last 2 rounds reducing to 20 and 10 were impeccably hard- all of the options are SO essential.”  “an understanding to begin with, people with autism being able to follow what they are interested in and/ good at as well as the understanding of parents and professionals, then risk of suicide reduce”  “I work full time and find it very difficult to access support and services for my autism because they only run when I’m working (9-5 weekdays, nothing in evenings or on weekends) I feel isolated.”  “The most important factor, as with other parts of life, is empower us to shape our own destiny rather than choose it for us.”  “Please drive change - autistic people need support and protection”  “I have little faith that suggesting more funding will fly with the government. Autistic people need more support into work and more access. Young people need early support about society’s structure.”  “Thank you. I really feel for myself individually the biggest reason for my suicidal ideation is a lack of belonging and contributing to society basically disabled with daily tasks but waiting to die”  “Autistic ppl will not thrive if they are forced through the same work/school meat grinder as everyone else. What helps us helps the whole of society.”  “Much needed survey, to close the gap in the infrastructure. In 2025 the world is still not autistic friendly”  “Every turn is difficult when trying to get a diagnosis and support. As a parent, you have to fight for everything and you shouldn't have to. I was told to keep my child away from anything sharp”  “Many of your suggestions are based on people getting diagnosed, but this won’t change anything”  “I’m so glad someone is finally doing this research. Due to personal experiences, I know how difficult and hopeless it feels to live in a world not built for you and ending is the only way out. Thanks”  “Most autistic people i know are traumatised. Treat the trauma/ptsd and alot of the mental health symptoms become less severe”  “I would struggle to get my son to fill this in. He won’t access services due to negative previous experiences . He rarely leaves the house and will never access a group”  “I’m convinced all NDers are trauma victims. Identifying this over autism alone would be more effective”  “In my experience, all services have been dire”  “There is a disconnect between my academics & my social ability to navigate the adult world. I feel inadequate because my paper qualifications suggest I should be flying and yet I constantly struggle.”  “As a parent and in my professional life (NHS, Social Services, and Education) I have found recognition, understanding, and skills of autism lacking and too long await for diagnostic assessments”  “Autistic people are amongst the most marginalised in society. Neither myself or husband are diagnosed but we have 3 Autistic YP. At ages 18 ( twins ) & 25 all are NEET.”  “Daughter struggled thru schl & at university-little support.Educating everyone on how to treat ASD people,schl,GP,mental health staff wld make diff.”  “My daughter and my experience of the NHS, LA [local authorities], Education and work has resulted in both of us experiencing trauma and feelings of hopelessness due to lack of understanding of being autistic by others”  “I am really struggling with my job and feeling intensely burnt out. My mental health is very poor. I don’t feel my autism is acknowledged in my daily life or the ways it makes me struggle”  “Great survey, resonated A LOT. Well done. People need so much more help than they are getting, females are particularly neglected. Huge progress to be made.”  “Please do all that you can to ensure autistic people are seen in society. Our experience has been that there is absolutely no support or mental health services.”  “It's hard being autistic in a world that doesn't cater for us and give us the freedom to live life the way that works for us. It leads to burnout, meltdowns, physical illness”  “All the ideas put forward are really great and well thought out so it was difficult to choose.”  “Well trained mentors and advocates for all aspects of life are required.”  “I feel that adults with Autism who need only support with day to day tasks are not supported in any way only by family”  “Diagnosis for me was in a time autism in girls was a new concept. But my biggest struggle has always been the office politics neurotypical people can't seem to live without. This culture harms us.”  “Stop being so negative and start to realise everyone has a contribution to make but if you make us deficient, chronic, disability - nothing will every change.”  “The Leadbeater bill to introduce assisted suicide devalues and dehumanises autistic people and many will agree to commit suicide due to pressure from society and lack of support. It must be stopped.”  “Thank you for researching suicide and autism: it's such important work as I and many other autistics face regular mental health crises due to lack of money, employment, housing and social contact.”  “Everything possible to level up the life chances and mental health of suicidal people must be done, it the right thing to do as a society.”  “I struggle to cope with relationships and the wider world. There needs to be more tailored support for Autistic people”  “Autism needs to be identified so the right help can be put in place, but there's no point identifying the autism if there's no autism specific help available.”  “The most important thing is having support available that's designed for autistics, as well as staff having knowledge about what autism looks like. Nobody should be dismissed.”  “In my experience those who have power such as NHS, schools, and workplace mangement have inadequate understanding and just view any training as a tick box exercise.” |
| 1. *Acceptance, belonging and community*   Reflected in 14.7% of comments within the parent theme (59% of which were from formally diagnosed autistic participants, 30.5% from possibly autistic participants, and 10.5% from non-autistic participants).  [Back to the top](#Thematictable_staging) | “I believe isolation and loneliness to be the greatest hardships suffered by my son who has autism”  “My choice was primarily based on my personal experience. finding a peer group was the most profound support for my suicidal ideation and losing that peer Group was the most profound knock back”  "Isolation and feeling overwhelmed predominant - complexity and unpredictability stressful. Need for trusted individual(s) - friends, professionals - and processes. Support needed for friends."  "Reasons for autistic people to have suicidal thoughts are isolation and feeling different/a bother.”  “I have selected options that I think empower and inform autistic people. Also I selected for both formal support services, and opportunities for social support and finding their tribe.”  I think that the NHS, education, police etc need to be trained on individual differences. They shouldn't need to know someone is autistic in order to treat them humanely.”  “Lack of friends who can relate to you or you can be yourself around, leads to chronic loneliness and isolation. Professionals often fail to see how great your support needs are, because you ‘look ok’.”  “I have ranked preventing bullying lowly. It is too vague. I think educating adults with authority and influence - parents, teachers, police, doctors and health and social workers should be a priority.”  “How about working on their self esteem as children? I have approached many sports clubs to see if my autistic child could join and have been told t look elsewhere. There is no elsewhere for our kids.”  “an understanding to begin with, people with autism being able to follow what they are interested in and/ good at as well as the understanding of parents and professionals, then risk of suicide reduce”  “I work full time and find it very difficult to access support and services for my autism because they only run when I’m working (9-5 weekdays, nothing in evenings or on weekends) I feel isolated.”  “Thank you. I really feel for myself individually the biggest reason for my suicidal ideation is a lack of belonging and contributing to society basically disabled with daily tasks but waiting to die”  “Thank you for considering us. Society has rejected and laughed at us for so long and there's been little help, none for us women and girls who were ignored and doubted and still not been diagnosed”  “Early ID is key to reducing long term trauma = mental health issues. Unadapted therapy can do harm but is needed for trauma. Work capacity is lower so help is needed. Community improves mental health.”  “People need to understand that a lot of ‚support‘ for autistic people is still about making them neurotypical and this is part of the problem.”  "I think it's vital to have groups based on interests available for me.Not general support groups if there are general support groups they need to be very regimented for me. I can't cope otherwise"  “The main issue is isolation, and not having a non verbal way to ask for help (including for those ho do speak as they can't express some things)”  “I think we sorely lack community but the most pressing issue if that the NHS refuses to treat autistic people with mental illnesses. It’s not surprise the suicide rate is so high.”  “Feeling misunderstood & isolated is very common for autistic people and can lead to depression then thoughts of suicide, feeling like nobody can help you so the only way out is suicide. A very sad”  “I think suicide in autistic women and girls is likely related to being bullied and ostracised while not knowing they are autistic. They begin to believe they deserve it and that they are the problem.”  "Personally 'groups' would not be of help. Biggest help would be educating others into acceptance. Teachers don't identify girls who study hard and keep to rules as struggling"  “Pls focus on diagnosing women & girls appropriately, de-stigmatisation of ASD in all spheres.”  “Isolation is the hardest thing. Also as high masking female, finding other girls like me is difficult. I have male friends both autistic and not and they’re all gross and annoying. Give me some girls!”  “Diagnosis for me was in a time autism in girls was a new concept. But my biggest struggle has always been the office politics neurotypical people can't seem to live without. This culture harms us.”  “I placed importance on improvements in services available for acute mental health episodes, but a priority was flexibility in work/school as social attitudes are a huge barrier to accessing support”  “Many kill themselves after losing their support network and financial support. They struggle to work or pay rent. The fear is overwhelming. Most live with others and don't work. They need money.”  “People need to put more effort into understanding autistic people rather than punishing us or trying to make us think and do things in ways neurotypical people do. It harms us and leads to problems”  “We need to work on the stigma. More education, more famous people plastered across the media with the message that it’s ok to be autistic, it’s just a different way of thinking.”  “For my son, the biggest struggle he has is making connections with people but he doesn’t want to join autistic groups - he wants to be accepted in the mainstream world.”  “Many autistic struggle with change so adolesence is hard ans is transition from education to work. Autistic people need to feel as though they belong and are valued in society too.”  “I think the most important thing is to improve the ability for autistic people to participate in society and use their skills to benefit their community while still allowing for rest.”  “You need to make it clear neurodivergent people are born and come from 1 or 2 neurodivergent parents. There needs to be an understanding that many of us are differently abled not disabled.”  “Autistic people particularly when highly intelligent struggle with expressing their feelings, social interactions and finding and worthwhile work/employment. This leads to feelings of worthlessness.”  “I think obtaining support and recognition without judgement at an early age would have led to a happier more fulfilled life.”  “Thank you for doing this research - I have been suicidal whilst waiting for my autism assessment because there have been next to no peer support groups where I can talk to others about my problems.”  “My brother died aged 25 in 1991 jumped off clifton suspension bridge. No one suspected Autism, never picked up. He was very bright but " didn't fit in this world, never designed to be this way"  “Signs of ASD were obvious when I was a child, but I remained undiagnosed till 57. This was the start of severe life long MH issues. The stigma is terrible, even when seeking help 4 suicidality”  “I think preventative things and squandering loneliness are the most important to lessening it, ATKEAST from my own experiences and causes.”  “We, as was my son are seen via a neurotypical lens. We are not seen as different, rather broken and made to conform to be accepted. Prevented from being who we authentically are. This drives suicide”  “I am 44, a woman, lives with my dad and lost my mum in 2022. We need help, support, kindness and more than anything, hope.”  “The biggest damage is the lack of diagnosis, the long wait to get one and people's ignorance to what it is like to survive with it.”  “Society needs to be more flexible in their expectations of what people should do. Managing everything is overwhelming and pressuring us to do so, ends up killing us.”  “Peer-diagnosis given the same legal status as medical. More opposition to harmful messaging (jigsaw pieces, curing/ preventing autism, etc.)”  “People often say they accept autistic people until it comes to a trait that isn’t just being quiet or very interested in something. E.g coming off as rude / not understanding social cues”  “The survey addresses provision not looking at aggravating factors such as trauma, isolation, exclusion and misunderstanding. Building autistic agency and enabling people to follow their passion. Do it”  “I feel identification and support/resources for autistic people to understand themselves will be the best way to counter high suicide rates. Raising awareness of nuerodiversity generally willalsohelp”  “Therapy needs to support managing existential grief and being considered disordered by society. Too much gaslighting of autistic individuals’ accurate view of human condition & traumatic experiences.”  “Misplacement is unaddressed. "Supported living" is first used to unburden functional relatives. To prop up an autist among non-autists with only food and shelter in helpless bewilderment is torment.”  “We need to spread more awareness of what being autistic is like & that it can present differently. All services need in depth training and should treat people flexibly even if someone is not diagnosed”  “It is important to help society to have a general understanding the Autism is not an illness, we just have a different operating system - we should not feel afraid to be who we are. Equality for all.”  “I considered suicide to stop the overwhelm while trying to finish writing a book. Simply having an autistic person say, “Yeah, I know. It’s a lot. But you’ll be finished soon”, would have helped.”  “I work in domiciliary care. I got bullied by a patient. I was told by manager to go in other room and cry union said the employer didn't have do anything. I have asked for autistic mentor not coping.”  “Isolation and sense of rejection are bad for health. Knowing there are groups available for engaging socially and understanding mindset are very useful. Physical Exercise should be on the list too.”  “Any times i've had suicidal thoughts its been due to negative attitudes of those around me to my autism which often cause far greater lasting mental harm than most of the symptoms of my autism.”  “The power and experience of autistic community is extremely impotant in combatting lonliness, so any autistic-led/peer-support group is essential.”  “i’ve been suicidal as long as i can remember. struggle to make friends, feel like an alien, sure i’m going to crash out and quit my first adult job i got at age 38”  “My biggest issue is lack of social groups and isolation. Being forced to socialise is patronising and worse for me. I want a group to just be comfortable around.”  "Early recognition, intervention, & support is essential. The earlier autistic people understand themselves, the better. Validation, understanding & support from others is also key."  “Greater understanding of the difficulties faced by young autistic adults is needed throughout society along with both crisis and timely pre-emptive support in order to bring suicide rates down.”  “HUGE stigma personally faced in all NHS services. Reduce harsh judgments from ignorance through education. Lived Experience is key for services development & provision.”  “I think education for all ages is vital. Maybe I wouldnt have felt so broken if I had known I was autistic”  “I had recurrent crisis because I don't have support, even though I am diagnosed. This invisible disability is very stereotyped and people don't recognise it as such, especially in females.”  “This is really important and I’m glad someone is looking into it. Unfortunately the solutions presented are not easily solved, raising awareness of autism and getting rid of stigma should be a start.”  “A mentor, like in the 12 step programe after diagnosis, as it is very isolation.”  “keep people safe in crisis, then prevent people getting to crisis. education and understanding is key, more projects like the Oliver Mcgowern training so society understands autism”  “Actually I think if we just had a healthier education and work culture, and if difference was more widely understood and tolerated - or even celebrated - that would make the biggest impact.”  “All these ideas for prevention made me annoyed, cause I realised how bad the situation is. Mostly ppl uneducated about ASD.”  “Ita so important because neurotypical people really don't understand alot of the challenges which autistic people experience.”  “Personally 'groups' would not be of help.Biggest help would be educating others into acceptance. Teachers don't identify girls who study hard and keep to rules as struggling"  “We need the empathy and understanding we can only really get from other neurodivergent people. I also need to be able to take a break when I'm struggling. Nothing is designed for us. A constant grind.”  “thank you for creating this survey. You are saving millions of autistic people (including me) from feeling misunderstood and feeling alone.”  “Educating people should be the top priority. I can't believe so many people still don't understand autism & believe high functioning people are all gifted super hackers with no problems.”  “High functioning 1960's girl, clearly 'odd' from very young but never diagnosed. It runs through our family. We do not need 'extra' help if neurotypicals are not arses to us! Educate them.”  “Information for autistic people in crisis may help them find support and realise they are not alone. Teach young people about neurodivergency will help communication between allistics & autistics.”  “Neurotypical attempts to make us 'conform', misinformation about vaccines, and stereotypes about autism all contribute to negative mental health in autistic people. These all need active tackling.”  “I am 50 and would have benefited massively from identification and/or support as a child. Support, including via peers, would help as an adult. Other people understanding autism would also help."  “Ideas that pay autistic people to mentor others is amazing! I have tried a lot of jobs but ususally get fired for being wierd (visibly disabled) but for example i could helpmentor someone through PIP”  “Understanding masking, links between autism & mental health, less able to access support & services, not being asked the right questions, unconscious bias & stereotyping, communication difficulties”  "I selected mostly for more support primarily, but broader awareness, acceptance and accomodation are needed. Adjustment ( to fit in) should not fall entirely on Autistic people"  “I think it is more important to educate neurotypicals about what autism is, how it affects people and how they can support people who may be autistic. The problem lies with the lack of understanding”  “Even with mental health support I found I was still really struggling in times of crisis. We need pportunities to help us with our hopes/goals, like making friends/relationships and fueling hobbies.”  “Autistic people trained and paid to help undiagnosed/newly diagnosed understand themselves is the key”  “The world is very autism unfriendly .the lack of understanding is awful”  “I will eventually kill myself because I have to work full time and my needs are not being met and no one cares. If I could get benefits enough to live on I might survive but I know I won't.”  "Diagnosis and crisis treatment comes first for me as a diagnosis can help avert crisis and asd people are often in crisis. Next is others understanding NHS then school. Last is access to care"  “Other autistic people could be very beneficial in helping support other people due to how well they often relate.”  "I need to know: what mental health support would work for me and how to get it. How to get independence from people who do me harm. How to make them stop and understand."  “There is a great stigma where neurotypical people often think of autistic people as being "retarded" or expect them to be Rain Man-type figures, or generally just see and treat them as weird or rude”  “Diagnosis and acceptance are the key things.”  “The ideas relating to understanding & recognition of autism come 1st as I lmany late diagnosed so did not access support so no point in support if no one knows they are autistic or can get a diagnosis”  “I think it’s sad I’ve felt different etc and everyone blamed CP”  “Zero tolerence campaign re impoliteness major problem as autistic people often seen as impolite and this is problematic. I now declare that I'm autistic to prevent staff using this to refuse to help”  “Financial instability was a big trigger for suicidal thoughts for me during university as a full-time student. I never called helplines because I didn’t want to speak. Helping us find community is key”  “I think education about rights should include how it impacts disabled people because it is often forgotten about”  “To reduce suicide ideation, autistics need to feel much less alienated. We need help to allow us to function more fully as part of society. Isolation is the hardest thing. Also as high masking female, finding other girls like me is difficult. I have male friends both autistic and not and they’re all gross and annoying. Give me some girls!”  “I feel like Neurodiverse people seem to naturally gravitate towards each other. As I have gotten older (42) I realise that the small amount of connections I have left are all suspected or diagnosed. Pls focus on diagnosing women & girls appropriately, de-stigmatisation of ASD in all spheres.”  “Wider reaching and quicker diagnosis is paramount. Early in life, crucial. Prevent child hood issues of alienation and bullying and stop them spilling into and affecting adult life. Financial support”  “Most suicidial ideation in autism is likely from social exclusion and continual negative social experiences, it was in my case.”  “The lack of awareness and understanding of autism is and how it manifests has caused me the most problems and even led to life threatening situations. This is what causes me the most distress.”  “I believe Education re autism ( and neurodiversity in general) is important for everyone now more info is now available. We're catching up slowly.”  “Autistic children need skills to thrive that take them to adulthood, especially Asperger children. They are clever but not socially which leads to social isolation and suicide”  “Better services designed for Autists by Autists delivered in a timely manner. In a world not built for us compassion, grace and understanding give us hope for the future. No hope = risk of suicide”  “The hate that people have towards people like me come from people from ALL walks of life and itis the only prejudice in the modern age that is still socially acceptable.”  “I am not listened to, just ignored or made to feel stupid. I get sad sometimes.”  “The best way we can stop suicides is by making lives worth living - friends, lovers, community, financial security via adapting the world of work to accommodate autism.”  “Would suggest that maybe options in terms of education of others might be an idea to add.”  “I have an IQ of 166 mensa and clinically quantified. I am an accomplished teacher, Level 2 Autistic. Furious with the language and model of me! Unbearable. Autistics to teach and support Autistics!”  “I think practical in person support for people who diagnosed or awaiting one should be top priority followed by organised and streamlined care then education and raising awareness of autism Autistic people need to be loved and accepted for who they are, when they become adults there is nothing to help develop friendships, support at work, my son works full time so needs weekend help”  “Neurotypical (NT) people ALWAYS try to paraphrase what we say, we mean the literal things we say. NT people in NHS M-H services without neurodiversity training can make things dangerously worse.”  “I think that some of the work, such as supporting the general public and education for children in school, and explaining need for time alone overlap considerably so one project could cover all.”  “I struggle with depression and suicidal ideation because with autism and chronic illnesses I find it impossible to keep on top of and do daily activities.m without support of others.”  “Education, awareness and support, when young to understand how to cope with differences by those , who may be autistiç: other people to understand, that some do think differently, but still 'normal'.”  “I have seen many u diagnosed people with Asd reduced to a life of misery and poverty due to lack of support and understanding.”  “My suicide attempt wasn't a mental health crisis. It was just I knew I couldn't be who society wanted me to be. I suspect there's a lot of suicides like my attempt, where no crisis is involved.”  “I am aware of the power of peer support. I believe autistic people should be involved at every stage including design and running services. This is genuine co-production not tokenistic involvement.”  “Bullying and the ignorance of others has a huge impact on mental health. Knowing why you see/do things differently really helps, for yourself and others, formal diagnosis or not.”  “No option here for physical contact. Sometimes all I need is a hug to feel better.”  “From personal experience, I have had years of therapy which wasn't always helpful but support and education are crucial"  "Early identification and support is key. My experience is that a lot of the suicidal ideology stems from trouble communicating and being understood. Also, stop letting allistic people speak for us."  "Autistic involvement is key ....insight! Support from another autistic person.... people feel they are not alone. Help with executive function....so overwhelming. Time off at crisis points."  “The biggest factor for depression in autistic people is rejection. So reducing bullying in schools is the most essential as it may prevent autistic people needing therapy in the first place. “  “In my personal experience, my suicidal tendencies have mostly been because of stigma in education, like now, I feel like I'm never going to succeed in college because I don't do well in mainstream”  “Helping people understand” isn’t useful or often possible. People will understand or they won’t, education won’t always help. Autistic awareness is a buzzword at this point.”  “We can help each other as we know what it feels like when we are feeling so bad and want to help others not feel that way”  “Being/feeling, battling constantly alone, being treated as a fake or just constantly misunderstood, not having any access to a life we need/ being treated like it's something we want is not a life”  "Reasons for autistic people to have suicidal thoughts are isolation and feeling different/a bother. Bullying is massive, work/school/in life. Support is scarce and for visible disabilities.mate crime"  “Neurologically typical people can only do what they observe. Without the input of autistic lived experience services though well meant fail to really help. We need society to understand and accept us.”  “More research is needed on medical impacts of autism and less on social normalisation, which encourages masking and mental health issues. These seem for NT benefit, not ours - we need inclusion.”  “People in general want to succeed in living. Autistic people are no different. Dignity,respect, belonging and self advocacy matter. Meaningful support towards this is key. Not all families are helpful”  “Neurotypicals have a very broad understanding of autism.”  “All autistic need advocacy of their choice . Mentoring. Access to living options without group living , sensory, counselling and daily living, financial support .subs open 24hours villages built.”  “I am AuDHD so it is very difficult but I think work support is the most important with peer mentor support.”  "Education can change everything and help understanding aswell as awareness. Education can then enable suitable wnd actionable decisions such as implementing training, services etc"  “A well trained GP and support network beyond within the mental health care system and education is vital to the wellbeing of a person of any age being correctly assessed then assisted to function.”  "Autistic people (professionals) supporting other autistic people = both speak “the same language”. Very important. NHS needs to be accessed via non-verbal communication! Can’t accessSupportOtherwise"  “Autistics in crisis needs to be understood - Employing autistics to counsel others and train NHS staff is needed - good communication and a safe autism friendly space is imperative during crisis”  “Key factors: enable people to be able to afford to live, and then get support from peers rather than have those around them make it worse ie bullying, and also reduce systemic sexism.”  “I feel misunderstood, unaccepted and unwanted by society. By educating society and raising awareness, combined with legal protection, autistic people will find acceptance and accept themselves.”  “It is very important that autistic people are accepted and are able to actually live and participate in society therefore financial inclusion and access is very important.”  “To prevent suicide you need to change attitudes in society so we don't face discrimination and bullying. You need to train staff to understand us. You need to give us ways to cope and support to cope.”  “Finding it slightly confusing but what do know is I’ve always felt different and struggled with socialising etc, people say it’s bad to have a “label “ but isn’t it just as bad to feel alone etc”  “Having autism can be very lonely and I assume is the main reason suicide rates are high. Building connection and a community is key for support.”  "There are more pieces to my puzzle: I'm high-functioning. It's just enough to ruin your life, but not enough to look disabled to others. I know I can sink. Biggest problems are money and stability."  “Survival 1st, stop fear(benefits).Stop the bullying,ALSO in institutions and PRESS!Not 'on list'SEEKING diagnosis support desirable.INSTANT support is a must.Peer support skilled YES 121 trained,paid!”  “My rankings are given as a diagnosed adult without a learning disability. The best suicide prevention is crisis prevention, that is done by creating feelings of a hopeful future and social connection”  “Being autistic I avoid going places I don't know the etiquette, I find it hard to be in a community or have people understand why. You just need to bridge the gaps for life to be livable for us.”  “Bullying in school prevents autistic ppl from evolving any social skills. It also drags into workplaces where we can't select who we have to work with. Also, make public spades more autism friendly”  “The most challenging thing is doing the stuff everyone else finds 'easy' or 'normal'. Assumptions like 'everyone knows that' can cause mental health deterioration.”  “I prioritised increasing understanding of non-autistic people. Their lack of understanding can be the most depressing thing of all.”  “There are no services and support for people awaiting diagnosis or afterwards. People, including professionals still don't understand autism and that needs to change so that support is available.”  “I was diagnosed at 23 and I believe the services available when growing up would still caused mental harm based on my older brothers experience. Diagnosis does not mean understanding either.”  “The biggest barriers I have found has been the complete lack of post diagnostic support but also the mind-blowing ignorance of many neurotypicals when it comes to autism. We need more education of NTs”  “I would really benefit from a peer support group to feel less lonely and more comfortable in myself as a person.”  “It is important to educate people on the benefits of neurodiversity and STOP being so negative. Educate and neurodiversity is an evolution - we are only different in a neurotypical world.”  “It takes far too long to get an assessment. The general public believe that autism (along with its difficulties ‘might get better’ and is poor mental health, of course it is a life long condition.” |
| 1. *Accessible, appropriate and trustworthy healthcare and crisis services*   Reflected in 23% of comments within the parent theme (60.4% of which were from formally diagnosed autistic participants, 24% from possibly autistic participants, and 15.6% from non-autistic participants).  [Back to the top](#Thematictable_staging) | “It is appalling to me that I can be sectioned for being autistic under the MHact [Mental Health Act], but the clinicians responsible have no idea how to help in a crisis. They make it worse, make you feel no one can help”  “A phone line is only helpful if it actually helps, endless signposting contributes to suicide. Current ‘help’ is fractured, unco-ordinated smoke and mirrors, none of it is autism or carer friendly”  “A lot of teenagers make suicide attempts so help needs to be there at school age. Too many medical professionals have an outdated idea of what autism is and need retraining.”  “I think that improving the environment is most important. It's also really important to identify people who are undiagnosed and provide appropriate mental healthcare for all autistic people."  “At the moment, the NHS is actively harmful to autistic people looking for mental health support. Early intervention and genuine support needs to happen.”  “I think the person with autism needs more support with mental health, especially at times of crisis. This support needs to be regular and often by professionals trained in autism.”  “It's incredibly difficult to be heard by professionals who don't understand, when your disability is based in communication issues within your own body, never mind others.”  "Training in education /nhs is poor the excuse is no funding available there is little communication with other groups/ agencies involved. There is little support when young people exit education”  “Thanks so much for doing this. The major issue right now is zero mental health support tailored to autistic people. The nhs hasn’t the time, resources or understanding. It’s a definite crisis.”  “I actually think Autism experts within existing services that are integrated into teams but have access to Autism friendly spaces may be most useful, due to the number of co-occuring conditions.”  “Access to services is hard the way things are currently set up for example Managing phoning for a phone call GP appointments is very stressful and there are no alternatives.”  “Lack of friends who can relate to you or you can be yourself around, leads to chronic loneliness and isolation. Professionals often fail to see how great your support needs are, because you ‘look ok’.”  “We need specialist trauma services for autistic women who have been raped or sexually assaulted. We are more likely to suffer this than our peers but there is no specialist support on the NHS”  “provide autistic people with specific (not generalised) support. Separate Mental Health Teams from Autism Support. They are two different things."  We need to stop misdiagnosing Autistic/ADHD people with a personality disorder because they are self-harming or suicidal. Specialist Pathway for ADHD/autistic people with emot/relation difficulties.”  “The most important thing for me is mental health services designed directly for autistic teens and adults. And options under the NHS that aren't just CBT- which, anecdotally, doesn't help a lot of us.”  “Research into mental health therapies and treatments. CBT and SSRIs often don't work on us. There needs to be bespoke treatment.”  “Autism villages built. Autism specific social care. Autism sensory rooms nhs"  “Neurotypical (NT) people ALWAYS try to paraphrase what we say, we mean the literal things we say. NT people in NHS M-H services without neurodiversity training can make things dangerously worse.”  “therapies such as cbt or behavioural activation can make autistic burnout worse, not better. We desperately need affordable mental health services that work for autistic people before we reach crisis”  “Thank you, this is so important. It's so easy to despair when the 'normal' help doesn't work. It's so important to be understood and supported properly”  “There are too many steps to access NHS or financial support. Because of my autism this is overwhelming. I have no access to autism-specific support. I feel this is a main factor in my unemployment”  “I hope that more tailored support will be provided by the NHS for late diagnosed adults with autism and mental health conditions.”  “When I was struggling most with my mental health, I found there to be next to no options for help that were catered to autistic people- local therapists and CBT programmes would not be very effective”  “We need a Specialist Pathway for Neurodivergent People with emotional/relational difficulties. They should not be given PD diagnoses which overshadow the neurodivergence. Autism not PD. ADHD not PD.”  “Early identification can really help. Too many mental health interventions are designed for NT people and can be actively harmful in Autism and in any event, don't address the root cause of distress.”  “Mental health support designed for neurodivergents is of vital importance. 'Adapted' therapies (CBT) are not working, most professionals are not trained to work in a neuro affirmative manner either.”  “We need a specialist NHS service for autism. My local area has one for elderly, people with chronic illnesses, learning disability, etc but none for autism Also care leavers with autism need help”  “You are missing something critical. There needs to be more research into therapies both pharmaceutical and talking therapies that are better suited to the needs of autistic people.”  “Services often do not feel like they are designed with neurodiverse people in mind, especially primary mental health services.”  “When I rank highly the idea that autistic people should have prompt mental health support when they need it I imagine it as being helpful, appropriate mental health support, which it is not at present”  “Early ID is key to reducing long term trauma = mental health issues. Unadapted therapy can do harm but is needed for trauma. Work capacity is lower so help is needed. Community improves mental health.”  "Extremely let down by nhs mental health services. They need to do better. They need to have more knowledge. Services need to be adapted so autistic individuals can access services”  “Access to mental health support that actually works for an autistic brain and is easy to access is the most important thing. NHS staff that don’t get it can make things worse.”  "CAMHS is not set up for autistic children- the system and staff fail completely. Teachers and SLT do not understand autism in girls and cannot cope/comprehend with the fall out of undiagnosed autism."  “Girls and women are diagnosed too late - poor MH. Longer wait times leads to deterioration in MH. Support & therapies NEED TO BE ADAPTED. Focus on strengths and interests rather than deficits helps.”  "I have had types of therapy which seemed to be aimed at people who think/are different to me. Being told “feelings aren’t facts” in therapy isn’t helpful when people genuinely are judgmental of me."  “There is no specific help for ASD kids with dangerous behaviour they have lengthy wait for hugely over stretched CAMHS. Autism is a disability so they should have a separate specialist service to help”  “I was misdiagnosed and in and out of therapy for 15 years before being diagnosed. There’s no support out there that caters to autistic individuals, more generic support for mental health etc”  “Things like CBT commonly used for depression don't work for my neurotype. Its silly trying to trick a brain that analyses everything and is resistant to change into believing something new.”  “Validation of autism is crucial for some people to know and accept themselves. Education and health professionals must be better informed and proactive.”  “I don't think an NHS online form is enough. For 10 years I happily booked online appointments with my GP. 6months ago this system was removed and must now go through an online triage system. I am bloc”  “I feel there needs to be much more support from the NHS and those in education for people with Autism diagnosed or undiagnosed. Bullying should be taken more seriously.”  “Missed the most important option that I am desperately searching for. Online, ongoing email support. So many phone lines out there and I can't access them. Samaritans email is my lifeline but limited”  “In crisis I get bounced between different services mainly because the main NHS mental health teams do not have the understanding or training to help autistic people, also everything relies on PHONE”  "Autistic people (professionals) supporting other autistic people = both speak “the same language”. Very important. NHS needs to be accessed via non-verbal communication! Can’t accessSupportOtherwise"  “Poor NHS support. Little is done to prevent autistic people from falling through the cracks and being unheard. Text lines would help autistic people who struggle to talk on phones”  "There are alot of autistic people, with or without diagnosis that aren't able to verbalise on a telephone. The main support for people in crisis involves using a telephone."  “When you are suicidal, quick help is needed, hence being able to contact someone without speaking our or battling a GP receptionist, who does not think your case is urgent enough if vital.”  "I can't see that group activities will help autistic people. However, the app is very good as written communication is often easier than verbal. Autistic people find phone conversations exhausting."  “I think we sorely lack community but the most pressing issue if that the NHS refuses to treat autistic people with mental illnesses. It’s not surprise the suicide rate is so high.”  “Mainly NHS related choices because my experience with NHS MH services has been terrible. Staff often don't know what it actually is and dismiss autistic girls as anxiety. NHS adult autism service good”  “It was hard to rank & reduce, there are so many good ideas. G.P. awareness has to improve especially with old thinking amongst male G.P.s where women are concerned. Better neurodiversity training.”  “The medical profession, employers and education system need to update their understanding of autism and BELIEVE and not gawomen and girls when they are struggling with ND-rooted mental health issues”  “It's very difficult to choose, I'm a late diagnosed woman who was told I can't be autistic because I am articulate. We need medical professionals to understand autism in girls and women.”  “Repeated experienced lack of knowledge and training with regards to ASD in NHS mental Health Services towards an adult woman.”  “Personally I think the Samaritans training and education is most important cause I can't call them as an autistic person cause they make everything worse and I was kicked out of school.”  “92% of autistic women live with PMDD, PMDD causes severe depression in the luteal phase, in a lot of cases this involves suicidal thoughts. There is a lack of knowledge on PMDD at work and nhs”  "The assessment for asd needs to gear up for girls. It's to much geared for boys. And the metal health's services are diabolical, I believe hubs in the community and schools should be the way forward"  “I think it is brilliant you are doing this. Things need to change especially for females where so much is blamed on hormones and GPs aren’t aware of autism signs.”  “The NHS needs to do a lot better in recognising autism, especially in women and especially when we're distressed. The dismissal I faced should not happen, nor should routine misdiagnosis with BPD.”  “There is a severe lack of understanding people need to be more aware from a very young age. Gp and parents, also teachers.”  "Thank you for a clear and accessible survey. In my view better awareness of undiagnosed signs amongst teachers and mental health services is the key and benefits everyone (neurodivergent or not)."  “Generally, I think that appropriate training is the biggest issue for those who work in education, the working environment and health services – to be sensitive to recognising someone who may be autis”  “I really hope this helps improve access to support when required but perhaps if NHS and education were more aware of danger of suicide, they’d help more rather than sending them to a website!”  “I grew up with 0 support both before and after my diagnosis. My struggles are invisible to healthcare professionals and I don’t get the life skills support I need. Education traumatised me very badly.”  “Missing: non-mainstream school options, freedom of dress in school + work, certainty of no compulsory treating when go to mental health, opportunities to tell researchrs + policymkrs past experiences”  "Diagnosis and crisis treatment comes first for me as a diagnosis can help avert crisis and asd people are often in crisis. Next is others understanding NHS then school. Last is access to care"  “Healthcare and education are so important.”  “I have first hand experience of being failed by the edudction system and the NHS. My son has been failed by education. This is where we can make the biggest difference, early on!”  “A well trained GP and support network beyond within the mental health care system and education is vital to the wellbeing of a person of any age being correctly assessed then assisted to function.”  “Currently post autism support doesn’t exist for young adults. Just for family. NICE says MH supports must have someone trained to give MH support when that isn’t always the case. Schools don’t help”  “I placed importance on improvements in services available for acute mental health episodes, but a priority was flexibility in work/school as social attitudes are a huge barrier to accessing support”  “Children are let down by schools, CAMHS, where they could easily be referred for diagnosis. Then let down by mental health services as an adult, which includes GP, Psychiatrists, counsellors, CMHT.”  “Instant, safe text or online chat access for crisis support most important. You need to know they won’t call the police e.t.c as v dark feelings can be normal and they pass.”  “I had ptsd, anorexia and suicidal idiology triggered in me by bullying from my GP and an eating disorder psychologist. They directly caused me to feel suicidal. I’m not the only one.”  “I still experience regularly NHS mental Health staff who do not know about or understand ASD.”  “The paradigm used by new services cannot be the same dehumanising paradigm the medical community has used so far. And thank you for your work on these issues.”  "ND people NEED to be working with ND people. As an autistic woman, I have received better medical care from NHS women with autism.”  “There was so much bullying at school. All kids needs to be sensitised to the needs of others, including all educational staff who allowed bullying to take place. Most doctors and therapists the same”  “Majority of NHS staff not trained to deal with autistic people. Very little support available after diagnosis. Employers fail to address the simplest of issues, which could improve work and life.”  “Thank you for the opportunity to be involved with service improvement. I hope practitioner awareness, access to support and diagnosis will improve soon.”  “I was refused mental health support by NHS because I asked for RAs and then became suicidal.”  “My teenage daughter is autistic and CAMHS NHS service (Wirral) refused to help her severe mental health crisis age 12-14 BECAUSE she has Autism! Rapid NHS help would have really helped vs refusal.”  “diagnosed at 62 years (female ) life of carnage, on Valium at 11 if only I knew I could have had a power of attorney in adult life. . 2 divorces 3 sons later. let down by GP’s”  "texting on crisis lines is high priority. i find verbal communication very difficult in those situations and it would majorly help. + educating those on crisis lines about autism is priority"  “Glad someone is trying to help. People, the NHS or doctors in my experience have been condescending and ignorant. We are told to ask for help when they have none to provide leading to further dispair.”  “I think the priority has to be harm reduction and suicide prevention. If those providing services do not understand or value autistic people how can they support them?”  “Our daughter is 12. We have faced difficulty accessing mental health support for children with suicidal intent. Even the hospital crisis team said they were for adults.”  “The vital part for me is that NHS needs to deliver MH support that works for and understands he ND brain.I was in the care of CAMHs and CMHT on and off for about 19 yrs before I got diagnosed at 32”  “Samaritans are crap, sorry. I really think additional recommendations for support/crisis should be added to your list. Samaritans are just a listening service which isn't necessarily of any use.”  “GP's be careful of literal thinking, being told I cannot access support because not harming myself, led me to harm myself. MH professional should consider asd especially if past therapy has not worked”  “I have attempted suicide over 50 times in the past year and still have no mental health support. The mental health team refuse to make Reasonable Adjustments and I think want me dead”  “Samaritans do not know how to help autistic people specifically so when we reach crisis we don't bother calling because we know they're not likely to understand fully”  “There needs to be services that can be accessed in crises that understand autistic people as present differently and hard to express emotions. Equally training important to prevent getting to crises”  “I rang NHS mental health crisis line last June. My friend tried to speak on my behalf as my autism makes speaking on the phone difficult, esp when I'm in distress. But the advisor was rude to us both”  “Start early, train teaching staff, ALL nhs staff & police to recognise autism in children (& adults until it’s normal) INC WOMEN so everyone has access to help asap & suicide goes down in ASD patients”  “Iq of over 130, missed diagnosis till almost 30. Been told by GPS I wasn't asd, on and off SSRI's, turns out I am. The only reason I didn't stage exit left, was people that I cared for. Keep this up!”  “One issue is that older women do not have the opportunity to be assessed. Long waiting lists, the issues with assessing women, but key is we get turned away when we ask about assessment by GPs.”  “HUGE stigma personally faced in all NHS services. Reduce harsh judgments from ignorance through education. Lived Experience is key for services development & provision.”  "1. Immediate help for those most at risk. 2. Help that is designed for autistics (with possible alexithymia). 3. GP Support for all autistics. 4. Better life experience for upcoming generations"  “My experience of NHS ASD support post diagnosis is zero. I have seen a decline in NHS mental health support generally & recently worryingly lack of understanding by NHS staff of ASD and mental health.”  “Mental health serviceaand NHS ICB do not support asd and severe mental health. 111 and crisis team you go around in circles. Its the wild wild west which ends in death!!”  “Assessment waiting lists are so long (over 5 yrs locally), need education for GPs, s, MH to not make things critically worse, eg reacting to overwhelmed frozen people with anger, restraint and tranq”  “When someone 'gets it's and it's amazing. 99.9% of the time it's not. GPs are pointless, uneducated and lack access to suitable services. My work civil service has been better support than NHS.”  “Having experienced mental health crisis myself many times, there is huge misunderstanding in mh teams and misdiagnosis of personality disorder. Coproduction is essential”  “It's soul destroying when a doctor or nurse doesn't know the basics of autism.”  “The ranking was very difficult, but I based it upon my own life experience. I struggled a lot at school. But I put the text service higher as I have been in crisis and unable to call anyone.”  “Based on my own personal experience. None of the professionals I encountered over the years with my mental health issues mentioned autism. Had to pay privately.”  “I’m autistic and am an NHS MH nurse. Clinician’s lack of understanding of autism (beyond the stereotypes) is a huge issue that negatively impacts autistic people, which is why I ranked this first.”  “Co production and training is vital but it has to be implemented which in my experience did not happen. Awareness - only useful with action. A&E - not a “safe space” for anyone in mental health crisis”  “Need somewhere safe when in crisis (not currently NHS). ND-affirmative treatments needed. Relieving everyday burdens of living incl engaging with services would have greatest impact on qual of life.”  “I have a lot of trauma from mental health professionals not knowing how to work with or help autistic patients, and I know that is the same for a lot of people”  “People who are Neurodiversent understand what their peers go through, it's as simple as," that, they can help each other, Gp are pretty much ignorant to Neurodiverscity, this must change as we are”  “Mental health services were high on my rankings as I’ve found there to be little understanding of autism, I went undiagnosed for years. Feeling misunderstood makes things worse.”  “Services shouldn’t be co-produced by autistic people, it should be led SOLELY by autistic people. Also very explicit consent for all services and no mandatory reporting to police”  “Financial instability was a big trigger for suicidal thoughts for me during university as a full-time student. I never called helplines because I didn’t want to speak. Helping us find community is key”  “Through my responses I hope you can see that making sure a mental health crisis in an autistic person is responded to and is the start of light at the end of the tunnel is incredibly important”  “From experience there's not enough understanding from nhs professionals on neurodiversity, 40 minute appointments should not allow them to say "she's not ausistic" when masking. The waiting TO long.”  "Training in education /nhs is poor the excuse is no funding available there is little communication with other groups/ agencies involved. There is little support when young people exit education"  “As someone who has made attempts many times - the vital component is quicker access to MH care. 6-24 month wait lists are insane and causing deaths. Crisis care is also very poor/barely existent.”  “I live with suicidal ideation daily I have since I was 17 years old and I’m now 52. It’s distressing. I want faster help for problems related to mental health and a system designed for autistic people”  “AFAB dx'd autistic @ 5, heavily abused between 9-19, pushed around by CMHTs with no help for 7yrs, mis-dx'd EUPD, fucked NHS off, found help thru MHcharity, fought to get dx of complex ptsd after 8yrs”  “GPs and secondary care being better trained is most important, as they currently cause significant harm to autistic people through ignorance.”  “Falling through the cracks and not getting autism appropriate support in a timely manner has greatly increased both mine and my peers' suicide risks so sorting this is of the utmost importance”  "I think starting with supporting children would help them to need less support (emotionally) as adults. Many professionals don’t understand autism in females. Waiting times are too long for adults."  “Better knowledge in NHS teams are needed. My child was unnecessarily restrained and intimidated and needs neglected by staff in a and e and paed ward - had 18 attempts and no real support from camhs.”  “The most important thing is having support available that's designed for autistics, as well as staff having knowledge about what autism looks like. Nobody should be dismissed.”  “I was misdiagnosed with bipolar and treated with antipsychotics what harmed me permenantly. I was sent away from crisis team as my auto sim was mistaken as lack of engagement. There was no help”  "Many of my crises have worsened because medical/mental health professionals have not understood or respected my autism. I have been sectioned because doctors make assumptions rather than listening to”  “The times I've been suicidal are often late at night when I'm alone and I catastrophise in my head. Having a service where you could talk to someone who can talk you off that ledge would be helpful.”  “Being treated in the right way by anyone in the nhs is paramount. I tried to commit suicide in hospital because of lack of awareness and understanding by staff if you don't have a formal diagnosis.”  “If you want to stop people who are autistic killing themselves then there has to be someone "safe" to turn to when you're in crisis. I do not have a diagnosis.The waiting time for assessment is 3 y”  “The NHS lacks the resources/understanding to deal with autistic mental health crises. I've been accused of everything from lying to being a drug addict by NHS staff when trying to get treatment.”  “I ranked based on ability to implement. There are many small things we can do that. Quite cheap too. It is a shame not to do it. A text support service for autism sounds like a lifesaver. I would use”  “Autistics in crisis needs to be understood - Employing autistics to counsel others and train NHS staff is needed - good communication and a safe autism friendly space is imperative during crisis”  “I selected many of the training ones and put them as high impact. Many autistic people struggle because they don't know that they're autistic until too late in life.”  “I am autistic and have found that my crisis care has been impacted because of this. I have been dismissed by MH teams as they do not understand my condition or how it affects me.”  “It's not safe for many of us to say anything to a health professional about being autistic.”  “My cousin, undiagnosed autistic, committed suicide on 6Jan 2025. The doctors receptionist was nasty to her about asking for a quicker appnt to see the mental health nurse, and she went home and hung h”  “Camhs MH practitioners assessing kids in crisis don't consider masking or situational muteness. Only therapy offered is CBT (son discharged before even that offered) despite suicide notes and running”  “Feel priority should be services autistic people can access in crisis in terms of suicide prevention but also timely diagnosis, training and support in education & workplace can make a huge different”  “Spotting signs of trauma, not just autism, would be utmost for me.”  “Availability of services when autistic person is in crisis - poor. Lack of understanding of autism by GPs & A&E staff problematic. Seeing alcolism as cause rather than symptom prevents access to servi”  “My niece is autistic in crisis. The places she has been put in show a complete lack of understanding of autism. As a result her mental health has declined”  “I was diagnosed at 23 and I believe the services available when growing up would still caused mental harm based on my older brothers experience. Diagnosis does not mean understanding either.”  "woman nearly 60. Life has been hell. Diagnosis 2 years ago. relief and trauma. how many GPs and mental health workers FAILED to spot my autism? How many F&F now challenge me thru ignorance? Woeful!"  "Early recognition is vital to mental health. Most NHS staff even in mental health settings don't recognise it, especially in women/girls/adults in general! Support sooner could stop suicide.” |
| 1. *Access to diagnosis and post-diagnostic care*   Reflected in 26.6% of comments within the parent theme (58.6% of which were from formally diagnosed autistic participants, 30.8% from possibly autistic participants, and 10.6% from non-autistic participants).  [Back to the top](#Thematictable_staging) | “I was diagnosed at 44 but have had no follow up support. I think if you’re ‘good’ at managing your own mental health and suicidal ideation then the NHS lets you get on with it on your own”  "I never had any support or help to understand my Autism. And this led inpart to suicide attempts. Finding information has been near impossible and I have learnt to cope or not most of the time"  “Early identification and support is key - research shows that MH and well-being in autistic people is correlated with having a positive autistic identity.”  “As a woman who was diagnosed at 57, life could have been so different if bullying at schools was taken more seriously and presentation of autistic girls/women was understood. Knowledge is everything.”  “I think that improving the environment is most important. It's also really important to identify people who are undiagnosed and provide appropriate mental healthcare for all autistic people."  “Our experience is that my husbands mental health significantly improved after diagnosis. Priority should be diagnosis and then service adaptations”  “Services are severely underfunded. The waiting listctimes for diagnosis is far too long. Mainstream secondary schools are not designed for neurodiverse children and teachers lack knowledge”  “It's very difficult to rank because all of them are so important. But without access to diagnosis, appropriate support and the financial means to make choices then everything else is irrelevant.”  “Thank you for considering us. Society has rejected and laughed at us for so long and there's been little help, none for us women and girls who were ignored and doubted and still not been diagnosed”  “Every turn is difficult when trying to get a diagnosis and support. As a parent, you have to fight for everything and you shouldn't have to. I was told to keep my child away from anything sharp”  “Many of your suggestions are based on people getting diagnosed, but this won’t change anything”  “As a parent and in my professional life (NHS, Social Services, and Education) I have found recognition, understanding, and skills of autism lacking and too long await for diagnostic assessments”  “Autism needs to be identified so the right help can be put in place, but there's no point identifying the autism if there's no autism specific help available.”  “As a late diagnosed autistic adult female, burnout and suicide attempts may not of occurred if I had the support from when I first thought I was autistic. I do not speak on the telephone”  “Early identification can really help. Too many mental health interventions are designed for NT people and can be actively harmful in Autism and in any event, don't address the root cause of distress.”  “Early ID is key to reducing long term trauma = mental health issues. Unadapted therapy can do harm but is needed for trauma. Work capacity is lower so help is needed. Community improves mental health.”  “As a late diagnosed autistic woman, earlier diagnosis and more tailored support might have prevented my suicide attempt”  “Girls and women are diagnosed too late - poor MH. Longer wait times leads to deterioration in MH. Support & therapies NEED TO BE ADAPTED. Focus on strengths and interests rather than deficits helps.”  “Validation of autism is crucial for some people to know and accept themselves. Education and health professionals must be better informed and proactive.”  “Wider reaching and quicker diagnosis is paramount. Early in life, crucial. Prevent child hood issues of alienation and bullying and stop them spilling into and affecting adult life. Financial support”  “Thank you for taking the time to investigate suicide rates in autistic people. Suicide is an option that I consider daily due to my life experience not knowing I am autistic.”  “Suicide prevention needs to be focused on undiagnosed females and diagnosing them because they're most likely to die.”  “Bullying and the ignorance of others has a huge impact on mental health. Knowing why you see/do things differently really helps, for yourself and others, formal diagnosis or not.”  "CAMHS is not set up for autistic children- the system and staff fail completely. Teachers and SLT do not understand autism in girls and cannot cope/comprehend with the fall out of undiagnosed autism."  “shorter waiting times and better testing for females i believe is a priority as many autistic people need support, which sadly no matter what we change, will never happen without a formal diagnosis.”  "So many people go undiagnosed for too long - especially females and ethnic minorities. Going through life undiagnosed is mentally, emotionally, physically exhausting and traumatising = suicidal."  “I think suicide in autistic women and girls is likely related to being bullied and ostracised while not knowing they are autistic. They begin to believe they deserve it and that they are the problem.”  “Firmly believe early diagnosis would have helped mostly but then not enough support for female ASD and certainly not enough employers willing to accommodate”  “Autistic women in crisis often don’t know that they are autistic, get misdiagnosed and treated for the wrong thing, and the treatment doesn’t work or makes things worse.”  “I really think there needs to be more research into diagnostic criteria for women and girls - I think going undiagnosed for 37 years of my life has made my mental health significantly worse.”  “It's very difficult to choose, I'm a late diagnosed woman who was told I can't be autistic because I am articulate. We need medical professionals to understand autism in girls and women.”  “MAKE THE AUTISM NHS ASSESMENT MORE SPECIFIC TO HIGH FUNCTIONING OLDER ADULTS WHO HAVE LIVED DECADES MASKING TRAITS AND BEHAVIOURS.”  “Access to diagnosis more available and awareness to marginalised groups (POC, immigrants, high functioning, women) and awareness”  “Diagnosis and acceptance are the key things.”  “The ideas relating to understanding & recognition of autism come 1st as I lmany late diagnosed so did not access support so no point in support if no one knows they are autistic or can get a diagnosis”  “I think it’s sad I’ve felt different etc and everyone blamed CP”  “I suspect many suicides are completed by those who are undiagnosed and don’t even realise autism is a possible factor in why life is so utterly, impossibly difficult. So more awareness & diagnosis pls”  “As someone diagnosed in adulthood, I feel early diagnosis and consistent support for students to understand themselves and succeed would have made such a difference to my life.”  “Early diagnosis helps understand why the autistic person thinks and feels the way they do. It's absolutely key. EBSA is real. Trying to get an EHCP is a nightmare.”  "The assessment for asd needs to gear up for girls. It's to much geared for boys. And the metal health's services are diabolical, I believe hubs in the community and schools should be the way forward"  “Please include in your list diagnosis support for non white people as they have more issues accessing diagnosis and help than women and women have a lot and you include them.”  “One issue is that older women do not have the opportunity to be assessed. Long waiting lists, the issues with assessing women, but key is we get turned away when we ask about assessment by GPs.”  “It took over 23 suicide attempts for them to rethink the diagnoses I was rejected from when 7 for being a girl. Since I have been able to access support allowing me to do a degree when I barly passed”  “Thank you for undertaking this work. We need to do so much more for girls and adult women in recognising autism.”  “Pls focus on diagnosing women & girls appropriately, de-stigmatisation of ASD in all spheres.”  The choices are based on own experience. Scared/unhappy in school & most employment, always knowing something was wrong. Accidentally discovered AS in a Times article and diagnosed aged 47 (female).”  I struggled in school and university and was put into care, but no diagnosis until I reached 49 year of age. My answers reflect my general life and then school life.”  Mainstream school and lack of diagnosis destroyed my child. My child was sectioned with psychosis after it took me 12 years to get her a diagnosis as she’s a masked girl”  "Diagnosis and crisis treatment comes first for me as a diagnosis can help avert crisis and asd people are often in crisis. Next is others understanding NHS then school. Last is access to care"  “Currently post autism support doesn’t exist for young adults. Just for family. NICE says MH supports must have someone trained to give MH support when that isn’t always the case. Schools don’t help”  “Feel priority should be services autistic people can access in crisis in terms of suicide prevention but also timely diagnosis, training and support in education & workplace can make a huge different”  “I think education for all ages is vital. Maybe I wouldnt have felt so broken if I had known I was autistic”  “I am 50 and would have benefited massively from identification and/or support as a child. Support, including via peers, would help as an adult. Other people understanding autism would also help."  “Honestly, we need all of these things and more. It took multiple mental health crises for me to be diagnosed autistic at 20 years old as a woman, after going through many NHS mental health services.”  “The options above target people who already think they could be autistic, or for whom it’s been confirmed. But as a late diagnosed woman, I was my most suicidal when had no idea. More early support!”  “My choices reflect my current life, i.e someone who has been waiting 5 years for a diagnosis and is currently being forced to undergo benefit checks designed to trip up claimants rather than help”  “There was much research done when J was little to understand why he had tantrums when he was little as there seemed to be no specific trigger. My niece spent years going to different specialists”  “Presently crisis intervention is priority but prevention should be the aim. NHS support resources are nonexistent, assessment situation is even worse. Many cannot get diagnosis or mental health care”  “Wanting a diagnosis is oddly stigmatised, but gives us an important answer for why we struggle, and helps us stop internalising so much shame and frustration.”  “The choices are based on own experience. Scared/unhappy in school & most employment, always knowing something was wrong. Accidentally discovered AS in a Times article and diagnosed aged 47 (female).”  “diagnosed at 62 years (female ) life of carnage, on Valium at 11 if only I knew I could have had a power of attorney in adult life. . 2 divorces 3 sons later. let down by GP’s”  “It was very challenging, as all the ideas were good. I think the most critical thing is getting people diagnosed and supporting them through the process. Spotting it early is also important.”  “The vital part for me is that NHS needs to deliver MH support that works for and understands he ND brain.I was in the care of CAMHs and CMHT on and off for about 19 yrs before I got diagnosed at 32”  “Better access to diagnosis - remove need for self diagnosis. All educational situations should be geared to support all equally, not identify special needs.”  “My brother died aged 25 in 1991 jumped off clifton suspension bridge. No one suspected Autism, never picked up. He was very bright but " didn't fit in this world, never designed to be this way"  “It takes so long to get diagnosed at the moment, I think that we should target th population at large. My father was not diagnosed and was autistic and killed himself.”  "Short term the lost generation are still being let down by long waiting lists, lack of pre and post dx support.”  “Long term, education and awareness and understanding across the population is key.”  “Early diagnosis is a key to allow autistic individuals re-design their lives. For those who reached burnout, the accessibility to support is a major issue currently.”  “I have heard many accounts where suicide attempts are made by people (especially women) who are undiagnosed and did not know they were autistic. Everyone should have access to support reduce the risk.”  “I think the most important thing is finding undiagnosed people struggling with long term mental health conditions. We are dying from not being diagnosed and no one will even know the true statistics.”  “Start early, train teaching staff, ALL nhs staff & police to recognise autism in children (& adults until it’s normal) INC WOMEN so everyone has access to help asap & suicide goes down in ASD patients”  “A lot of these questions relate to the slow NHS diagnose of autism. A diagnose should be the gateway to these services, if only older people could get one.”  “I feel identification and support/resources for autistic people to understand themselves will be the best way to counter high suicide rates. Raising awareness of nuerodiversity generally willalsohelp”  "I never had any support or help to understand my Autism. And this led inpart to suicide attempts. Finding information has been near impossible and I have learnt to cope or not most of the time. I am 65"  “I am an autistic co-trainer for the Oliver McGowan mandatory training in the NHS. My late diagnosis meant I spent 9 years on medication I did not need and when I got my diagnosis I received no support”  "I think staff in services should be on the lookout for people possibly autistic. Services for autistic should be restricted: selfdiagnosis not accurate:not fair on those people to miss correct help”  "diagnosed late (56) if I’d know earlier in life I think my mental health struggles would have been less, especially if I had had the right support. Even older are helped by diagnoses”  "Generally, I think that appropriate training is the biggest issue for those who work in education, the working environment and health services – to be sensitive to recognising someone who may be autis”  “Iq of over 130, missed diagnosis till almost 30. Been told by GPS I wasn't asd, on and off SSRI's, turns out I am. The only reason I didn't stage exit left, was people that I cared for. Keep this up!”  “None of the autistic people I’ve known who attempted suicide had been diagnosed when they did it. Often undiagnosed people are in a more extreme situation *because* of their lack of diagnosis.”  "Address the root problem: Education, bullying/school life, diagnosis - reduce the number of autistics traumatised before they reach adulthood, and ill-equipped for independent life. Financial help."  “I struggled in school and university and was put into care, but no diagnosis until I reached 49 year of age. My answers reflect my general life and then school life.”  “Waiting times for assessment and diagnosis are very long. By the time someone gets to crisis, or needs support they may not have been assessed. Not all autistic people desire a formal diagnosis.”  "Early recognition, intervention, & support is essential. The earlier autistic people understand themselves, the better. Validation, understanding & support from others is also key."  “groups miss out people who are struggling who do not realise they are autistic (me most of my life)”  “Not knowing you're autistic can easily lead to poor mental health, better knowledge, easier access to a diagnosis and support I feel is essential for helping autistics  “Suicide prevention needs to be focused on undiagnosed females and diagnosing them because they're most likely to die.  “I autistic person who is still currently waiting for assessment. I have been fortunate to have amazing employer who has support my job as a staff nurse. I been the lucky one, many people don't have  “Assessment waiting lists are so long (over 5 yrs locally), need education for GPs, s, MH to not make things critically worse, eg reacting to overwhelmed frozen people with anger, restraint and tranq  “I tried to balance the need for immediate help with things that would help to prevent the crisis in the first place. I think that identifying and supporting people at an early stage is important.”  “Am 70 and awaiting formal diagnosis after grandson & daughter both had recent formal diagnoses. For myself it's a minefield. Understanding & supporting them is difficult while I come to terms with me”  “Autism is similar to any issue, it increases with poverty and intersectionality and trauma. All middle aged adults have not had access to diagnosis or can’t meet the costs of private assessment”  “This is a dilemma for me because I was born in a age when autism was only considered if it was non verbal or severe, I was not formally diagnosed until age 40, self diagnosed at 35, diagnosis is key.”  “Services are severely underfunded. The waiting listctimes for diagnosis is far too long. Mainstream secondary schools are not designed for neurodiverse children and teachers lack knowledge  “Getting diagnosed is high as so many people go through life not knowing what is "wrong" with them which leads to feelings of worthlessness. I was late dx at 42 and it's really helped my self esteem”  “There are so many people coming to discover autism much later in life and it is disorientating & complex. We need support to adjust, reframe & relearn how to look after ourselves eg not typical advice”  “I am a late diagnosed female autistic adult, No understanding from education, mental health struggles, self advocated. Little support from workplace. Burnout. Now trained in therapy to support others.”  “Early Id of autism is essential in so that coping mechanisms and aids can be learnt and practised from a young age so they are more habitual, which will naturally be better engrained in adulthood.”  “undiagnosed autistics often have a harder time than diagnosed as there isnt an explanation for their differences. Only autistic people can know how to help others, people w/o it are often useless”  “Based on my own personal experience. None of the professionals I encountered over the years with my mental health issues mentioned autism. Had to pay privately.”  “early identification, diagnosis and support could help prevent autistic children from depression and suicidal ideation later in life.”  “I never sought assessment myself but believe I am autistic and have 2 children diagnosed autistic. Scottish Education supports needs without diagnosis but its is important in other areas.”  “Everyone thinks they’re autistic” is damaging, but self-diagnosis is where it starts for most adults. Support while awaiting diagnosis will motivate govt to better fund diagnosis services.”  "I am 50 and would have benefited massively from identification and/or support as a child.”  “Support, including via peers, would help as an adult.”  “Other people understanding autism would also help."  “Waiting around for diagnosis is very hard. I’m experiencing this as a woman in my 60s having lost my brother to suicide. I work with children who are losing their education due to delayed diagnosis.”  “My child has had an awful time and due to diabolical lack of funding / support/ knowledge/ wait time we had to go private. I suspect I also have it but mask.”  “Mental health services were high on my rankings as I’ve found there to be little understanding of autism, I went undiagnosed for years. Feeling misunderstood makes things worse.”  “Mainly NHS related choices because my experience with NHS MH services has been terrible. Staff often don't know what it actually is and dismiss autistic girls as anxiety. NHS adult autism service good”  “It took me a suicide attempt before I got the psychological support I needed and a psychologist finally recognised me as potentially having autism. I attempted suicide because I felt incapable in life”  “Assessed aged 58 my life would have been easier with early identification and support to find an appropriate career. I got neither and my life slowly disintegrated.  “I always knew I was different and was told I was mad I learnt to mask the real me and just fit in.it is too late for me but wanted my child to not struggle like I do,they have a diagnosis and help  "stats will look better if diagnosis doesn't occur. care will be greatest when diagnosis does occur. Focus should be on care not stats. Principal of prevention rather than 'cure' should be implemented."  “I discovered autism at 19 and then i was diagnosed at 22 and i think my life would have been totally different and i would have experienced less trauma and struggles with mental health.”  "problems start at school so that's where intervention needs to begin. When a child starts to self exclude then some assessment should start at that point as they begin to be treated as badly.”  "I suspect many suicides are completed by those who are undiagnosed and don’t even realise autism is a possible factor in why life is so utterly, impossibly difficult. So more awareness & diagnosis pls”  “People on autism pathway need help because it is long wait. Transitions are major suicide risk factor. I have slipped through the cracks and it is bad mentally.”  “focus on undiagnosed support. Making sure people don't get forgotten by systems and revive care without self/family advocacy and understand timelines of this with regular checks and updates.”  “Not everyone knows they are autistic at first. I had mental health issues as a teen due to autistic burnout, then realised and got a diagnosis of AuDHD”  “Early diagnosis & support is important to help prevent a MH crisis”  “I think a lot of autistic people don't go diagnosed for a very long time. access to diagnosis is impossible. and accessing it is not very autistic friendly. especially when you have past trauma as wel”  “Probably many undiagnosed autistic people out there. Need to find and help them so get waiting times down too.”  “helping with everyday tasks is often overlooked but can help people a lot. Also support for people in the workplace and education is so important and diagnosing more women also important.”  “Autistic People can actually flourish with the right help and support. Diagnosis needs to happen faster shame of thinking you are a defective Horse when you are a perfectly average Zebra builds!”  “Diagnosis is so hard to access currently so no matter who thinks they have autism, they need to have access to help. Education is also absolutely vital. I wouldnt have thought I was so broken”  "There is very little support out there for autistic mental health, help those people first then they can help others.”  “Earlier identification would mean more chances to be supported/learn strategies”  "It takes years to get diagnosis or be considered for one. If you are Autistic that "no man's land" is a dangerous time. With no support you're more likely to follow through with suicide.”  “For 35 years I have used GP services for mental health , attended eating disorder clinics, an unrelated appointment with a nurse who suggested I may be autistic led to my diagnosis at 53”  “Making sure people are diagnosed is priority number one, knowing there is high likelihood you are autistic changes the pressures you put on yourself to perform to neurotypical standards.”  "I think that improving the environment is most important.”  “It's also really important to identify people who are undiagnosed and provide appropriate mental healthcare for all autistic people."  “The main issues are receiving diagnosis, so that life starts to make sense, and support, so it feels like there's a positive way forward.”  "Early identification and support is key. My experience is that a lot of the suicidal ideology stems from trouble communicating and being understood. Also, stop letting allistic people speak for us."  “I’m currently self diagnosed awaiting assessment. I have 2 kids both autistic. I’ve always struggled finally makes sense to me and this is what I feel I need now”  "I think starting with supporting children would help them to need less support (emotionally) as adults.”  “Many professionals don’t understand autism in females. Waiting times are too long for adults.”  "I was late diagnosed in highschool (female) and if I'd been diagnosed earlier it'd have been better. I was severely bullied and didn't know why. now the NHS won't help me. Im 'too complex' for them.”  “I just wanted to add, Also check for autism when a child tells an adult more than twice that's she's being bullied.”  “I was actually having trouble with the phone version of this form, but I put better understanding of autism in women in girls due to personal experience. I wasn't diagnosed until I was 40.”  “I work in education - 20+ years. Generally speaking, school staff recognise signs of autism. But ch’n can’t seem to get an official diagnosis unless they have a severe learning disability.”  “Diagnosis in the UK is hard to get, especially for people who fit into certain groups. It seems the system is trying to prevent certain people from being diagnosed. Support should be for everyone.”  "many people struggle undiagnosed till later in life I at 47. people who comit it crimes may have drug or alcohol dependence and spectrum problems un diagnosed. if recognised repeat offending could be p"  “Early diagnosis and support is key, to improve self-esteem, coping, and friendships early in life”  “I think it’s a priority to improve accessibility and speed of formal diagnosis for autism so that everyone who needs it can access the necessary services”  “My husband & I were diagnosed with autism in our 40s. We sought diagnosis after our son was diagnosed with autism. Both of us have had suicidal thoughts.”  “I feel like the options were repetitive and fed into each other so that was hard to rank. The main issues are the wait times, the lack of any support especially for adults, and the benefit criteria.”  “Of utmost importance in our families experience- camhs needs to improve recognition of autistic traits and refer for assessment! Trauma caused by them not doing this.”  “Late diagnosis and long wait times leads to depression, burnout and despair.”  “The single most important thing is to identify those who are potentially undiagnosed autistics who are in crisis eg they have approached mental health services in crisis.”  “My main thing is finding those who aren't diagnosed/don't know enough about autism to self-diagnose so feel broken and don't understand why or why 'normal' therapy and techniques aren't working.”  “Autistic women and girls have been overlooked for too long. This is beginning to improve but there is a way to go. hence, I put it as the priority.”  "The waiting time for diagnosis is much much too long. Early intervention is needed - Camhs are overwhelmed and schools are allowed only one ED Psych visit per term. Please help change this"  “If you want to stop people who are autistic killing themselves then there has to be someone "safe" to turn to when you're in crisis. I do not have a diagnosis.The waiting time for assessment is 3 y”  “I think a lot of suicides is because of undiagnosed autism or misdiagnosed as other conditions I think its important that autistic people can obtain services that are helpful to them”  “Developing an understanding of Autism and how it might influence your needs was without a doubt the most important thing i needed to begin accepting myself, and this makes support with education key.”  “I think spotting autism in children and getting them diagnosed early is key to preventing significant mental health issues as an adolescent and adult.”  "Rapid diagniosis will massively save lives. Going through a prolonged autistic shut down without knowing what it is, is incredibly damaging. Support for autistic people in crisis FROM autistic ppl"  “Its so important that support is accessible, that there are options other than death, and that there are more opportunities for undiagnosed autism to be picked up so people can see there might be hope”  “I have to work full time to survive financially but I can't cope with it. I didn't know I was autistic until my 30s and am now on a years long waiting list. I fear I will eventually commit suicide.”  "All students at school should be assessed for autism, (AQ Test) even the ones 'without problems'. It's not rocket science. Then actually provide services that can be accessed at all stages of life.”  "Due to family history of suicide attempts, I believe in early identification/diagnosis and access to timely and suitable support across all sectors for mental health issues for suicide prevention.”  “I believe many autistic individuals are still overlooked or misdiagnosed. I also believe that support systems and programs designed and implemented by autistics may be more effective.”  "I am awaiting diagnosis but since being open to considering that I may have autism I have begun to have so much more insight into events in my past including a time when I planned to take my life.”  “A well trained GP and support network beyond within the mental health care system and education is vital to the wellbeing of a person of any age being correctly assessed then assisted to function.”  “Now my daughter is diagnosed I can see that my brother and father were also autistic and suffered by never being diagnosed, both medically, both went to prison, both found day to day life hard. Died y”  “I selected many of the training ones and put them as high impact. Many autistic people struggle because they don't know that they're autistic until too late in life.”  “There needs to be better diagnosis of autism. I wasn’t diagnosed formally until I was 11, and that diagnosis is crucial for help in other areas falling into place.”  “I'm sorry if it looks like I don't care about children - I absolutely do, but I'm 41 and the idea I might be autistic had never even crossed my mind until the psychiatrist suggested I might be.”  “The time pre suspicion and diagnosis was worst for me regarding suicide. I suffered from complex trauma, as a result of undiagnosed ASD. I now accept it is part of me and normal, so suffer less.”  “Finding it slightly confusing but what do know is I’ve always felt different and struggled with socialising etc, people say it’s bad to have a “label “ but isn’t it just as bad to feel alone etc”  “I was diagnosed aged 18 when a mental health professional recognised female autism. Early diagnosis would have been useful but help with life after education e.g. Work transition was crucial for me.”  “30+ years of being undiagnosed and not knowing what the problem was did the damage to me. Educate/diagnose kids/teens early, get them the support/care early, stop them reaching crisis as an adult.”  “I am a female who was diagnosed with an anxiety disorder at 13. I have experienced 2 severe depressive episodes with suicidal ideation. I am on a 3-year waiting list for an autism assessment at 28.”  “Daughter’s autism not picked up at school. Not accepted onto nhs waiting list for assessment as “functioning” ie off to Uni. Private assessment by Clinical Psychologist (NHS autism lead) confirmed.”  “Need mental health support that doesn't cause more distress by forcing into groups which are too difficult to deal with. Need someone to recognise it's autism related to get the right help.”  “I became suicidal as I was treated for depression and anxiety so strategies I employed to recover just exacerbated my autistic burnout and made me feel worse about myself. Had I been diagnosed autisti”  “It all starts with correct childhood diagnosis and support followed by long-term support and opportunities”  “I made my choices because I am a late diagmosed female and there is nothing in the way of support and it has been very very hard. If it wasn't for my college being helpful I would have nothing.”  “I suffered due to BPD misdiagnosis. That made me want to die at times.”  "My son was diagnosed at 28 and previously had a severe mental health condition diagnosis. We have fought for support for the last 10 years and the system has failed us. No coordination of services”  "The choices come from my experience of a late diagnosis at 56 and my history of mental health issues that I now understand, plus the experience of my children, who have some autistic tendencies.”  "I was a late diagnosed autistic woman at age of 49, it was a complete shock to me but also perfectly described my entire life.”  “I have received NO aftercare support which is very poor."  “Survival 1st, stop fear(benefits).Stop the bullying,ALSO in institutions and PRESS!Not 'on list'SEEKING diagnosis support desirable.INSTANT support is a must.Peer support skilled YES 121 trained,paid!”  “Spotting signs of trauma, not just autism, would be utmost for me.”  “For my suicide attempt, I called the psychiatrist to get an appointment as I was struggling. I was told it was a 12 week wait. My biggest issues were lack of finances, suitable job, no aut diagnosis”  “Prevention is better than cure. Since I realised I'm probably autistic I feel my mental health is so much better. I wish I'd been diagnosed as a child and people had supported me and understood me.”  “I wasnt diagnosed until I was 35, that doesn't mean I magically became autistic at 35, I just struggled through. I don't believe having/not having a diagnosis should be used to discriminate.”  “I wasn’t diagnosed autistic until I was 49. It would have helped me had I been diagnosed earlier. Support following diagnosis would be helpful. I also need support to navigate the benefits system.”  “I was misdiagnosed and in and out of therapy for 15 years before being diagnosed. There’s no support out there that caters to autistic individuals, more generic support for mental health etc”  “Autistic people are not always recognised as autistic or assessed and diagnosed. The question should always be asked be it education, mental health or criminal justice - Is this person autistic?”  “Children and adults from poor backgrounds (maybe due to multi-generational autism in the family) are far less likely to be diagnosed and supported by the aporaphobic society we live in.”  "woman nearly 60. Life has been hell. Diagnosis 2 years ago. relief and trauma. how many GPs and mental health workers FAILED to spot my autism? How many F&F now challenge me thru ignorance? Woeful!"  “Some of the options seemed to almost duplicate others. Although diagnosed at 57, and wanting more awareness for "mature" autistics, I believe suffering can be more effectively reduced by early diagno”  “It takes far too long to get an assessment. The general public believe that autism (along with its difficulties ‘might get better’ and is poor mental health, of course it is a life long condition.”  “I missed the first questionnaire and was disappointed in lack of suggestions to help support undiagnosed people in the workplace - so need checks for autism if stress or disciplinary issues arise”  “My daughter masked in school but age 6 had lumbar puncture done due to stress building up fluid in her brain. If diagnosed autistic sooner could have been more supported as cannot access life easily”  "Early recognition is vital to mental health. Most NHS staff even in mental health settings don't recognise it, especially in women/girls/adults in general! Support sooner could stop suicide."  “People think you can’t die from autism. But if you’re struggling to get a diagnosis and you can’t get the right support, you can kill your self. So that’s the same as dying from autism. We need help.”  “I tried to stab myself in the heart because I didn't want to live. Cousin and grandfather commit suicide. Intervention is critical but only in the RIGHT WAY. School gp mh i missed my autism. Do better”  “Thank you for asking us. I was missed for a very long time. Multiple burnouts misdiagnosed”  “When I didn't know I was autistic my faults felt bad but I was hopeful for improvement. Now I know there's no improving this. I am sad. If I had known what drove me it could have saved a lot of pain.”  “Old people are important. We missed out on diagnosis early in life and have struggled for decades in an alien environment on Earth, but once diagnosed we can understand things better and cope with lif”  “Thank you for the opportunity to be involved with service improvement. I hope practitioner awareness, access to support and diagnosis will improve soon.”  “Looking back, when I was diagnosed with Depression and Anxiety, the experiences I was describing to mental health professionals were autistic traits, so training to identify seems really important.”  “Most of my problems caused by bad reactions to medication for conditions I didn’t have and told too bad to be given therapy. Decades of refusal to give autism assessment.”  “I helped an undiagnosed autistic colleague who was in crisis last week. It brought home to me how the problems of lack of awareness and lack of diagnosis lead to suicidal intentions.”  “As mentioned, I would have preferred the proposals to be split into long and short-term solutions to mental ill-health. My access to services answers reflects current backlog in autism assessments.”  “We tried to access the "Right to Choose" scheme but sadly it turns out you don't actually have a right to choose as it comes down to funding. Our doctors wouldn't refer out of county for diagnosis”  “I got diagnosed after attempting suicide a number of times! This resulted in a quick diagnosis which I am grateful for as the waiting time is far too long.”  “I am a broken person & acutely suffering because of not having special needs support, or intervention,especially in early childhood, teens or adulthood. Getting a late diagnosis of Autism & many other”  “better mental health support is vital, my mental health issues where before I was diagnosed If ASD had been diagnosed recognised earlier, maybe I wouldn’t have almost killed my self - literally.”  “I lived with suicide idealisation since my early teens, more support/awareness for undiagnosed but autistic children surviving family abuse/neglect is needed! I fell through EVERY safeguarding net!”  "Autism is part of being neurodivergent , especially has links to ADHD. Being neurodivergent can be so Much harder especially without support or diagnoses"  “Please put pressure on the government to increase funding for autism assessments. A 4 year wait isn't adequate and self-diagnosis just doesn't cut it”  “Thank you for doing this research - I have been suicidal whilst waiting for my autism assessment because there have been next to no peer support groups where I can talk to others about my problems.”  “Signs of ASD were obvious when I was a child, but I remained undiagnosed till 57. This was the start of severe life long MH issues. The stigma is terrible, even when seeking help 4 suicidality”  “I am a woman waiting nearly 3 years at this point for an autism assessment. I feel the system is not designed to help those who can ot afford private diagnoses, my daughter waited 12 years for hers.”  “The biggest damage is the lack of diagnosis, the long wait to get one and people's ignorance to what it is like to survive with it.”  "Thank you for a clear and accessible survey. In my view better awareness of undiagnosed signs amongst teachers and mental health services is the key and benefits everyone (neurodivergent or not)."  “Thank you for doing this. I spent the first 47 years of my life unaware of my autism and it nearly killed me.”  “I was plagued with suicidal thoughts and suicidal ideation before I realised I might be autistic. Those thoughts have greatly reduced (= now rare). The understanding of my autism helped me enormously.”  “I would be interested to have a formal diagnosis, but my doctor told me that 'everyone is on the spectrum', the local mental health nurse said there are many children waiting to be diagnosed.”  “Dx bipolar aged 19 after suicide attempt. Heavy meds didn't help at all. Dx recurrent depressive disorder & autism age 25. Big help. ADHD added age 36. Last piece of the puzzle. Life much improved.”  “This is a really difficult thing to live with without support in adulthood. I realise now that my entire life has been a struggle not because I am a ‘weirdo’ but because I have this condition.”  “I would love to know how many people in prison have an ASD diagnosis and how accessible is an assessment.”  “Waiting almost 3 years for assessment. Autism makes sense to me, but validation is crucial. Only reason I’m still here is because if verified I might be able to accept myself. I can’t wait for ever.”  “Our son has been misdiagnosed by EPUT for ten years and they relished labelling him as an addict who caused his MH. We now know- in the words of dr eaton and after serious harms “it’s all Autism”  “I was diagnosed with autism at 16 in psychiatric hospital but they didn’t communicate it to me or give me a letter of official diagnosis only on Mh record. I’m now awaiting re diagnosis.”  “I think education for all ages is vital. Maybe I wouldnt have felt so broken if I had known I was autistic”  “Children and teenagers need to understand themselves and their brains. I spent my childhood feeling alone, like a freak. I wasted my life. We need support earlier for young people.”  “Mental health services need to be better adapted to the needs of autistic people. So many get diagnosed with BPD, CPTSD or Bipolar before autism is considered/recognised!”  “We need quicker discovery and diagnosis of autism”  “Mainstream school and lack of diagnosis destroyed my child. My child was sectioned with psychosis after it took me 12 years to get her a diagnosis as she’s a masked girl”  “Would en beneficial to have a neurodiversity assessment (not separate streams for ADHD/ASD. To assess chidlren and parents (as parents of neurodiverse and undiagnosed). I have been waiting over a year for diagnosis. I have also been referred for ADHD assessment.”  “Autistic adults commit suicide for one of two reasons 1) They are undiagnosed, cannot cope, and don't know why. 2) They are diagnosed and there is no 'suitable' support for them to access.”  “Access to diagnosis and an update in the process [redacted] would massively improve things.”  “Thank you for doing. My husband was undiagnosed and had horrendous time trying to get help for his mental health. He never received any as when he was reviewed they said he was fine”  “Diagnosis should be available in every town, waiting times need reducing.”  “been on antidepressants from age 14. nhs therapist said “I had autistic parts of my brain but not enough for diagnosis” “experienced too many emotions to be autistic” 👎  “The NHS needs to do a lot better in recognising autism, especially in women and especially when we're distressed. The dismissal I faced should not happen, nor should routine misdiagnosis with BPD.”  “I am worried that my diagnosis has been missed even with a private psychiatrist and that private companies will over diagnose. Access to medication is hard.”  “This is sad but amazing to learn that research is being done. Suicide is a daily fear supporting my autistic teen who was diagnosed at 18 after years of suffering & trauma. Thank you”  “Because of the overlap between autism and adhd there should be joint diagnosis, and other family members should have access to testing.”  “I lost my career trying to get support & diagnosis. I’ve been waiting from my benefit claim to go to tribunal for two years. We are 9 months away from losing the home I worked hard to buy. I hate it!”  “Misdiagnosis ruined my life, I lost job, driving licence, freedom, and put on antipsychotics. I was just autistic all along. Better education needed for clinicians. I was turned away in crisis. Trauma”  “High functioning 1960's girl, clearly 'odd' from very young but never diagnosed. It runs through our family. We do not need 'extra' help if neurotypicals are not arses to us! Educate them.”  “ I need to be assessed but it's extremely complicated and overwhelming and the wait times are long. I have been extensively "peer reviewer" by diagnosed autistics. My nephew is autistic (delayed dev).”  "And FYI because of the effort to get an adult diagnosis and the waiting times I may never bother getting diagnosed properly.”  “How many people think like me? How many of us are out there?"  “I was the last person to know I am autistic, even my GP suspected years before I did. Finding out so late nearly cost my life, it robbed me of decades and I struggled. This work is important.”  “Early diagnosis is so important”  “Northern Ireland is miles behind in adult diagnosis by the NHS. There is not even a waiting list you can be put on for adult ADHD, no such list exists”  “Diagnosis and acceptance are the key things.”  “If it had been picked up when I was an overachiever who struggled with social groups that I could be autistic, and received the support I needed, my life would look incredibly different right now”  “I was late diagnosed but now.relaise that the periods of severe depression that I have experienced in my life are the result of burnout through masking. Earlier diagnosis might have made a big differe”  “i think certain resources should be safeguarded so they can’t be exploited. Spotting signs of neurodiversity needs huge focus especially in education at all levels.”  “My partner was misdiagnosed as mentally ill when he was autistic. He got failed and killed himself 9 weeks ago.”  “I was diagnosed after suffering mental health issues my whole life and suddenly the issues I'd struggled with made so much sense!”  “This is VERY important. Too many preventable suicides. If my grandma was diagnosed I suspect she’d be alive, nor alcoholic. My brother - alcohol to cope, preventable schizophrenia. My friend’s teen.”  “Mental health services have made my mental health so much worse in various encounters over the years by failing to recognise my autism”  “You need to do more to make people diagnosed with personality disorders aware that autism may apply. This would prevent suicides and self harm. Once diagnosed life makes sense”  “Early diagnosis will help avoid stigma such as lazy, careless and stupid all labels put on me to this day still cause me issues”  “I hope that this information can help others who may have been misdiagnosed and continue to struggle with their mental health and being in a cycle of crisis”  "Looking back, my suicidal ideation was due to being an undiagnosed autistic having constant meltdowns, burnouts and extreme difficulty functioning in life.”  "I’m convinced all NDers are trauma victims. Identifying this over autism alone would be more effective”  “My son took his life self medicating to cope with neurodiversity he didn’t understand it & either did we ADHD shd also be surveyed as in my limited research I found those who took lives also hd adhd”  “Current access to autism assessment on the NHS is unacceptable. No 'right to choose' option in Scotland means it is worse here.”  “We need autistic diagnosticians only... I have seen far too many autistic individuals misdiagnosed or not at all because the clinician was NT and not equipped for the position.”  “As a parent and in my professional life (NHS, Social Services, and Education) I have found recognition, understanding, and skills of autism lacking and too long await for diagnostic assessments.”  “Adults should have access to autism assessments.”  “I was misdiagnosed as sexually abused as a child when I wanted to ceased living for the first time. I wasn't abused, just autistic with no learning disability”  “I also feel there should be more opportunity for people who have had an Autism assessment to complain of ask for a reassessment to be done. I am struggling to get a reassessment. I’m not being heard.”  “Please consider poorer autistic people. All prisoners should be screened for autism and then heavily supported if they receive a diagnosis.”  “I was not diagnosed with autism and have been sectioned twice and hospitalised twice due to psychotic episodes.”  “I waited for three years for an NHS Autism assessment. In the end I was diagnosed privately. This has excluded me from an post diagnosis support from the NHS. I feel this is so wrong.”  “Al I've got to say I struggled all my life thinking what is wrong with me. Braking down in jobs. Couldn't have a intimate relationship. Never learnt to drive. Socially not been out for 22 years"  “I suspect I have ADHD & this is more elevated than ASD. England has RTC, in Scotland the only right we have is to consider which method of payment to use to obtain a diagnosis leaving us behind.”  “More supports needed in employment - reasonable adjustments refused as I didn’t have a diagnosis but no checks done - I didn’t know I was neurodivergent” |
| 1. *Suitable educational provision and transition support*   Reflected in 8.3% of comments within the parent theme (55.4% of which were from formally diagnosed autistic participants, 18.5% from possibly autistic participants, and 26.1% from non-autistic participants).  [Back to the top](#Thematictable_staging) | “Me and so many autistic people I know feel absolutely useless and without hope at this age (I'm 21, my friends are 17-22) because we feel burnt out and lost after leaving school/college”  “Autistic children’s families are being threatened with fines and prison because they’ve made mainstream school inaccessible for autistic children”  “I am an autistic female and suffered a lot of bullying from teachers and students during my education, eventually dropping out at 14. I think the right support in education is a very important factor.”  “I am a broken person & acutely suffering because of not having special needs support, or intervention,especially in early childhood, teens or adulthood. Getting a late diagnosis of Autism & many other”  "Training in education /nhs is poor the excuse is no funding available there is little communication with other groups/ agencies involved. There is little support when young people exit education”  “We need an education system that is flexible and can meet the needs of autistic kids without requiring them to exhibit severe signs of trauma before providing accommodations and alternative routes.”  “Education are letting neurodiverse kids down, they will say it’s health but then don’t take the advise given. School is why my son tried to kill himself. Then college and university no protection”  “My daughter found it hard to make friends and was bullied all through school. She struggles with her mental health and self harms. She went through uni and I encouraged her to go to debate classes.”  "Thank you for a clear and accessible survey. In my view better awareness of undiagnosed signs amongst teachers and mental health services is the key and benefits everyone (neurodivergent or not)."  “Mainstream school and lack of diagnosis destroyed my child. My child was sectioned with psychosis after it took me 12 years to get her a diagnosis as she’s a masked girl”  “My son's suicidal feelings stemmed from overwhelm/burnout in mainstream education. He is now in specialist school and doing well.”  “I’m a teaching assistants . Neordiverse teens are in desperate need of support. Staff aren’t trained to care for them . I struggle to educate my team as im autistic: communication barriers.”  “My 14 year old son with autism took his own life in March 2024. Academic struggles at school & harassment of our family by social work played a massive part in his death”  “Early diagnosis helps understand why the autistic person thinks and feels the way they do. It's absolutely key. EBSA is real. Trying to get an EHCP is a nightmare.”  “The second a child with autism enters the ableist, inflexible,non nurturing,UK Ed system,they are set up to fail,not achieve potential,&feel less than.How can it be my 7yo said he didn’t want 2b alive”  “Early identification and support is key - research shows that MH and well-being in autistic people is correlated with having a positive autistic identity.”  “I feel there needs to be much more support from the NHS and those in education for people with Autism diagnosed or undiagnosed. Bullying should be taken more seriously.”  “Choices based on journey through life - from education to finding employment, with trained support in place at all stages.”  “The sensory environment of mainstream school causes overstimulation and eventually burn out. If child is forced into schools in burn out, parents are threatened with prison, suicide seems only way out”  “i feel school is where it all starts. there needs to be better support for autistic people in schools, all staff should have mandatory training and support needs to be so much better than it is now,”  "Address the root problem: Education, bullying/school life, diagnosis - reduce the number of autistics traumatised before they reach adulthood, and ill-equipped for independent life. Financial help."  “Autistic people want a different environment than Allistics and emotional support is unseen by Allistics. Children need less trauma in their own schools which will halve the problem.”  “Lack of support in childhood set me up for problems for rest of my life. Support with education and career is vital. Those of us that is too late for understand and safe place are vital.”  “problems start at school so that's where intervention needs to begin. When a child starts to self exclude then some assessment should start at that point as they begin to be treated as badly.”  “Start with schools, school was hell for me as an undiagnosed but obviously autistic girl. The teachers missed all the signs and the students ridiculed me. 12 years later I'm still struggling from it.”  "[redacted] County Council are failing SEND children, these children & their parents/carers need help now”  “thousands of neurodiverse children are out of education in [redacted]”  “helping with everyday tasks is often overlooked but can help people a lot. Also support for people in the workplace and education is so important and diagnosing more women also important.”  "CAMHS is not set up for autistic children- the system and staff fail completely. Teachers and SLT do not understand autism in girls and cannot cope/comprehend with the fall out of undiagnosed autism."  “Autistic people are amongst the most marginalised in society. Neither myself or husband are diagnosed but we have 3 Autistic YP. At ages 18 ( twins ) & 25 all are NEET.”  “Validation of autism is crucial for some people to know and accept themselves. Education and health professionals must be better informed and proactive.”  “Children and young people need a lot of support to enable them to become members of society. Adults need support to be helped to have a fulfilling life with help with daily living or social help”  “As a woman who was diagnosed at 57, life could have been so different if bullying at schools was taken more seriously and presentation of autistic girls/women was understood. Knowledge is everything.”  “A better EHCP system and all autistic parents being given advocates is needed.”  “Schools are forcing burnt out kids into school because of attendance policies. Gov policies seem to want autistic kids to just be normal and toughen and go to school, this makes child feel broken.”  “Autistic ppl have high rates of unemployment and poverty, which is a huge stressor and increases likelihood of a poor living situation. School trauma is the root of a lot of issues for me”  “I have little faith that suggesting more funding will fly with the government. Autistic people need more support into work and more access. Young people need early support about society’s structure.”  “The medical profession, employers and education system need to update their understanding of autism and BELIEVE and not gawomen and girls when they are struggling with ND-rooted mental health issues”  "Personally 'groups' would not be of help. Biggest help would be educating others into acceptance. Teachers don't identify girls who study hard and keep to rules as struggling"  “The choices are based on own experience. Scared/unhappy in school & most employment, always knowing something was wrong. Accidentally discovered AS in a Times article and diagnosed aged 47 (female).”  “There is a severe lack of understanding people need to be more aware from a very young age. Gp and parents, also teachers.”  “School, a FORCED social environment, is the toughest part of life for autistic kids, especially teenagers. For adults it's work environment or unsympathetic benefits ASSESSMENTS.”  “Better access to diagnosis - remove need for self diagnosis. All educational situations should be geared to support all equally, not identify special needs.”  “Thank you for all of the research you are doing as this is very near and dear to my heart. I would love to see more support for autistic people in education and at work.”  “Support to break from work/education and support in these places are paramount. My suicidal feelings always occur with burnout and burnout occurs because schools, universities, work are not inclusive”  “Generally, I think that appropriate training is the biggest issue for those who work in education, the working environment and health services – to be sensitive to recognising someone who may be autis”  "Services are in a dire state at the moment, so it does seem like some of these suggestions are unachievable but also good to see what people have considered. Changes need to start with schools."  “The scars from childhood determine outcomes in adulthood. Deal with education and bullying first. It will significantly improve outcomes.”  “I struggled in school and university and was put into care, but no diagnosis until I reached 49 year of age. My answers reflect my general life and then school life.”  “I am a self diagnosed autistic female who comes from a very neurodiverse background. I also work in primary education & advocate for our neurodiverse children. There 100% needs to be more awareness.”  “I really hope this helps improve access to support when required but perhaps if NHS and education were more aware of danger of suicide, they’d help more rather than sending them to a website!”  “Alternative schools for autistic teens would be best, with smaller classes, staff who understand their needs. Mainstream school is traumatic & overwhelming for autistic teens, leading to suicidality”  “I prioritised financial support and reforming the benefits system. Accessing benefits is intentionally cruel. Next priority: flexibility in work and education. Next priority: more control over our own”  “Services are severely underfunded. The waiting listctimes for diagnosis is far too long. Mainstream secondary schools are not designed for neurodiverse children and teachers lack knowledge”  “Very few of the autistic people I now work with get through education without trauma. There is very little support for autistic adults without learning disabilities.”  “Education need to change to support young people. Social care need to support ASC adults this is not just health it is beyond that”  “Educating people who work in schools is important because early issues could be resolved reducing trauma. Financial and life skill support is also important because burnout could cause more suicides.”  “I grew up with 0 support both before and after my diagnosis. My struggles are invisible to healthcare professionals and I don’t get the life skills support I need. Education traumatised me very badly.”  “Missing: non-mainstream school options, freedom of dress in school + work, certainty of no compulsory treating when go to mental health, opportunities to tell researchrs + policymkrs past experiences”  “my no1 isn’t on list. stop making children fail in school before they get help.”  "I purposely left out education as children should have the right to benefit from the environment in their school set up. Autistic children in main line schools find the environments traumatic."  “Autistic children need time alone. There should not be the need to threaten parents with fines for their autistic child's non attendance. Ofsted cause teachers to focus on other matters not autism.”  “i think certain resources should be safeguarded so they can’t be exploited. Spotting signs of neurodiversity needs huge focus especially in education at all levels.”  "Diagnosis and crisis treatment comes first for me as a diagnosis can help avert crisis and asd people are often in crisis. Next is others understanding NHS then school. Last is access to care"  “Taking pressure off ND children and teens.tge pressures of school and life is breaking our children. More underetstanding , support, flexibility”  “Autistic children need to be taught in a specialist environment where they can learn and be with like minded peers. State schools or best fit private schools are not able to give the right support.”  “Support to autistic individuals in the mainstream education system would decrease trauma to autistic people. Support with daily living would Support autistic people and help them be less overwhelmed”  “Healthcare and education are so important.”  “I have first hand experience of being failed by the edudction system and the NHS. My son has been failed by education. This is where we can make the biggest difference, early on!”  “If more support was provided in schools autistic children may never reach the point of crisis. Too often a child reaches crisis point but hasn't even been assessed by camhs. Everything is too slow.”  “Currently post autism support doesn’t exist for young adults. Just for family. NICE says MH supports must have someone trained to give MH support when that isn’t always the case. Schools don’t help”  “I placed importance on improvements in services available for acute mental health episodes, but a priority was flexibility in work/school as social attitudes are a huge barrier to accessing support”  “Children are let down by schools, CAMHS, where they could easily be referred for diagnosis. Then let down by mental health services as an adult, which includes GP, Psychiatrists, counsellors, CMHT.”  “As someone diagnosed in adulthood, I feel early diagnosis and consistent support for students to understand themselves and succeed would have made such a difference to my life.”  “Feel priority should be services autistic people can access in crisis in terms of suicide prevention but also timely diagnosis, training and support in education & workplace can make a huge different”  “Contacting the Samaritans, they have been very understanding of autism. The police and teachers humiliate autistic speech. When they no longer teach a pupil, teachers should lose all influence.” |
| 1. *Suitable employment and social care in the community*   Reflected in 7.5% of comments within the parent theme (68.7% of which were from formally diagnosed autistic participants, 21.7% from possibly autistic participants, and 9.6% from non-autistic participants).  [Back to the top](#Thematictable_staging) | “I think the most important thing is to improve the ability for autistic people to participate in society and use their skills to benefit their community while still allowing for rest”  “I feel it is important to ensure neurodivergent individuals are recruited into different job roles aligned to capability and clear process have to be followed support positive work placements”  “My 14 year old son with autism took his own life in March 2024. Academic struggles at school & harassment of our family by social work played a massive part in his death”  “I ranked it based on what I would need to live a healthy and fulfilling life. I'm waiting on a social worker and I barely make it through day to day”  “Autistic ppl have high rates of unemployment and poverty, which is a huge stressor and increases likelihood of a poor living situation. School trauma is the root of a lot of issues for me”  “Children and young people need a lot of support to enable them to become members of society. Adults need support to be helped to have a fulfilling life with help with daily living or social help”  “Support to break from work/education and support in these places are paramount. My suicidal feelings always occur with burnout and burnout occurs because schools, universities, work are not inclusive”  “Autism villages built. Autism specific social care. Autism sensory rooms nhs"  “Support to autistic individuals in the mainstream education system would decrease trauma to autistic people. Support with daily living would Support autistic people and help them be less overwhelmed”  “A well trained GP and support network beyond within the mental health care system and education is vital to the wellbeing of a person of any age being correctly assessed then assisted to function.”  “Generally, I think that appropriate training is the biggest issue for those who work in education, the working environment and health services – to be sensitive to recognising someone who may be autis”  “helping with everyday tasks is often overlooked but can help people a lot. Also support for people in the workplace and education is so important and diagnosing more women also important.”  “Feel priority should be services autistic people can access in crisis in terms of suicide prevention but also timely diagnosis, training and support in education & workplace can make a huge different”  “I am AuDHD so it is very difficult but I think work support is the most important with peer mentor support.”  “The medical profession, employers and education system need to update their understanding of autism and BELIEVE and not gawomen and girls when they are struggling with ND-rooted mental health issues”  “Firmly believe early diagnosis would have helped mostly but then not enough support for female ASD and certainly not enough employers willing to accommodate”  "The assessment for asd needs to gear up for girls. It's to much geared for boys. And the metal health's services are diabolical, I believe hubs in the community and schools should be the way forward"  “The choices are based on own experience. Scared/unhappy in school & most employment, always knowing something was wrong. Accidentally discovered AS in a Times article and diagnosed aged 47 (female).”  “School, a FORCED social environment, is the toughest part of life for autistic kids, especially teenagers. For adults it's work environment or unsympathetic benefits ASSESSMENTS.”  “Thank you for all of the research you are doing as this is very near and dear to my heart. I would love to see more support for autistic people in education and at work.”  “I prioritised financial support and reforming the benefits system. Accessing benefits is intentionally cruel. Next priority: flexibility in work and education. Next priority: more control over our own”  “Education need to change to support young people. Social care need to support ASC adults this is not just health it is beyond that”  “I grew up with 0 support both before and after my diagnosis. My struggles are invisible to healthcare professionals and I don’t get the life skills support I need. Education traumatised me very badly.”  “Autistic people particularly when highly intelligent struggle with expressing their feelings, social interactions and finding and worthwhile work/employment. This leads to feelings of worthlessness.”  “Majority of NHS staff not trained to deal with autistic people. Very little support available after diagnosis. Employers fail to address the simplest of issues, which could improve work and life.”  “I'm a nurse just going through restructuring, as an older diagnosed autistic I have been taken through a very unsupported process. I'm not that personable, in a click so I don't charm or stand out.”  “I dont believe self-diagnosis, it must be medical. Prevention of suicidal thoughts key, hence work+life opportunities critical: secondary is rapid diagnosis: last is crisis management.”  “As someone autistic, I've had a lot of difficulty finding suitable work. Highly skilled technically but too anxious and don't have the social skills/experience to get roles. I imagine I'm not alone.”  “Misplacement is unaddressed. "Supported living" is first used to unburden functional relatives. To prop up an autist among non-autists with only food and shelter in helpless bewilderment is torment.”  “I work in domiciliary care. I got bullied by a patient. I was told by manager to go in other room and cry union said the employer didn't have do anything. I have asked for autistic mentor not coping.”  “I have problems in interviews for teaching assistant jobs because I am literal, because I am passionate but not the usual kind of teachery bubbliness. They want formulaic answers which I can’t provide”  “Hard to eliminate/rank as so much needs to change. Addressing NHS numerous shortfalls for immediate effect. Quality of life for long term effect. Rounded approach needed.”  “I autistic person who is still currently waiting for assessment. I have been fortunate to have amazing employer who has support my job as a staff nurse. I been the lucky one, many people don't have”  “Helpful for work places to know how they can support autistic people so that the person isn’t mentally exhausted at the end of the day”  “Educating people who work in schools is important because early issues could be resolved reducing trauma. Financial and life skill support is also important because burnout could cause more suicides.”  “I am a late diagnosed female autistic adult, No understanding from education, mental health struggles, self advocated. Little support from workplace. Burnout. Now trained in therapy to support others.”  "I can't access healthcare and there is no appropriate mental health care for me. I've been denied benefits. Social care doesn't offer anything useful. Early support would mean fewer traumatised adults"  “There is nothing after diagnosis except when in crisis and advocates have to seek this out. There is nothing after official (certified place) with autistic employees in any institution to support”  “I'm concerned about the impact of arrest and unemployment on autistic people, as those have been big events in my recent life.”  “Assessed aged 58 my life would have been easier with early identification and support to find an appropriate career. I got neither and my life slowly disintegrated.”  “I ranked it based on what I would need to live a healthy and fulfilling life. I'm waiting on a social worker and I barely make it through day to day. If I had intervention sooner, I would be happier”  “I've a BSc (Hons, 1st class, applied psych). Yet work outside the home destroys me: insomnia, panic attacks, self-harm, SI. My ASD teens (mild LD range) = same issues. We need £££, family PA, cleaner.”  “There are too many steps to access NHS or financial support. Because of my autism this is overwhelming. I have no access to autism-specific support. I feel this is a main factor in my unemployment”  “As well as support services for "functioning" in society, it's important to acknowledge that our needs & capacity are different to neurotypicals (hence breaks from work/school) - we need both”  “The priority should be to helping them in their normal life. These day-to-day struggles are what accumulates to the major feeling of loss of hope & desire to continue living. And parents need this too”  “To reduce suicide ideation, autistics need to feel much less alienated. We need help to allow us to function more fully as part of society.”  “The best way we can stop suicides is by making lives worth living - friends, lovers, community, financial security via adapting the world of work to accommodate autism.”  “I struggle with depression and suicidal ideation because with autism and chronic illnesses I find it impossible to keep on top of and do daily activities.m without support of others.”  “The biggest impact on my mental health has been managing as an adult financially. Burnout, relationships. I work with autistic children”  “Work is the single biggest stressor for me. Autism, if you're able to work is a hidden disability. We NEED LEGAL protection for autistics at work same as any visible disability”  "Autistic involvement is key ....insight! Support from another autistic person.... people feel they are not alone. Help with executive function....so overwhelming. Time off at crisis points."  “I am self diagnosed, awaiting formal diagnosis and have struggled to get support from my employer. I’ve also experienced pushback from colleagues who don’t understand my needs.”  “All autistic need advocacy of their choice . Mentoring. Access to living options without group living , sensory, counselling and daily living, financial support .subs open 24hours villages built.”  “Early ID is key to reducing long term trauma = mental health issues. Unadapted therapy can do harm but is needed for trauma. Work capacity is lower so help is needed. Community improves mental health.”  “I am AuDHD so it is very difficult but I think work support is the most important with peer mentor support.”  “I mostly thought about my own experiences and what I think could have prevented my struggles. E.g. a mental health service that understands autism and an accessible working world.”  "More Help From Adult social care If u move To an Supported home"  “There was no support after my diagnosis age 53. I was shocked and having to reassess my whole life. In areas near me there is significant support but I can’t access. More help in workplace”  “I placed importance on improvements in services available for acute mental health episodes, but a priority was flexibility in work/school as social attitudes are a huge barrier to accessing support”  “In my experience those who have power such as NHS, schools, and workplace mangement have inadequate understanding and just view any training as a tick box exercise.”  “Financial support above all. Most suicidality is caused by housing insecurity & poverty, which affects autistic ppl more than average. With that baseline support in place other issues can be addressed”  “Fulfillment and meaning are so important to autistic people, and if we feel fulfilled and that our lives are meaningful then it will make a huge difference in regulating mental health.”  “For my suicide attempt, I called the psychiatrist to get an appointment as I was struggling. I was told it was a 12 week wait. My biggest issues were lack of finances, suitable job, no aut diagnosis”  “The waiting lists are so long that undiagnosed people wouldn’t get help otherwise. I have no idea how to ‘do life’. I have no idea how to apply for benefits. The forms for autusm are too hard.”  “Bullying in school prevents autistic ppl from evolving any social skills. It also drags into workplaces where we can't select who we have to work with. Also, make public spades more autism friendly”  "My worst point was when I was arrested after a very major autistic issue, i then lost my job. This led 2 a downward spiral. In my current job I often need breaks, and being interrupted is a nightmare"  “Choices based on journey through life - from education to finding employment, with trained support in place at all stages.”  “I missed the first questionnaire and was disappointed in lack of suggestions to help support undiagnosed people in the workplace - so need checks for autism if stress or disciplinary issues arise”  “an understanding to begin with, people with autism being able to follow what they are interested in and/ good at as well as the understanding of parents and professionals, then risk of suicide reduce”  “I have a masters degree but I have struggled to find work due to social & sensory problems. It means I can't afford to socialise or do my hobbies or start a family.”  “I wanted to end my life twice, one time because of school and one time because of work. The world is not designed for autistics, especially the ones with high sensory issues.”  “I feel it is important to ensure neurodivergent individuals are recruited into different job roles alighed to capability and clear process have to be followed support positive work placements.”  “My daughter (mid 30s) in regular crisis. We get no support from Ex Social Care, EPUT or S Ex Asperger’s Service & she was bullied by Housing Dept from 2019. H’less. Under confidentiality. Im helpless.”  “People who autism feel that don't have a purpose or struggle to have a job. I feel concerned they would lose benefits and having to reapply”  “i’ve been suicidal as long as i can remember. struggle to make friends, feel like an alien, sure i’m going to crash out and quit my first adult job i got at age 38”  “Employment is key to independent living. Normalise part time working for autistic people”  "I have struggled more with work since diagnosis. Applied to AtW need help with applying"  “It's been work, predominantly, that's made me experience suicide ideation.”  “Even with mental health support I found I was still really struggling in times of crisis. We need pportunities to help us with our hopes/goals, like making friends/relationships and fueling hobbies.”  "Autism villages built. Autism specific social care. Autism sensory rooms nhs"  “I will eventually kill myself because I have to work full time and my needs are not being met and no one cares. If I could get benefits enough to live on I might survive but I know I won't.  “Social services must also be trained on autism presentation !!”  “I have little faith that suggesting more funding will fly with the government. Autistic people need more support into work and more access. Young people need early support about society’s structure.”  "I want To help if I can Social worker people Nedeed to do mired BeforeThey move home"  “Greater support to help autistic individuals remain in work, to support employers or managers who do not understand or use the diagnosis to fire. Burnout & daily guidance mentoring advice.”  “The most limiting factor I've experienced as an autistic individual is not being able to get a job despite obtaining an MPhys Hons. degree. How is this possible?”  “I attempted suicide because I was fired from my job in a brutal way with no thought to how the information was delivered, it sent me into full on failed mode and I walked in front of a car”  “I am really struggling with my job and feeling intensely burnt out. My mental health is very poor. I don’t feel my autism is acknowledged in my daily life or the ways it makes me struggle”  “Diagnosis for me was in a time autism in girls was a new concept. But my biggest struggle has always been the office politics neurotypical people can't seem to live without. This culture harms us.”  “Autistic people need to be loved and accepted for who they are, when they become adults there is nothing to help develop friendships, support at work, my son works full time so needs weekend help”  “More supports needed in employment - reasonable adjustments refused as I didn’t have a diagnosis but no checks done - I didn’t know I was neurodivergent” |
| 1. *Financial security*   Reflected in 5.5% of comments within the parent theme (67.2% of which were from formally diagnosed autistic participants, 21.3% from possibly autistic participants, and 11.5% from non-autistic participants).  [Back to the top](#Thematictable_staging) | “I ranked financial support #1 because too many are stuck in a cycle of recurring autistic burnout (often with suicidality) because of pushing themselves past their limits to make enough money to live”  “Many kill themselves after losing their support network and financial support. They struggle to work or pay rent. The fear is overwhelming. Most live with others and don't work. They need money”  “I feel like the options were repetitive and fed into each other so that was hard to rank. The main issues are the wait times, the lack of any support especially for adults, and the benefit criteria.”  “Autistic ppl have high rates of unemployment and poverty, which is a huge stressor and increases likelihood of a poor living situation. School trauma is the root of a lot of issues for me”  “most services don’t feel like they encapsulate all presentations of ASD, e.g. women, low needs. Also employment/finances is an issue for many autistics, support would help!”  “It's very difficult to rank because all of them are so important. But without access to diagnosis, appropriate support and the financial means to make choices then everything else is irrelevant.”  “The DWP and the benefits system has been one of the biggest causes of my mental decline as well as the current demonisation of mental health and benefits”  “There are too many steps to access NHS or financial support. Because of my autism this is overwhelming. I have no access to autism-specific support. I feel this is a main factor in my unemployment”  “Early ID is key to reducing long term trauma = mental health issues. Unadapted therapy can do harm but is needed for trauma. Work capacity is lower so help is needed. Community improves mental health.”  “School, a FORCED social environment, is the toughest part of life for autistic kids, especially teenagers. For adults it's work environment or unsympathetic benefits ASSESSMENTS.”  "Address the root problem: Education, bullying/school life, diagnosis - reduce the number of autistics traumatised before they reach adulthood, and ill-equipped for independent life. Financial help."  “I prioritised financial support and reforming the benefits system. Accessing benefits is intentionally cruel. Next priority: flexibility in work and education. Next priority: more control over our own”  “Educating people who work in schools is important because early issues could be resolved reducing trauma. Financial and life skill support is also important because burnout could cause more suicides.”  “Wider reaching and quicker diagnosis is paramount. Early in life, crucial. Prevent child hood issues of alienation and bullying and stop them spilling into and affecting adult life. Financial support”  “Many kill themselves after losing their support network and financial support. They struggle to work or pay rent. The fear is overwhelming. Most live with others and don't work. They need money.”  “My choices reflect my current life, i.e someone who has been waiting 5 years for a diagnosis and is currently being forced to undergo benefit checks designed to trip up claimants rather than help”  “Non-autistic people will never understand despite training, we need autistic people trained to help so we really feel heard. Benefits system is designed to drive us to suicide.”  “Financial help is probably the most important, which is easier to apply for. Depression and Anxiety are very prevalent with Autistic people and taking away that pressure gives them room for decisions.”  “My recent, further demise & suffering I can directly attribute due to the designed persecution method & immoral conduct from within the benefits system these last few years. Truly wicked, harrowing.”  “I ranked financial support #1 because too many are stuck in a cycle of recurring autistic burnout (often with suicidality) because of pushing themselves past their limits to make enough money to live.”  “Autism is similar to any issue, it increases with poverty and intersectionality and trauma. All middle aged adults have not had access to diagnosis or can’t meet the costs of private assessment”  "I can't access healthcare and there is no appropriate mental health care for me. I've been denied benefits. Social care doesn't offer anything useful. Early support would mean fewer traumatised adults"  “Right now, what would most make living my life easier would be being able to live with a partner while on benefits. Because I can't, I live alone - that's really hard and makes me sad and angry.  “I've a BSc (Hons, 1st class, applied psych). Yet work outside the home destroys me: insomnia, panic attacks, self-harm, SI. My ASD teens (mild LD range) = same issues. We need £££, family PA, cleaner.  “There are too many steps to access NHS or financial support. Because of my autism this is overwhelming. I have no access to autism-specific support. I feel this is a main factor in my unemployment  “Financial instability was a big trigger for suicidal thoughts for me during university as a full-time student. I never called helplines because I didn’t want to speak. Helping us find community is key  “Wider reaching and quicker diagnosis is paramount. Early in life, crucial. Prevent child hood issues of alienation and bullying and stop them spilling into and affecting adult life. Financial support  “In my personal experience, benefits and finances are one of my biggest burdens. I’m far too confused and anxious to apply for PIP- i can barely make a GP appointment myself.”  “As an adult with Autism who has struggled my entire life i would like to see more support financially as trying to work like a neurotypical has been one of the big stuggles.”  “I put the financial support and benefits ones top because money is a huge cause of stress, and working leaves autistics with no energy for hobbies or socialising which are essential for mental health.”  “I have seen many u diagnosed people with Asd reduced to a life of misery and poverty due to lack of support and understanding.”  “The biggest impact on my mental health has been managing as an adult financially. Burnout, relationships. I work with autistic children”  “The DWP’s failures with how benefits are organised are actually quite a large cause of severe distress in disabled and autistic people. The DWP are clueless on autism”  “I realised in the final ranking you did not include advocates for benefit application in the care corporation advocacy part I feel benefits system ione of thw top 5 reasons for adult autistic suicides”  “1 Helping person in crisis. 2 foundations to prevent crisis (breaks from education, help w benefits). Less important: clubs, self-advocacy classes, help in small way but don't address core issues.”  “My rationale is that trauma begins in how autistic people experience the world and are stigmatised and mistreated from a young age. Then they need financial support throughout life.”  “I also think benefits are important because PIP^[[6]](#footnote-6)^ assessors were discriminatory and disgusting to me. I still have nightmares about my PIP assessment.”  “Bullying was my biggest issue, then sexual abuse, then financial issues and im definetly not the only autistic person this happened to. I have medical trauma probably PTSD due to dealing with the NHS.”  “I think it depends on the age group. For me my main problems as an adult were the cruel benefits system, but as a child it was the bullying, we should aim for improvement in both for both age groups.”  “All autistic need advocacy of their choice . Mentoring. Access to living options without group living , sensory, counselling and daily living, financial support .subs open 24hours villages built.”  “I have to work full time to survive financially but I can't cope with it. I didn't know I was autistic until my 30s and am now on a years long waiting list. I fear I will eventually commit suicide.”  “Key factors: enable people to be able to afford to live, and then get support from peers rather than have those around them make it worse ie bullying, and also reduce systemic sexism.”  “It is very important that autistic people are accepted and are able to actually live and participate in society therefore financial inclusion and access is very important.”  “Financial support above all. Most suicidality is caused by housing insecurity & poverty, which affects autistic ppl more than average. With that baseline support in place other issues can be addressed”  “I am suffering all the time with having to apply for benefits and making sure I don’t go over the means test threshold I think they should remove the means test for we should get more money”  "There are more pieces to my puzzle: I'm high-functioning. It's just enough to ruin your life, but not enough to look disabled to others. I know I can sink. Biggest problems are money and stability."  “Survival 1st, stop fear(benefits).Stop the bullying,ALSO in institutions and PRESS!Not 'on list'SEEKING diagnosis support desirable.INSTANT support is a must.Peer support skilled YES 121 trained,paid!”  “I found that I had sometimes picked the best idea from each group - for example, although more financial support generally would be best, support with accessing benefits would be better than nothing.”  “For my suicide attempt, I called the psychiatrist to get an appointment as I was struggling. I was told it was a 12 week wait. My biggest issues were lack of finances, suitable job, no aut diagnosis”  “The waiting lists are so long that undiagnosed people wouldn’t get help otherwise. I have no idea how to ‘do life’. I have no idea how to apply for benefits. The forms for autusm are too hard.”  “I wasn’t diagnosed autistic until I was 49. It would have helped me had I been diagnosed earlier. Support following diagnosis would be helpful. I also need support to navigate the benefits system.”  “Autistic people struggle with everyday life. PIP does not provide enough in order for people to survive on.”  “The benefits system deliberately doesn't include autism issues, the questions should be redesigned for PIP.”  “The DWP is currently ruining my life. The WCA seems designed to cause harm to n/d claimants. My life was fine 'till I was migrated to UC. My mental and physical health have dropped through the floor.”  “Autism is not a mental health condition. DWP seem to think it can be relieved with medication, it can’t.”  “People who autism feel that don't have a purpose or struggle to have a job. I feel concerned they would lose benefits and having to reapply”  “I think, once people survive education, they then have to survive life. The benefit system is so soul destroying for a non austic person but I'm sure it has been a cause of many suicides I”  “The second most important thing for suicide prevention is probably getting financial help for many of us. I was unable to manage full time work without burnout even when I was well enough to work”  "I feel that finance is almost always the baseline. Secure housing and food are crucial."  “I need financial supportike an EMERGENCY!!! Chrinic migraine loss of speech and movement...thrown off Zpip as I look and sound "Normal"”  “I lost my career trying to get support & diagnosis. I’ve been waiting from my benefit claim to go to tribunal for two years. We are 9 months away from losing the home I worked hard to buy. I hate it!”  “DWP Benefit assessments severly impact mental health, rumination, suicide ideation”  “I will eventually kill myself because I have to work full time and my needs are not being met and no one cares. If I could get benefits enough to live on I might survive but I know I won't.”  “My autistic young person really worries about ever being financially independent but isn't eligible for benefits”  “More help after diagnosis as an adult I have found very lacking. PIP for diagnosed”  “My Child suffered bullying at school but with a lot of support from us they managed to get a degree, but they received no help from the NHS after 16 I feel they have been let down by the NHS and DWP” |
| 1. *Safety from victimisation and equity within the criminal justice system*   Reflected in 6.1% of comments within the parent theme (67.3% of which were from formally diagnosed autistic participants, 17.3% from possibly autistic participants, and 15.4% from non-autistic participants).  [Back to the top](#Thematictable_staging) | “My son has a 2:1 Law degree and is experiencing huge and extensive disability discrimination in the workplace which has lead to his second attempt at suicide”  “The CJS needs serious work as well. I was accused of being a gifted hacker & stalker ... [redacted locality] police said because i can talk I'm not a vulnerable person. Apparently it was reasonable”  “To prevent suicide you need to change attitudes in society so we don't face discrimination and bullying. You need to train staff to understand us. You need to give us ways to cope and support to cope.”  “As a woman who was diagnosed at 57, life could have been so different if bullying at schools was taken more seriously and presentation of autistic girls/women was understood. Knowledge is everything.  “I think suicide in autistic women and girls is likely related to being bullied and ostracised while not knowing they are autistic. They begin to believe they deserve it and that they are the problem.”  “Autistic women more likely to be in Domestic abusive relationships but more likely to recognise those as caring when they are controlling or coercive relationships and if profs not aware feed into it”  “There us an epidemic if police violence against us when we can't communicate under stress. Also perimenopause and pmdd need addressing as they cause high volume of suicides. Nobody warns us.”  “I am an autistic female and suffered a lot of bullying from teachers and students during my education, eventually dropping out at 14. I think the right support in education is a very important factor.”  “There was so much bullying at school. All kids needs to be sensitised to the needs of others, including all educational staff who allowed bullying to take place. Most doctors and therapists the same”  "Address the root problem: Education, bullying/school life, diagnosis - reduce the number of autistics traumatised before they reach adulthood, and ill-equipped for independent life. Financial help."  “The scars from childhood determine outcomes in adulthood. Deal with education and bullying first. It will significantly improve outcomes.”  “Wider reaching and quicker diagnosis is paramount. Early in life, crucial. Prevent child hood issues of alienation and bullying and stop them spilling into and affecting adult life. Financial support”  “Bullying and the ignorance of others has a huge impact on mental health. Knowing why you see/do things differently really helps, for yourself and others, formal diagnosis or not.”  “Contacting the Samaritans, they have been very understanding of autism. The police and teachers humiliate autistic speech. When they no longer teach a pupil, teachers should lose all influence.”  “I feel there needs to be much more support from the NHS and those in education for people with Autism diagnosed or undiagnosed. Bullying should be taken more seriously.”  “My daughter found it hard to make friends and was bullied all through school. She struggles with her mental health and self harms. She went through uni and I encouraged her to go to debate classes.”  “Start with schools, school was hell for me as an undiagnosed but obviously autistic girl. The teachers missed all the signs and the students ridiculed me. 12 years later I'm still struggling from it.”  “I work in domiciliary care. I got bullied by a patient. I was told by manager to go in other room and cry union said the employer didn't have do anything. I have asked for autistic mentor not coping.”  “I feel misunderstood, unaccepted and unwanted by society. By educating society and raising awareness, combined with legal protection, autistic people will find acceptance and accept themselves.”  “I was bullied at school because of being different (basically just quite). It still impacts my mental health 30 years later. My parents and teachers seemed unaware. This hurts as much as the bullying.”  “There needs to be more research into the long term impact that school bullying has in later life for autistics.”  “I was subject to bullying in school and it haunted me all my life, resulting in a nervous breakdown and hospitalisation.”  “i find reducing bullying to be the most important simply because and it can cause really deep, ingrained mental illness that can easily go under the radar and fuck you up for a long time.”  “As a woman who was diagnosed at 57, life could have been so different if bullying at schools was taken more seriously and presentation of autistic girls/women was understood. Knowledge is everything.”  “I had to resign from my role as a Registered Nurse in Rheumatology due to bullying over 6 months. As a result I was in constant fear. I began banking..Mistakes made.”  “AFAB dx'd autistic @ 5, heavily abused between 9-19, pushed around by CMHTs with no help for 7yrs, mis-dx'd EUPD, fucked NHS off, found help thru MHcharity, fought to get dx of complex ptsd after 8yrs”  “The biggest factor for depression in autistic people is rejection. So reducing bullying in schools is the most essential as it may prevent autistic people needing therapy in the first place.”  “I was late diagnosed in highschool (female) and if I'd been diagnosed earlier it'd have been better. I was severely bullied and didn't know why. now the NHS won't help me. Im 'too complex' for them.”  “I just wanted to add, Also check for autism when a child tells an adult more than twice that's she's being bullied.”  “I was constantly bullied throughout education, it has left mental issues that I am still struggling with today. For me, suicide is more worrying over time as I keep losing hope I'll ever feel better.”  “My rationale is that trauma begins in how autistic people experience the world and are stigmatised and mistreated from a young age. Then they need financial support throughout life.”  “Worrying about safety is a baseline threat to Autistic people’s mental wellbeing, especially if this sparks fears of being a burden or ‘useless’.”  “It begins with bullying in schools. Interestingly it takes many years for a 'professional' to identify that you're autistic, but a child of 7 can spot you and start making your life hell from then on”  "Reasons for autistic people to have suicidal thoughts are isolation and feeling different/a bother. Bullying is massive, work/school/in life. Support is scarce and for visible disabilities.mate crime"  “Autistic teenagers are more likely to be bullied than neurotypical teens, and is the main reason for depression and anxiety, and ultimate suicide.”  “Bullying was my biggest issue, then sexual abuse, then financial issues and im definetly not the only autistic person this happened to. I have medical trauma probably PTSD due to dealing with the NHS.”  “Bullying in childhood has lifelong impact on a person self worth, esteem and view of others. tbh it was hard to rank all the choices”  “I think it depends on the age group. For me my main problems as an adult were the cruel benefits system, but as a child it was the bullying, we should aim for improvement in both for both age groups.”  “Bullying isn’t just in school but in the workplace too. I am late diagnosed and have been through many mental health services but they don’t work if they don’t take autism into account.”  “Key factors: enable people to be able to afford to live, and then get support from peers rather than have those around them make it worse ie bullying, and also reduce systemic sexism.”  “The bullying I experienced as a child and teenager had a massive effect on me. I am not sure I will ever fully recover.”  “bullying in school first as if children hadn’t bullied and shunned him and understood autism my son would not have felt suicidal in the first place. He was a happy boy before he went to school.”  “It was difficult to consider the impact without having a timeframe to consider it in. E.g. I think reducing bullying could have a huge impact, but probably very delayed.”  “Survival 1st, stop fear(benefits).Stop the bullying,ALSO in institutions and PRESS!Not 'on list'SEEKING diagnosis support desirable.INSTANT support is a must.Peer support skilled YES 121 trained,paid!”  “Improvements should be aimed at children then this should decrease trauma from things such as bullying and will give them the recognition, information and the tools needed to navigate life”  “Bullying in school prevents autistic ppl from evolving any social skills. It also drags into workplaces where we can't select who we have to work with. Also, make public spades more autism friendly”  “Bullying at school has deeply affected my confidence and mental health throughout my life. I am now 69. Later on I struggled at work and seriously considered suicide by tube train.”  “Bullying wrecks lives and autistic people are a target for this. Autistic people can only reach their full potential if bullying is stamped out atschool and work.”  “I had ptsd, anorexia and suicidal idiology triggered in me by bullying from my GP and an eating disorder psychologist. They directly caused me to feel suicidal. I’m not the only one.”  “my son as a 2:1 Law degree and is experiencing huge and extensive disability discrimination in the workplace which has lead to his second attempt at suicide”  “I want help from people who get it. Last year I got bullied by someone from Newport mental health team. It was meant to be a triage interview he made my situation worse due to lack of understanding.”  “I lived with suicide idealisation since my early teens, more support/awareness for undiagnosed but autistic children surviving family abuse/neglect is needed! I fell through EVERY safeguarding net!”  “My daughter (mid 30s) in regular crisis. We get no support from Ex Social Care, EPUT or S Ex Asperger’s Service & she was bullied by Housing Dept from 2019. H’less. Under confidentiality. Im helpless.”  “The survey addresses provision not looking at aggravating factors such as trauma, isolation, exclusion and misunderstanding. Building autistic agency and enabling people to follow their passion. Do it”  “Please consider poorer autistic people. All prisoners should be screened for autism and then heavily supported if they receive a diagnosis.”  I think that sex education should be considered more when looking after autistic people and preventing suicide because autistic people are at high risk for sexual exploitation.”  “My Child suffered bullying at school but with a lot of support from us they managed to get a degree, but they received no help from the NHS after 16 I feel they have been let down by the NHS and DWP”  "For the last section it should include if people are seeking a diagnosis of any other conditions. Reducing bullying in schools just won't happen. I've not experienced the NHS services so couldnt answer"  “education for autistic people around forming healthy relationships is paramount. My brother was abused for many years by a partner, police have to be trained in recognising signs when victims cannot” |
| 1. *Equality for diverse identities and needs*   Reflected in 6.3% of comments within the parent theme (55.7% of which were from formally diagnosed autistic participants, 34.3% from possibly autistic participants, and 10% from non-autistic participants).  [Back to the top](#Thematictable_staging) | “For suicide prevention we need radical social change with a redistribution of resources and disability, racial, queer, climate and all other forms of justice”  “Access to diagnosis more available and awareness to marginalised groups (POC, immigrants, high functioning [sic], women)”  “We need specialist trauma services for autistic women who have been raped or sexually assaulted. We are more likely to suffer this than our peers but there is no specialist support on the NHS”  “It would be nice if the suggestions were able to serve the needs of a broad range of autistic people including variation for other forms of marginalisation. This requires lived experience input.”  “The bredth of the spectrum make a one size fits all approach too hard. Co morbidities will have an affect on being able to use and access services. Autism isn't a standalone diagnosis.”  “Girls and women are diagnosed too late - poor MH. Longer wait times leads to deterioration in MH. Support & therapies NEED TO BE ADAPTED. Focus on strengths and interests rather than deficits helps.”  “The autistic spectrum is so wide, individuality makes creating a template for one size fits all very difficult.”  “Start early, train teaching staff, ALL nhs staff & police to recognise autism in children (& adults until it’s normal) INC WOMEN so everyone has access to help asap & suicide goes down in ASD patients”  “I think that, for young women and girls, awareness and intervention is especially important in the teenage years when masking/coping strategies can begin to break down.”  “I had recurrent crisis because I don't have support, even though I am diagnosed. This invisible disability is very stereotyped and people don't recognise it as such, especially in females.”  “Suicide prevention needs to be focused on undiagnosed females and diagnosing them because they're most likely to die.”  "CAMHS is not set up for autistic children- the system and staff fail completely. Teachers and SLT^[[7]](#footnote-7)^ do not understand autism in girls and cannot cope/comprehend with the fall out of undiagnosed autism."  “Autism is similar to any issue, it increases with poverty and intersectionality and trauma. All middle aged adults have not had access to diagnosis or can’t meet the costs of private assessment”  “Don't know all the systems but have important insights. Autistic people not all the same"  “Mainly NHS related choices because my experience with NHS MH [mental health] services has been terrible. Staff often don't know what it actually is and dismiss autistic girls as anxiety. NHS adult autism service good”  “As a woman who was diagnosed at 57, life could have been so different if bullying at schools was taken more seriously and presentation of autistic girls/women was understood. Knowledge is everything.”  “It was hard to rank & reduce, there are so many good ideas. G.P. awareness has to improve especially with old thinking amongst male G.P.s where women are concerned. Better neurodiversity training.”  “helping with everyday tasks is often overlooked but can help people a lot. Also support for people in the workplace and education is so important and diagnosing more women also important.”  "I think starting with supporting children would help them to need less support (emotionally) as adults. Many professionals don’t understand autism in females. Waiting times are too long for adults."  “I was actually having trouble with the phone version of this form, but I put better understanding of autism in women in girls due to personal experience. I wasn't diagnosed until I was 40.”  “Diagnosis in the UK is hard to get, especially for people who fit into certain groups. It seems the system is trying to prevent certain people from being diagnosed. Support should be for everyone.”  “The NHS is so stretched re: diagnosis, and many people women especially have no idea they're autistic so won't feel confident seeking a diagnosis, therefore resources/help should be widely available”  “Autistic women and girls have been overlooked for too long. This is beginning to improve but there is a way to go hence, I put it as the priority.”  “shorter waiting times and better testing for females i believe is a priority as many autistic people need support, which sadly no matter what we change, will never happen without a formal diagnosis.”  “Because of delays in diagnosis and lack of diagnoses in women and minority groups, anyone e who thinks they have autism and that it is impacting their ability to cope must be able to access resources”  “The medical profession, employers and education system need to update their understanding of autism and BELIEVE and not gawomen and girls when they are struggling with ND-rooted mental health issues”  "So many people go undiagnosed for too long - especially females and ethnic minorities. Going through life undiagnosed is mentally, emotionally, physically exhausting and traumatising = suicidal."  "There is no mention about recognising the risk to women and girls due to DV is higher for those autistic. Until services catch up with waiting lists for diagnosing services need to assume many are und"  “I think suicide in autistic women and girls is likely related to being bullied and ostracised while not knowing they are autistic. They begin to believe they deserve it and that they are the problem.”  “Girls and women are diagnosed too late - poor MH. Longer wait times leads to deterioration in MH. Support & therapies NEED TO BE ADAPTED. Focus on strengths and interests rather than deficits helps”  “Firmly believe early diagnosis would have helped mostly but then not enough support for female ASD and certainly not enough employers willing to accommodate”  “Autistic women in crisis often don’t know that they are autistic, get misdiagnosed and treated for the wrong thing, and the treatment doesn’t work or makes things worse.”  “most services don’t feel like they encapsulate all presentations of ASD, e.g. women, low needs. Also employment/finances is an issue for many autistics, support would help!”  “I really think there needs to be more research into diagnostic criteria for women and girls - I think going undiagnosed for 37 years of my life has made my mental health significantly worse.”  “It's very difficult to choose, I'm a late diagnosed woman who was told I can't be autistic because I am articulate. We need medical professionals to understand autism in girls and women.”  “Repeated experienced lack of knowledge and training with regards to ASD in NHS mental Health Services towards an adult woman.”  “Children and adults from poor backgrounds (maybe due to multi-generational autism in the family) are far less likely to be diagnosed and supported by the aporaphobic society we live in.”  "Early recognition is vital to mental health. Most NHS staff even in mental health settings don't recognise it, especially in women/girls/adults in general! Support sooner could stop suicide.”  "Gender-diverse autistic people need help now. We are dying.”  “Oliver McGowan training is too focused on learning disabilities and the poster person is a young white male reinforcing stereotypes. Extremely unhelpful for autistic women without a learning disability”  “Service should be for all ages - I find too much ageism as regards research, services ... everything is for young people. We missed out at that age.”  “I think there needs to be more support for autistic females during menopause & during periods as from personal experiences with myself & daughter, our mood dips & we become very anxious & depressed.”  “It is devastating to discover the lack of support for autistic girls. I am my daughters safety net, and there is no real support”  “MAKE THE AUTISM NHS ASSESMENT MORE SPECIFIC TO HIGH FUNCTIONING OLDER ADULTS WHO HAVE LIVED DECADES MASKING TRAITS AND BEHAVIOURS.”  “More help is desperately needed for late diagnosed autistic people. We can’t mask anymore!”  “I wish there was more support for autistic adults who can mask well but still need support.”  “Access to diagnosis more available and awareness to marginalised groups (POC, immigrants, high functioning, women) and awareness”  “92% of autistic women live with PMDD, PMDD causes severe depression in the luteal phase, in a lot of cases this involves suicidal thoughts. There is a lack of knowledge on PMDD at work and nhs”  “I hope that this will bring about changes particularly for undiagnosed females”  "The assessment for asd needs to gear up for girls. It's to much geared for boys. And the metal health's services are diabolical, I believe hubs in the community and schools should be the way forward"  “Please include in your list diagnosis support for non white people as they have more issues accessing diagnosis and help than women and women have a lot and you include them.”  “One issue is that older women do not have the opportunity to be assessed. Long waiting lists, the issues with assessing women, but key is we get turned away when we ask about assessment by GPs.”  “It took over 23 suicide attempts for them to rethink the diagnoses I was rejected from when 7 for being a girl. Since I have been able to access support allowing me to do a degree when I barly passed”  “I think it is brilliant you are doing this. Things need to change especially for females where so much is blamed on hormones and GPs aren’t aware of autism signs.”  “The NHS needs to do a lot better in recognising autism, especially in women and especially when we're distressed. The dismissal I faced should not happen, nor should routine misdiagnosis with BPD.”  "Personally 'groups' would not be of help. Biggest help would be educating others into acceptance. Teachers don't identify girls who study hard and keep to rules as struggling"  “Suicidal ideation in autistic people often comes from also being marginalised in another way, eg trans.”  “I am part of the lost generation of autistic women and I suspect that what we need may be different from younger women who have not had a lifetime of trauma and masking. Different solutions needed.”  “Overlap between autism and LGBTQ+ identity is important when working towards suicide prevention. The approach for reducing suicide among autistic people must be LGBTQ+ inclusive, and vice versa.”  “This work you are doing is so very important , one of the most dangerous psychological threats to autistic women is RSD ( rejection sensitive dysphoria ) co-existing with emotional instability”  “Thank you for undertaking this work. We need to do so much more for girls and adult women in recognising autism.”  “Pls focus on diagnosing women & girls appropriately, de-stigmatisation of ASD in all spheres.”  “Autistic women more likely to be in Domestic abusive relationships but more likely to recognise those as caring when they are controlling or coercive relationships and if profs not aware feed into it”  “This survey is very much needed. Too many autistic people struggle with their mental health and find it difficult to access support. There is huge gap in knowledge around autism especially in females.”  “Thank you for considering us. Society has rejected and laughed at us for so long and there's been little help, none for us women and girls who were ignored and doubted and still not been diagnosed”  “Isolation is the hardest thing. Also as high masking female, finding other girls like me is difficult. I have male friends both autistic and not and they’re all gross and annoying. Give me some girls!”  “Would have been interesting to see an option in healthcare on supporting autistics with catching freq. co-occurs e.g. ADHD, Gender dys.”  “I really think that there still isn’t enough focus on autism in women and girls, this needs to get better.  “There us an epidemic if police violence against us when we can't communicate under stress. Also perimenopause and pmdd need addressing as they cause high volume of suicides. Nobody warns us.”  “Within the spectrum of autism, there are categories of varying developmental problems and that could help identify those who have more negative dispositions and thinking processes”  “Great survey, resonated A LOT. Well done. People need so much more help than they are getting, females are particularly neglected. Huge progress to be made.”  “Please consider poorer autistic people. All prisoners should be screened for autism and then heavily supported if they receive a diagnosis.”  “I liked the fact that it included those without a formal diagnosis; females, in particular, may never get a diagnosis but struggle regardless.”  “Diagnosis for me was in a time autism in girls was a new concept. But my biggest struggle has always been the office politics neurotypical people can't seem to live without. This culture harms us.” |

**Full thematic table for views on provision of support to undiagnosed individuals**

Quick navigation:

**Theme 1:** [“Until access to diagnosis is universal, discrimination against self-diagnosis is unethical”](#Thematictable_undiagnosed_T1)

**Theme 2:** [“A diagnosis should be the gateway to services”](#Thematictable_undiagnosed_T2); *Subthemes* [“Nobody should be in a position where they can’t obtain a formal diagnosis”](#Thematictable_undiagnosed_T2s1); [“Services are already stretched”](#Thematictable_undiagnosed_T2s2); [Self-diagnosis “muddies the water”](#Thematictable_undiagnosed_T2s3)

| **Themes,** *subthemes* | All quotations |
| --- | --- |
| 1. **“Until access to diagnosis is universal, discrimination against self-diagnosis is unethical ”**   Contributed to by 36.6% of comments related to this topic (48.9% of these comments were from formally diagnosed autistic participants; 42% were from possibly autistic participants; and 8.9% were from non-autistic participants).  [Back to the top](#Thematictable_undiagnosed) | “I chose all, 'cos "thinks they might be autistic, self diagnosed, awaiting assessment, diagnosed" is a natural sequence followed by any adults who end up diagnosed”  "Until access to diagnosis is universal discrimination against self diagnosis is unethical"  “I think support should be available on symptoms basis, not just diagnosis basis. Do you have these symptoms, and would this service help? Then you should be entitled to help"  “I think having some things available to anyone who may need it is infinitely better than not having them at all. Accessibility can help everyone (like the drop curb effect)”  “I know undiagnosed autistics not on a waiting list. A lot of them are (eg) scared of doctors, or were denied being put on a list, or fear it would affect child custody so avoid diagnosis”  “Because of delays in diagnosis and lack of diagnoses in women and minority groups, anyone who thinks they have autism and that it is impacting their ability to cope must be able to access resources”  “I understand clinical diagnosis confirms presentation. ADULTS that receive late diagnosis late are born autistic and have faced many difficulties without the right support. Neuro diverse needs shared”  “We can know we are autistic, whether formally diagnosed or not. We can't make people wait 4 years for a certificate That wait can change functioning to actively suicidal simply due to lack of support”  “I believe too much emphasis is placed on a piece of paper to prove diagnosis. My brother told mental health services that he thought he may be autistic but this was simply ignored”  “In my experience, most people who suspect they are autistic probably are. It's not a diagnosis to choose or consider lightly. Therefore we should classify all the groups in need of additional support”  “I feel that so many autistic people are hesitant to identify themselves that I can't exclude those groups who aren't formally diagnosed. I see there is a weakness that this could be exploited”  “ANYONE suspecting autism needs aid”  “Make all support and services inclusive”  “While I think it's important that we know that "everyone is a little autistic" is rubbish I think support should be available/offered to anyone if it would potentially help them”  “I think people without a formal diagnosis should be able to access the support mentioned in the survey as even if it's found they aren't autistic, they can be signposted to appropriate services”  “Who would REALLY want to be autistic! The waiting list is so high now I think, if you think you are autistic you should get the same treatment as a diagnosed autistic person.”  “I have heard many accounts where suicide attempts are made by people (especially women) who are undiagnosed and did not know they were autistic. Everyone should have access to support reduce the risk.  “I'm lucky in that I have a diagnosis, but many are still waiting a diagnosis and some don't want to get a diagnosis because it may restrict any plans e.g., if they wish to emigrate to another country.”  "30+ years of under-testing means requiring diagnosis is stupid"  “The most important thing is having support available that's designed for autistics, as well as staff having knowledge about what autism looks like. Nobody should be dismissed.”  “Support for individuals with mild autism symptoms is crucial. They often share sensitivities but may not display them in daily behavior, making them more likely to be overlooked by society”  "Until there is faster diagnosis process in the NHS that is consistently offered to all then ask groups should be covered. I only have a formal diagnosis of ADHD despite asking for an ASD one."  “We need to help everyone who is diagnosed or who thinks they might be autistic until the Country is better positioned to support those in need.”  “Anyone who is in crisis should be given the opportunity to receive support. Anyone who is or believes themselves to be autistic should be given specialist support.”  “Waiting times for assessment and diagnosis are very long. By the time someone gets to crisis, or needs support they may not have been assessed. Not all autistic people desire a formal diagnosis”  “Due to long waiting times to obtain diagnosis and a lot of stigma surrounding autism + the lack of willingness to approve the autism diagnosis made by other professionals, it should be very inclusive”  “I think the ideas (e.g. training both NHS and education staff to spot undiagnosed autism) should be a available to everyone”  “Peer-diagnosis given the same legal status as medical”  “There are so many people who feel that they fit an autism diagnosis but are not able to get a diagnosis, for various reasons, until this is sorted out, everyone who thinks they might be autistic”  “Given the wait times for accessing diagnostic support, as well as barriers to getting referred in the first place, think it's important that people who are struggling can access help”  “Autism is similar to any issue, it increases with poverty and intersectionality and trauma. All middle aged adults have not had access to diagnosis or can’t meet the costs of private assessment”  “Many people don’t pursue diagnoses for fear of it impacting the healthcare they receive. It’s also either a long wait or very expensive. Until this is reformed, self-diagnoses must be validated.”  “undiagnosed autistics often have a harder time than diagnosed as there isnt an explanation for their differences. Only autistic people can know how to help others, people w/o it are often useless”  “These services should also be available for people who haven't even considered that they might be autistic. Anyone offering services should consider that anyone might be autistic.”  “Because autism is neurodiversity nobody should be excluded at the start. Make support and services inclusive to all then filter out those who don't need it.”  “I think that everyone who suspects that they are autistic should receive the same support as people with a formal diagnosis due to the difficulties and barriers in the system of obtaining diagnosis”  “‘Everyone thinks they’re autistic’ is damaging, but self-diagnosis is where it starts for most adults. Support while awaiting diagnosis will motivate govt to better fund diagnosis services.”  “Awareness and education of autistic traits (even without diagnosis) allows professionals to adapt their approach to "reach" potential people with autistic traits.”  “autism diagnosis is so expensive and out of reach that services absolutely need to be made available to all people”  “Even if somebody only suspects they're autistic, that suspicion must come from some set of symptoms, which may or may not be autism, which may be disabling and require accommodation in such areas.”  “Any people self identifying on the waiting list or waiting diagnosis turn out to be correct about having Autism. It is important to have the support when they need it”  “I think everyone who needs support should get it, formally diagnosed or not. I think the person who needs support should be very involved in deciding and specifying what type of support they get.”  “I think a lot of autistic people don't go diagnosed for a very long time. access to diagnosis is impossible. and accessing it is not very autistic friendly. especially when you have past trauma as well”  “Probably many undiagnosed autistic people out there. Need to find and help them so get waiting times down too.”  “Having support whoever you are and in any phase of the diagnostic journey is important.”  “Diagnosis is so hard to access currently so no matter who thinks they have autism, they need to have access to help. Education is also absolutely vital. I wouldnt have thought I was so broken”  “Self diagnosis and suspected autism are important to be included given the difficulties in getting a diagnosis”  “current- -diagnostiC-process-is-sO-slow-that-l-feel- anyone-brave-enough-to-consider-that-they-might-be-autistic-should-be-offered-support-as-this-may- help-confirm/refute- the-diagnosis”  “I don't think gatekeeping is necessary. And it only makes sense to have autistic people actually running things, since they know what matters”  “I am self diagnosed, awaiting formal diagnosis and have struggled to get support from my employer. I’ve also experienced pushback from colleagues who don’t understand my needs.”  “I’m currently self diagnosed awaiting assessment. I have 2 kids both autistic. I’ve always struggled finally makes sense to me and this is what I feel I need now”  “I think it is vital that a formal diagnosis is not used as a method of gatekeeping access to support, especially in the context of long waits for assessment and persisting stigma.”  “If the waiting time for diagnosis was shorter my answers would be different. Too many people assume they have autism.”  “Diagnosis in the UK is hard to get, especially for people who fit into certain groups. It seems the system is trying to prevent certain people from being diagnosed. Support should be for everyone.”  “Until access to assessment is improved, it's important to allow anyone who believes they may be autistic to access support”  “The most important thing is having support available that's designed for autistics, as well as staff having knowledge about what autism looks like. Nobody should be dismissed.”  “The NHS is so stretched re: diagnosis, and many people women especially have no idea they're autistic so won't feel confident seeking a diagnosis, therefore resources/help should be widely available”  “I have said to make available to all groups because diagnosis is so slow and difficult to obtain. If diagnosis was quicker and easier then anyone with, or waiting for a diagnosis would be workable.”  “Barriers to diagnosis means that given the high conversion rate of assessment to a diagnosis as an outcome that means its likely the person has needs that could be met by services adapted”  “I selected "all of these groups" because current systems for diagnosis are still broken. Until diagnosis is more accessible, support must be freely available”  “I think a lot of suicides is because of undiagnosed autism or misdiagnosed as other conditions I think its important that autistic people can obtain services that are helpful to them”  “No one should need to be diagnosed. Differences in individuals should be accepted and respected. No one should harm anyone else, no matter what condition they are diagnosed with”  “I was recently asked by the NHS to leave the waiting list for diagnosis as it had reached 18 years long. I'm very wary of anything that is diagnosis-dependent, knowing how unlikely it is you get one”  “Regardless of if they 'truly' are autistic if they're reaching out they need help”  “Red tape hurts autistics and makes it less likely for us to access any service. The services have to be as easily accessible as possible with as little red tape as possible”  “Formal autism diagnosis is slow and may lead to discrimination. This could be further complicated by whether private diagnosis was accepted by the NHS”  “Because of delays in diagnosis and lack of diagnoses in women and minority groups, anyone e who thinks they have autism and that it is impacting their ability to cope must be able to access resources”  “Self-diagnosis is unfortunately the next best thing when the NHS waiting lists are sometimes 4 years long, and private diagnosis costs are extortionately high. Hence my choice of “all groups”.”  “Everyone ought to be respected if they believe they have autism, as that could solve the problem fully, often diagnosis comes as the result of stress and there just isn’t time for a formal diagnosis.”  “Everyone who feels they may be autistic after significant engagement with information on the topic should be able to access unless and until diagnosis is widely and easily accessible.”  “Ed, empl, cj, have such life serious traumas they matter to all. Social care should be with wanting to confirm autism by d. NHS unless will put all in for autism ass unrefusably, can't be tied to it.”  "There is no mention about recognising the risk to women and girls due to DV is higher for those autistic. Until services catch up with waiting lists for diagnosing services need to assume many are und"  “Lack of diagnosis is often due to lack of access to diagnostic services. I was not diagnosed till my 30’s & both of my attempts were when I was an undiagnosed child/YA. Early support is vital.”  “I see self diagnosis as valid as waiting times are ridiculously long and also there are barriers to getting an assessment especially for older adults such as myself and my father.”  “The diagnosis process is so long and can be traumatic and a deterrent, therefore I have included those awaiting and those who think they are autistic.”  “It is quite difficult to get a diagnosis especially if you have not received one as a child or mask your symptoms. Those that think they are autistic, will have similar issues to those diagnosed.”  “Ideally, services to extend to people formally diagnosed and self diagnosing but do think diagnosis is important,sometimes people think they're autistic if have social anxiety, overlap but not same”  “I know undiagnosed autistics not on a waiting list. A lot of them are (eg) scared of doctors, or were denied being put on a list, or fear it would affect child custody so avoid diagnosis.”  “Waiting lists for assessment & diagnosis are so long that all support MUST include those waiting for assessment. It is 6+ years in my location.”  “Regardless of whether you're diagnosed or not, that help should be available to those who require it. Diagnosis is taking too long to hold back on those who need help.”  “The waiting lists are so long that undiagnosed people wouldn’t get help otherwise. I have no idea how to ‘do life’. I have no idea how to apply for benefits. The forms for autusm are too hard.”  “I wasnt diagnosed until I was 35, that doesn't mean I magically became autistic at 35, I just struggled through. I don't believe having/not having a diagnosis should be used to discriminate.  “I think support should be available on symptoms basis, not just diagnosis basis. Do you have these symptoms, and would this service help? Then you should be entitled to help  “I understand clinical diagnosis confirms presentation. ADULTS that receive late diagnosis late are born autistic and have faced many difficulties without the right support. Neuro diverse needs shared  “I think autism is much more prevalent than we realise, and self-assessment as autistic should be as accepted as self-assessment as gay.  “I would design access to support differently to how you have categorised it here (not necessarily diagnosis based).  “If you are autistic or think you are and looking for help then you may benefit equally. It could be very important to co-design and co- produce as an autistic persons involvement could be insightful.  “Autistic people are not always recognised as autistic or assessed and diagnosed. The question should always be asked be it education, mental health or criminal justice - Is this person autistic?  “Was tempted to say self-diagnosis minimum, but its important for support even for those who wish to find out more about themselves. Co-design/prod: I'd say neurodivergent rather than autistic specific  “I select for all, as the cost to get diagnosed is rather high. By only applying the help to the officially diagnosed and pending assessment it forces the hand of those who perhaps can't afford to..  “Given the long waiting times/ limits of the current diagnostic system, self-diagnosed autistic people should be fully supported in education, employment, healthcare etc  "As an AFAB (in their late 50s) who stands no chance of getting an autism diagnosis on the NHS, and who cannot afford to go privately, I feel really strongly about the validity of self diagnosis"  “With long waiting list and people being put off by yet another assessment, it is important to include self-identification and people who think they are autistic - usually this means someone is.”  “Not sure why you need to put barriers around any service, at least let people try something and decide for themselves it is not appropriate than foster feelings of treatment when you shut people out.”  “As autism is a lifelong condition/disability - not allowing support due to waiting for diagnosis invalidates the persons struggles”  “This is a dilemma for me because I was born in a age when autism was only considered if it was non verbal or severe, I was not formally diagnosed until age 40, self diagnosed at 35, diagnosis is key.”  “Autistic-led, person-centred, needs-led services and supports have been repeatedly demonstrated as key to better self-understanding, self-acceptance, improved autistic wellbeing and survival.” |
| 1. **“A diagnosis should be the gateway to services”**   Contributed to by the remaining 63.4% of comments related to this topic, which were divided between the following subthemes: | “I tend towards cat 3 (self-diag) is base level for most things. In my experience disclosure is very important to expect understanding, so some level of formal diagosis is vital”  “Whilst it feels very harsh to say services should priorotise those with a formal diagnosis. Self diagnosis is very complicated - a person may identify with a few traits but not be autistic”  “There are so many barriers to formal diagnosis, but I do think it is important for some accommodations to be moderated. In education + social care, hopefully ASD could be assessed to trigger this.”  “ Self diagnosis is valid, but for society to understand we need more diagnoses. Disabled people are so often vilified so I have little faith we can make a difference unless it’s strategic.”  “I think those already diagnosed and those waiting to be diagnosed should be prioritised. Police and courts should take it onboard more.”  “I think practical in person support for people who diagnosed or awaiting one should be top priority followed by organised and streamlined care then education and raising awareness of autism”  “People with diagnosed autism and waiting to be diagnosed have limited access to help and should be prioritised.”  “just have to be people who are formally diagnosed first, then those waiting for assessments. They would be the priority groups.” |
| 1. *“Nobody should be in a position where they can’t obtain a formal diagnosis”*   Contributed to by 26.6% of comments within the parent theme (68.4% of these comments were from formally diagnosed autistic participants; 18.4% were from possibly autistic participants; and 13.2% were from non-autistic participants).  [Back to the top](#Thematictable_undiagnosed) | “A lot of these questions relate to the slow NHS diagnose of autism. A diagnose should be the gateway to these services, if only older people could get one”  “Better access to diagnosis [would] remove need for self diagnosis”  “Self diagnosis is valid, but for society to understand we need more diagnoses. Disabled people are so often vilified so I have little faith we can make a difference unless it’s strategic”  “I think we should focus on radically improving diagnostic processes rather than redesigning entire institutions to accommodate undiagnosed people”  “Focus on reducing waiting times for diagnosis, rather than stretching support for all the above to everyone who thinks they MIGHT be autistic”  “Support needs to be across the spectrum, autistic people just like anybody else, would look at their own needs and that may not suit someone else on the spectrum, people who are not diagnosed should be”  “I wouldn't exclude any of the options if getting a formal asment was as easy as accessing testing for physical health conditions If it was as easy I would only include formally diagnosed or waiting to”  “assessments should be easily available to anyone who thinks they might be autistic, it should be as easy as getting a cancer screening”  “Better access to diagnosis - remove need for self diagnosis. All educational situations should be geared to support all equally, not identify special needs.”  “It should be quicker easier and less stigmatising to get a diagnosis so people don't have to self diagnose or guess”  “Diagnosis should be readily available and higher quality.”  “If people have or suspect they have autism, they might benefit from seeking this diagnosis and then unlocking further support related to the autistic experience”  “Would exclude people who think they're autistic if diagnosis was available v quickly.  "If diagnosis happens in a timely way only those with formal recognition should need services. Sorry but too many blaming autism for crimes and giving autistics a bad name."  “Unsure re ‘think they might be’..mostly that group need earlier assessment/diagnosis.”  “Criminal - I don’t want people to use as excuse. Education and nhs - cause I think they can get advise how to get diagnosed”  “I've put services should be limited to people with a formal diagnosis but this assumes better recognition and quicker assessment than is currently the case.”  “I think it’s a priority to improve accessibility and speed of formal diagnosis for autism so that everyone who needs it can access the necessary services”  “To clarify above I think self-diagnosed and suspected autistics should be offered diagnosis to receive support”  “I think the help should be mostly limited to people with a formal diagnosis although this relies on a diagnosis not taking years. This is why those waiting for diagnosis need some support as well.”  “I've selected only diagnosed or those waiting for diagnosis because I believe if someone thinks they're autistic or self identifies they should be able to access an assessment within a reasonable time”  “shorter waiting times and better testing for females i believe is a priority as many autistic people need support, which sadly no matter what we change, will never happen without a formal diagnosis.”  “If the access to waiting lists is improving in the near future it is enough to apply just to ppl on the waiting list or with a dx. If it is remaining difficult to be referred, extent to self dx”  “If autism assessments were easier to obtain you wouldn't need to include self diagnosis. I think screening tools could be used for access to autistic services if no formal diagnosis.”  “I've ranked access to this support assuming that diagnosis would be much improved by then.”  “I think exclusive services can be justifiable, but currently, having access to an autism assessment as an adult is a postcode lottery. Ideally, people should be formally diagnosed.”  “Difficult to narrow down or exclude whole groups from support as a Boolean option - ideally people who think they are Autistic should be able to get diagnosed, and not be waiting eg 3 years as I have”  “Included all the groups as I'd like those who self diagnose and think they might be autistic can tell health services/Autistism diagnosis services/GP and be properly supported towards confirmation”  “I think the whole area of self diagnosis or identifying as autistic is very tricky and only really comes about due to the crazy waiting times to be assessed.”  “people shouldn't need to self diagnose”  “I am not sure about people who self diagnose- those who think they may be neurodivergent should have access to information and assessment services quickly.” |
| 1. *“Services are already stretched”*   Contributed to by 14.3% of comments within the parent theme (72.7% of these comments were from formally diagnosed autistic participants; 4.5% were from possibly autistic participants; and 22.8% were from non-autistic participants).  [Back to the top](#Thematictable_undiagnosed) | “I think access should be restricted to provide focus and make sure it is available for those with a diagnosis, rather than trying to do it for too many and services getting overwhelmed”  “Conflicted about question referring to whether services should be available to those not formally dx'd. With limited resources I put formal DX only, but recognise there are barriers to formal DX”  “I believe that given limited resources (Either NHS, Education, Work, etc) people who are formally diagnosed or are awaiting a diagnosis should be prioritised. Otherwise systems could get overwhelmed"  “The ONLY reason I think self-diagnosed/ "might be" shouldn't get Employment/ CJS support is saturation would stop those who DO need it getting it”  “Formal diagnosis more likely to happen when someone is really struggling. I was diagnosed via nhs. There is however a lot of misinformation on social media about ND & services are already stretched”  “There are too many people self diagnosing themselves and this is taking away from the neednto support those people who have a medical diagnosis. The key is access to diagnosis."  “Ideally services should be available to all of the groups but where funding is limited I have prioritised those with a formal diagnosis or awaiting a formal diagnosis”  “All interventions will cost money so should be limited to those diagnosed autistic. If someone is diagnosed it means the condition has already effected their lives and is highly likely theyre autistic”  “In an ideal world everyone would get the support that they need. But if there are limits, those with a diagnosis or are on waiting lists/advised to get diagnosed should maybe be prioritised.”  “I would have put " all of these groups" were it not for knowing there are massive mass refunding issues and even those diagnosed can't get help because of that. Mixed feelings re " self diagnosis"  “I think access should be restricted to provide focus and make sure it is available for those with a diagnosis, rather than trying to do it for too many and services getting overwhelmed”  "The reason for limiting access: 1) Reality (funding); 2) The Elon Musks of the autism world using a perceived diagnosis to excuse bad behaviour"  “Thinking about prioritising the target group. Avoiding people claiming autism and claiming support “ people who feel entitled “ gaining support over those without a voice”  “Some services should be available to all formal or identifying with. However if all services were available to all then those who really needed it would struggle to access.”  “I think too many young people are self diagnosing based on unscientific videos and quizzes on social media and this is preventing access to those who are actually autistic.”  “I don’t like self diagnosis. It closes doors for people who are diagnosed. If they are worried about being autistic they should go down the formal route.”  “There are so little services that cost nothing and it's hard when you can't work but need help. I believe you should be formally diagnosed to get help or it gets overwhelmed”  “I think that, because resources are so limited, it is important that the people who need them receive them.”  “I think there is a balance to be struck between ‘a diagnosis for everyone’ and ‘getting the support and services I need.’ So… not sure a good idea to enable everyone to see themselves as autistic”  “Ensure the correct people get the specific help they need.”  “If you opened it to everyone who 'says' they thing they are or might be without engaging the gears of getting a diagnosis it opens it up to abuse and overburdening the system.” |
| 1. *Self-diagnosis “muddies the water”*   Contributed to by 59.1% of comments within the parent theme (70.2% of these comments were from formally diagnosed autistic participants; 12.3% were from possibly autistic participants; and 17.5% were from non-autistic participants).  [Back to the top](#Thematictable_undiagnosed) | “In general I believe services should be offered with or without a formal diagnosis, but this may be abused by a minority of neurotypicals claiming self-diagnosis, e.g. in the workplace”  “In some situations, people who are not struggling with autism might claim to be autistic to gain freedoms or advantages”  “I just feel there are people who will and have abused the system claiming ASD to behave inappropriately and get away with things. So I believe it's a delicate area”  “If you opened it to everyone who 'says' they thing they are or might be without engaging the gears of getting a diagnosis it opens it up to abuse and overburdening the system.”  “i think certain resources should be safeguarded so they can’t be exploited. Spotting signs of neurodiversity needs huge focus especially in education at all levels.”  “not including self diagnosis as open to fraud. Any autistic service needs to have massive input of autistic people"  “I selected only those with or awaiting a formal diagnosis because I fear any system that allows exceptions such as self-diagnosis would be open to abuse by non-autistic people.”  “Preference for formal diagnosis is entirely due to people using our condition as an excuse or 'clout'”  “Criminal justice - it would be important not to dilute sentences with false claims of autism. Formal diagnosis may have to be baked in to the process. Even tho I don’t agree with formal diagnosis!”  “l got my diagnosis at 64 years of age. I think it should be those diagnosed or awaiting a diagnosis. otherwise it could be manipulated”  “Thinking about prioritising the target group. Avoiding people claiming autism and claiming support “ people who feel entitled “ gaining support over those without a voice”  “self diagnos could lead to people benefitting the system for the people who genuinely need it”  “If certain services are available to those without formal diagnosis it is open to exploitation. However services in education should be open to all to detect autism in any degree”  “Some people could claim to be self diagnosed Autistic to evade blame.  "Difficult to decide as some people who self diagnose take advantage of systems they don't need, but not always the case"  “If you opened it to everyone who 'says' they thing they are or might be without engaging the gears of getting a diagnosis it opens it up to abuse and overburdening the system.”  “Orgs that are able to support a clinical referral to ASD assessment should accept all degrees of dx. Others eg CJS/Employment must be weary of self dx & excusing actions, invalidating the true ASD pts  "If diagnosis happens in a timely way only those with formal recognition should need services. Sorry but too many blaming autism for crimes and giving autistics a bad name”  “Self diagnosis is dangerous and should not be encouraged, it makes those of us with a diagnosis seen as skivers because we "made it up" Yes I have been told that, many times”  “People should not be allowed to self diagnose it muddies the water and makes it more difficult for those of us who are diagnosed”  “I think "self diagnosed" being able to access vital services for Autistic people could abused by predators. I'm a woman(51) who fought for 15 yrs to be accessed for Autism, I need safe spaces”  “The reason for limiting access: 1) Reality (funding); 2) The Elon Musks of the autism world using a perceived diagnosis to excuse bad behaviour"  “Criminal - I don’t want people to use as excuse. Education and nhs - cause I think they can get advise how to get diagnosed”  “I feel the term autistic is becoming meaningless, or at least no longer represents people with severe autism. The very people that need the most help are not having their needs considered”  “The key is sufficient funding into diagnosis. Is self diagnosis clogging the system? huge concerns that it is diluting my child’s reality. Causing a negative “everyone is Autistic now” culture”  “I’m sorry, but sharing these spaces with self-diagnosed people would ruin them. I have seldom related to them. It just makes me feel even more isolated. Plus resources are limited”  “I don’t like self diagnosis. It closes doors for people who are diagnosed. If they are worried about being autistic they should go down the formal route.”  “ Too much self-identifying. I don’t believe in it.”  “I dont believe self-diagnosis, it must be medical. Prevention of suicidal thoughts key, hence work+life opportunities critical: secondary is rapid diagnosis: last is crisis management.”  “Too many ppl self-diagnose w/out fully understanding how cripplingly painful it can be to be autistic. It isn't all hyperfocus, liking Harry Potter”  “Autism has become misunderstood and trendy, so many want to have it or think they have it when they do not. Resources for Autistics would be wasted on them”  “I think there is a danger with misuse if self diagnose but if in system for referrals after seeing GP then yes. Education before to include as that May flag up and support earlier.”  “I have concern for influencers making autism ‘trendy’, I find this invalidating. I used to support self diagnosis, but now want to be protected/separate so there is support for those in need.”  “I think too many young people are self diagnosing based on unscientific videos and quizzes on social media and this is preventing access to those who are actually autistic.”  “I understand why some people self diagnose, difficult to get a formal diagnosis through the NHS. Self diagnosis is a worrying trend. People strive to label themselves as autistic when they are not”  “There are simply too many people on waiting lists or self-diagnosing with no intention of seeking diagnosis who will take advantage of autism-services. they need to be explicitly for autistics”  “As someone who waited 3 years for my diagnosis through the NHS I’ve also noticed some people believe autism is fashionable. I do not like the idea of self diagnosis. There is a process !”  “I have often felt that my diagnoses are minimised by a few people I've met who are 'self diagnosed' but refuse to seek further help with this”  “I was diagnosed as a child and have a profound disability. My son also. Many people I know are not diagnosed (some went through the process and did not meet criteria) and are not impacted as much.”  “Self-diagnosis is not a diagnosis. The process is rigorous and long. Individuals who suspect they are autistic are not able to accurately and reliably reach a conclusion objectively”  “ I cannot understand why anyone in the UK would self diagnose as autistic. In the US it is expensive to get assessed, but not here.”  “The formal process for a diagnosis involves a professional who has conducted an assessment. People who self diagnose autism have not had access to this process and they might be ‘mis-diagnosing’”  “Compassion for those without diagnosis due to barriers however end result can be that they are not autistic therefore imperative that specific autism services are accessible to autistic individuals.”  “ My exclusions ie, diagnose as autistic/think they might be autistic etc is because they need a clinic physiologist/Dr to assessment to rule out any other mental health issue ie bipolar disorder or ?”  “With diagnosis or on waiting list assumes a definite or potential ASD. Other folks could have other conditions that would not necessarily be helped the same”  “If someone meets the criteria to be referred then they should be seen as on a waiting list if their GP takes them seriously or not. Self diagnosis could be dangerous”  “Self-diagnosis is a problem. Autistic people tend to be thorough seeking knowledge for self-dx & usually correct, non autistic people less able to differentiate, accuracy is critical to get better”  “I think where services are limited priority should go to those with a diagnosis or awaiting diagnosis. Someone without a dx might have different needs, ie. if their traits are due to trauma etc”  “Where i think self diagnosis is valid, it should be followed up by a referral for diagnosis. There are a lot of mental health issues which people self identify and it may not be an accurate diagnosis.”  “Being on the path to being diagnosed or being diagnosed autistic, is more realistic as it stops someone who may have another diagnosis being mistreated and the signs missed.”  “Ideally, services to extend to people formally diagnosed and self diagnosing but do think diagnosis is important,sometimes people think they're autistic if have social anxiety, overlap but not same”  “I think staff in services should be on the lookout for people possibly autistic. Services for autistic should be restricted: selfdiagnosis not accurate:not fair on those people to miss correct help”  “It's important that help be given to individuals that are formally diagnosed and awaiting assessment have priority. Self-diagnosing isn't always accurate and suspicions aren't always accurate either.”  “My take is that autism diagnosis should be carried out by a professional. Self diagnosis can be very counter productive in my view” |

**Full thematic table for analysis of views towards co-production**

Quick navigation:

**Theme 1:** [“Nothing about us without us”](#Thematictable_coproduction_T1); *Subthemes* [“Nobody knows better than we do about ourselves”](#Thematictable_coproduction_T1s1); [“We need to feel understood in crisis”](#Thematictable_coproduction_T1s2)

**Theme 2:** [“Caution” – the need for diverse representation](#Thematictable_coproduction_T2)

| **Themes,** *subthemes* | All quotations |
| --- | --- |
| 1. **“Nothing about us without us”**   Contributed to by 92.3% of comments related to this topic. Within this theme, 38.4% of comments emphasised the importance of co-design and co-production broadly (68.8% of these comments were from formally diagnosed autistic participants; 27% were from possibly autistic participants; and 4.2% were from non-autistic participants). The remaining comments (61.6%) reflected either one or both of the following subthemes as specific benefits of co-design and co-production:  [Back to the top](#Thematictable_coproduction) | “I am aware of the power of peer support. I believe autistic people should be involved at every stage including design and running services. This is genuine co-production not tokenistic involvement”  “Autistic people should be involved in the creation of improvements and creation of services for autistic people at every step”  “Being autistic I need to be involved in design of anything that is going to affect how I do something, it enables me to process the information better, allows me to do in a way I understand”  “The autistic experience varies between individuals, having an input from all reaches of the spectrum would help develop more holistic strategies”  “Contact the BPS about getting Autistics on the other side of the practioners table. Even the profession of psychology is discriminative. Bloody Galton & Darwin, how do you de-evolve what has evolved?”  “I think involving autistic people in every aspect of delivering training and in direct engagement is essential”  “Autistic people (diagnosed or self identified) should be involved at all levels.”  “Why does everything have to be done /for/ autistic people (badly)? We're plenty competent: we just don't get the funding.”  “Research should be undertaken into trusts with high rates of autistic suicide such as Sussex Partnership, scrutinising these to establish good and bad practice, in consult with autistic advocates.”  “I object to the reference to autistic people being involved in websites etc. They should be designed totally by autistic people. Not non autistic people needed.”  “All studies, creation of resources etc must involve autistic people.”  “Nothing about us without us.”  “Nothing about us without us”  “services designed for supporting people on the autistic spectrum. Must have the varied needs and personal experience of life at the centre of any design.”  “Autistic people HAVE to have input on all levels of supporting and researching autism ie training autistic individuals t or just getting the community involved"  “ND people NEED to be working with ND people. As an autistic woman, I have received better medical care from NHS women with autism.”  “I would love to see a network of autistic professionals leading on service design and delivery”  “Any autistic service needs to have massive input of autistic people"  “We need autistic diagnosticians only... I have seen far too many autistic individuals misdiagnosed or not at all because the clinician was NT and not equipped for the position.”  “Autistic-led, person-centred, needs-led services and supports have been repeatedly demonstrated as key to better self-understanding, self-acceptance, improved autistic wellbeing and survival.”  “Involving autistic people in what services and support are needed for them is very important as I think too many decisions are made without our input and this is not getting the services that we need”  “Autistic people should be involved in decisions.”  “Involving autistic people is critical”  “Resources accessible 24/7 & without needing to talk to someone are best in lead up to crisis, & must be autistic co-created, otherwise it's just adding to systematic oppression.”  “Having experienced mental health crisis myself many times, there is huge misunderstanding in mh teams and misdiagnosis of personality disorder. Coproduction is essential”  “Some choices which otherwise looked good were deselected as they didn't ensure services would be designed by and for autistic people. For instance many harmful 'classes' for autistic people exist.”  “Co production and training is vital but it has to be implemented which in my experience did not happen. Awareness - only useful with action. A&E - not a “safe space” for anyone in mental health crisis”  “It's absolutely imperative that autistic ppl design/run support groups/services for autistic ppl. Swap ""autism-friendly"" for genuinely empathetic & inclusive for all suggestions and EVERYONE benefits"  “ Too often we are told what we need instead of being asked”  “Services shouldn’t be co-produced by autistic people, it should be led SOLELY by autistic people. Also very explicit consent for all services and no mandatory reporting to police”  “No co production with non autistic people. Only autistic people involved in anything.”  “I have an IQ of 166 mensa and clinically quantified. I am an accomplished teacher, Level 2 Autistic. Furious with the language and model of me! Unbearable. Autistics to teach and support Autistics!”  “Nothing about us without us, autistic people should be involved in the creation of improvements and creation of service for autistic people at every step”  “I am aware of the power of peer support. I believe autistic people should be involved at every stage including design and running services. This is genuine co-production not tokenistic involvement.”  “Early identification and support is key. My experience is that a lot of the suicidal ideology stems from trouble communicating and being understood. Also, stop letting allistic people speak for us."  “I have prioritised co-production and improving mental health services. I feel these are first steps in improving outcomes related to self harm and suicide.”  “Autistic people MUST be forefront in designing and implementing the services that will be for other autistic people. Nothing about us without us!”  “Too many services and support options are based on the voice of parents/carers and families, not ASD voices."  “ It is most important that autistic people are in charge of autistic services and of explaining to/treating autistic service users”  “Specialised training / services should be designed and/or run by autistic people or at the VERY least have a team of autistic advisors. Listen to people when they tell you what they need :)"  “The autistic experience varies between individuals, having an input from all reaches of the spectrum would help develop more holistic strategies”  “Co production and design one of the most important points in the survey”  "Absolutely everything about autistic people should be designed and led by autistic people. I LOVE the idea of training and paying autistic people to support other autistic people."  Autism is such a wide spectrum, that it makes sense to involve autistic people in the design process, as no 2 have the same needs/wants”  “Give autistic people a voice to work with themxelvesx”  “I believe many autistic individuals are still overlooked or misdiagnosed. I also believe that support systems and programs designed and implemented by autistics may be more effective.”  “Nothing about us without us.”  “It’s hard to create beneficial services for autistic people without their input. I wouldn’t trust a service aimed at autistic people that wasn’t made by or involved autistic people in its process.”  “Imp people have input to anything involving them..or know others similar have been involved . Massively imp that 'pros' don't underestimate auties,** or think know one, know all'!!**or parents!”  “It's beyond critical to have autistic people involved in as many areas as possible.”  “The key phrase here for me is "Nothing about us without us" - inclusion in design is integral. We can't expect change without being the main drivers behind the change - so coproduction is vital”  “It would be nice to have a little say in a world that doesn't hear us  " Regarding the access to support - I would have marked differently, if the choices were more coherent. Regarding last question - the question itself means excluding / exclusion (if ASD is not involv"  “Autistic adults and n children should always be the first consulted when making a decision This is how I ranked my scores" |
| 1. *“Nobody knows better than we do about ourselves”*   Reflected in 52% of comments within the parent theme (57.8% of these comments were from formally diagnosed autistic participants; 23.4% were from possibly autistic participants; and 18.8% were from non-autistic participants).  [Back to the top](#Thematictable_coproduction) | “NT people have no idea what life is like for an autistic or ND person can they understand and develop things that can work for a ND person. systems need to be made by the users or it just won't work”  “Absolutely no point a NT person giving NT advice to a ND person… our brains don’t work the same way”  “You need autistic people involved else how do neurotpicals know they are doing it right? They may understand the theory, the practical is very different”  “Even those who are neurotypical and have a son who is autistic are not ever going to be entirely tuned into daily experience of being autistic and will always have an outsider’s perspective”  “Autistic individuals are extremely good with understanding complex needs and situations and should be involved at every level about deciding what happens next”  “I don't really feel you can understand Autism unless you experience a lifetime of discrimination, not heard, seen or noticed”  “It’s vital that autistic people design support services as neurotypical people do not understand us or our needs. They have to stop talking for us and over us. It’s killing us.”  “ To prevent autistic suicide you must start with MH service. They know nothing. They failed to even recognise I could be autistic. MH for autistics run by autistics - only way to go”  “ They are best placed to understand. The world is already too neurotypical”  “I want help from people who get it. Last year I got bullied by someone from Newport mental health team. It was meant to be a triage interview he made my situation worse due to lack of understanding.”  “ No matter how well informed and well intentioned you are, if you have not had the experiences yourself, you will not fully understand and may miss or assume something.”  “ It is essential that any support for Autistic people is designed and co-created by Autistic people. Otherwise it would be like me buying you a pair of shoes & asking someone else if they fit you.”  “Autistics understand autism better.”  “Autistic people should definitely be consulted when designing any sort of programme or initiative to support autistic people. Neurotypical notions of support do not help neurodivergent people. At all."  “autistic people should be used in helping advise experts in how to support us”  “ Too often support is designed without input from the people who will need it and therefor doesn’t meet our needs or is designed without a clear purpose.”  “Neurotypicals can do their best to understand the mind of an autistic person and what may help, but only autistic people themselves are able to fully understand what we NEED.”  “Non-autistic people will never understand despite training, we need autistic people trained to help so we really feel heard. Benefits system is designed to drive us to suicide.”  “ It takes one to know one - who better to deal with our complex needs than our own kind? We need to feel understood in crisis”  “Non-autistic people will never understand despite training, we need autistic people trained to help so we really feel heard”  “Re co-production of resources: there are any number of autistic mental health professionals who are able to create material. We know what’s needed & have the skills to produce it. Rarely opportunity.”  “HUGE stigma personally faced in all NHS services. Reduce harsh judgments from ignorance through education. Lived Experience is key for services development & provision.”  “Involving autistic people in the design and running of support services or products is crucial. Their unique insights ensure that the solutions are truly effective and tailored to their needs.”  “Coming from a ND family, we have always been told what autism looks like and what we need by non autistic people who often don’t know our bespoke needs. We and family know best about us”  “I think if it doesn't reflect the lived experience of autistic people then how can it be helpful?”  “As there are approximately 750,000 people who gave autism and no two people are the same.....it would be beneficial for their input as they would be able to say what treatment works best for them.”  “My son has a therapist who is also autistic and supports him in navigating the world. It is hugely important that services are designed and carried out with the help of those who understand autism”  “Ita so important because neurotypical people really don't understand alot of the challenges which autistic people experience.”  “undiagnosed autistics often have a harder time than diagnosed as there isnt an explanation for their differences. Only autistic people can know how to help others, people w/o it are often useless”  “It is vital autistic people both run and inform how these services operate. Nobody knows better than we do about ourselves.”  “We know what we need, and we know that other autistic people also know what they need.”  “Autistic people are experts by experience and are vital for understanding the processes and practices that are helpful or unhelpful”  “Autistic people themselves are the experts on how they experiance life with autism.Those who live with autism should be at the forefront of designing and running support services for autistic people”  “People who are Neurodiversent understand what their peers go through, it's as simple as," that, they can help each other, Gp are pretty much ignorant to Neurodiverscity, this must change as we are”  “In short, in order to create an efficient neurodivergent-friendly support system/network actual diagnosed neurodivergent individuals MUST be consulted for effective insider advice and knowledge.  Only people who are autistic fully understand the needs of autistic people. There would need to be a range of individuals diagnosed with autism involved.”  “It is very difficult for allistic people to understand how autistic people experience the world and the difficulties we face. Trained and supported autistic people are best placed to offer support.”  “I think that those who have experience are the best sources of information, however caution. Because there are many who only seek there own benefits, we must support our community needs wisely.”  “Autism is a lived experience. Those best qualified are those with first hand experience”  I think autistic peopke should. Be involvef because they understand what they need”  “Autistic people understand. A person trained in autism do not understand in my experience and alot of energy is used in explaining basics before talking about the problem.”  “It would be nice if the suggestions were able to serve the needs of a broad range of autistic people including variation for other forms of marginalisation. This requires lived experience input.”  "Nothing about is without us”  “Absolutely no point a NT person giving NT advice to a ND person...our brains don't think the same way"  "Autistic people dont think like neurotypical people"  “I don't think gatekeeping is necessary. And it only makes sense to have autistic people actually running things, since they know what matters”  “Autistic involvement is key ....insight! Support from another autistic person.... people feel they are not alone. Help with executive function....so overwhelming. Time off at crisis points."  “If you don’t have autistic people involved in the design I feel that many things would get infantilised or may not be clear enough/ information would not be accessible for autistic people”  “How can non autistic tell autistics what they need? You need several autistic contributors to cover a wide range of symptoms”  “Neurologically typical people can only do what they observe. Without the input of autistic lived experience services though well meant fail to really help. We need society to understand and accept us.”  “Having neuro divergent people involved in the design of services and support ensures that all needs are considered from a point of understanding and not from a neurotupical view of how we should think”  “Autistic people understand best how things are for them”  “Only autistic people can understand the lived experience of actually being autistic. We need to raise our voices and develop autistic led services. For too long we have allowed others to speak us.”  “Because we have direct and lived experience of the life long difficulties which impact all areas of our lives. We are a minority , and need to advocate for each other”  “Lived experience is key to developing autistic appropriate services”  “Simple minor changes and adjustments can make a huge difference but might not be apparent to neurotypical.”  “Lived experience is a big factor & surely a positive influence.”  “having support designed by autistics would be SO helpful as they know what we actually need help with, rather than what neurotypicals THINK we need help with”  “Lived experience input on this work is absolutely essential for working with places like schools/unis, hospitals, criminal justice teams etc”  “Autistic people have lived experience, a non Autistic person who may have acquired a PhD by reading the subject matter, has secondary experience unlike a person who has been diagnosed with autism.”  “People assume what autistic people need. Which is often wrong. It does not make sense to exclude them from deciding what is best for autistic people.”  “Even those who are neurotypical and have a son who is autistic are not ever going to be entirely tuned into daily experience of being autistic and will always have an outsider’s perspective.”  “Without consulting a vast array of autistics you can’t understand how to support us. We have common traits but we are all different.”  “ASD peoples brains don’t work like neuro typical brains so what neurotypical people think would work might not for them. It is also important to listen to autistic people as they are experts!”  “It could be very important to co-design and co- produce as an autistic persons involvement could be insightful.”  “Very important for lived experience to be included, the views and opinions of autistic people are vital to ensuring nothing is overlooked.” |
| 1. *“We need to feel understood in crisis”*   Reflected in 9.6% of comments within the parent theme (61.5% of these comments were from formally diagnosed autistic participants; 30.8% were from possibly autistic participants; and 7.7% were from non-autistic participants).  [Back to the top](#Thematictable_coproduction) | “I want help from people who get it. Last year I got bullied by someone from Newport mental health team. It was meant to be a triage interview he made my situation worse due to lack of understanding”  “Non-autistic people will never understand despite training, we need autistic people trained to help so we really feel heard”  “It takes one to know one - who better to deal with our complex needs than our own kind? We need to feel understood in crisis”  “We need the empathy and understanding we can only really get from other neurodivergent people”  “It is important to provide support from those that autistic people identify most with”  “The power and experience of autistic community is extremely impotant in combatting lonliness, so any autistic-led/peer-support group is essential.”  “People who are Neurodiversent understand what their peers go through, it's as simple as," that, they can help each other, Gp are pretty much ignorant to Neurodiverscity, this must change as we are”  “ Better services designed for Autists by Autists delivered in a timely manner. In a world not built for us compassion, grace and understanding give us hope for the future.”  “I am that seemingly rare animal : a social worker late diagnosed as autistic : what I have learnt is that autistic people often work really well with other autists so have autistics help other autists”  “I am aware of the power of peer support. I believe autistic people should be involved at every stage including design and running services. This is genuine co-production not tokenistic involvement.”  “Autistic involvement is key ....insight! Support from another autistic person.... people feel they are not alone. Help with executive function....so overwhelming. Time off at crisis points."  “ It is vital for insight to the barriers neurodivergent individuals face as no one neurodivergent is the same. it's like a colour wheel not linear. They all present differently.”  “Autistic people seem to connect and communicate with each other better than they do with 'normal' people. Something that I have observed over the year's.”  “I feel more invested and have trust when it is led by autistic people.” |
| 1. **“Caution” – the need for diverse representation**   Contributed to by 7.7% of comments related to this topic (30% of these comments were from formally diagnosed autistic participants; 40% were from possibly autistic participants; and 30% were from non-autistic participants).  [Back to the top](#Thematictable_coproduction) | “Having a great input from autistic people in the design and running of the support is inevitably going to result in it being geared towards more higher functioning individuals”  “I have limited my support for co-production and peer-mentoring because ‘activists’ who get involved in this may not always represent other autistic people well. Evidence-based action is key”  “I think that those who have experience are the best sources of information, however caution. Because there are many who only seek their own benefits, we must support our community needs wisely.”  “There is such a wide range or autism severity and experiences that in fact professionals who have seen a wider range of autistic expressions and challenges might be more helpful to design”  “ I think it's moderately important, but not very important, since autism is a spectrum, so although their input is important, some trained professionals could maybe even know more about it than us.”  I think a deep understanding is so important but I don’t hold the view that everyone needs to be autistic to be passionate about helping autistic people”  “Having a suicidal person with autism affects everyone in the family. I feel that everyone who is affected should have a say. My son is very ill and it's hard for him to have insight”  “I think things shouldn't always be led by those close to it. I also had to have IVF. You couldn't trust infertile people to run the ethics committees - they're just too close to it to step back”  “I think a deep understanding is so important but I don’t hold the view that everyone needs to be autistic to be passionate about helping autistic people.”  “Co-design/prod: I'd say neurodivergent rather than autistic specific”  “Support needs to be across the spectrum, autistic people just like anybody else, would look at their own needs and that may not suit someone else on the spectrum” |

**Supplementary item 6: Community involvement**

Throughout the lifespan of our two-phase study, we sought feedback from the wider autism community, including autistic people (with/without experience of suicidal thoughts/behaviour), individuals who currently supported, cared or advocated for an autistic person of any age or ability (with/without experience of suicidal thoughts behaviour), and those who had been bereaved by the suicide of an autistic person they supported. Below, we explain the points at which we sought community feedback and how we responded:

1. During the planning stage for Phase 1, authors RLM and DM sought views of autistic community members as regards the acceptability of the project’s endeavours by attending a regional support group to explain the planned approach. Approximately n=30 autistic people attended the meeting, and feedback appeared very positive (unfortunately, we have no record of this, as the discussion was all verbal and unrecorded). No specific feedback was provided.
2. In the design stage, our Phase 1 survey and all materials were reviewed by n=13 autistic people and n=9 people who support autistic people. We implemented changes such as adding additional reassurance to information sheets; making eligibility criteria clearer; and adding clearer wording to manage expectations around support that the research team could feasibly provide. At times, we received some contradictory suggestions or some we felt we could not feasibly address. For example, some autistic people liked that we signposted participants to services and resources (like the Samaritans) at several points throughout the survey. Others pointed out that these services are not dedicated to autistic people and might not be helpful for them. In this case, we decided it was better to offer some ideas for support even if they are not tailored for autistic people, as they may still be of some help to many, and included an explanation within the resource materials that explained this decision. Having reviewed the suggested changes, we responded to the panel by explaining, point by point, what we had done in relation to each suggestion and exactly why we could not implement some of the suggested changes.
3. Prior to recruitment for the Phase 1 survey, authors TAP and JC, along with colleagues at Autism Action, held one-to-one discussions with community members, including with those who have been bereaved by the suicide of an autistic loved one, talking about the wording and visuals that would sensitively and respectfully communicate our messages. Having revised our content accordingly, we sought feedback from a larger group, receiving responses from over 90 autistic people and supporters/allies collectively.
4. In the design stage for Phase 2, we retained as much content that had previously been reviewed and approved as possible. We then sought feedback on the Phase 2 survey from a panel of 66 autistic and 69 non-autistic individuals. These individuals affirmed that they understood the 63 ideas from Phase 1 (having read an explanation of how these were analysed and formulated). While they offered no suggestions towards clarifying those, they provided feedback regarding making the general structure of Phase 2 more accessible (for instance, providing a counter which indicated how many ideas participants had selected; providing clearer guidance on the four-step process before it began). As before, we sought to implement as many of the suggested changes as possible and responded to the panel explaining our responses to each comment.
5. We asked a community panel of six autistic people and three non-autistic supporters/allies of one or more autistic people to read this manuscript and provide feedback on its clarity and acceptability. We asked them if they felt that the paper would be helpful for autistic people and those who support them; whether the language used was respectful; whether they felt any of the findings or interpretations could be harmful for autistic people and their supporters/allies; and whether there were any additional comments they wished to make. We received numerous helpful comments. For instance, we had previously referred to ‘autistic people and those who know them best (i.e. their ‘supporters’)’, but changed this to ‘supporters and/or allies’ on the basis of feedback about the power imbalance and situation of autistic people as ‘needy’ and ‘vulnerable’. We similarly amended our wording in relation to a sentence where participant responses ‘corroborated expert consensus’, as community members rightly noted that this renders the expertise of the participants as inferior. Another change pertained to the fonts used to display autistic and non-autistic participants’ comments, where we had inadvertently previously used fonts which rendered the non-autistic contributions as bolder and perhaps suggestively more important. Not all suggestions were implemented: for instance, to the request to change the format to a non-academic style without ‘legalistic phrases’, we explained that we needed to write in the style of the journal but would be producing an accessible version of the paper forthwith. We explained all of our changes to the community members who had provided feedback.

**References**

1. Moseley R, Procyshyn T, Chikaura T, et al. Community priorities for preventing suicide in autistic people: a two-phase cross-sectional approach to guide policy and practice. In.

2. Adler RH. Trustworthiness in qualitative research. *J Hum Lact.* 2022;38(4):598-602.

3. Anney VN. Ensuring the quality of the findings of qualitative research: looking at Trustworthiness Criteria. *Journal of Emerging Trends in Educational Research and Policy Studies (JETERAPS).* 2014;5(2):272-281.

4. Dodgson JE. Reflexivity in qualitative research. *J Hum Lact.* 2019;35(2):220-222.

5. Society NA. *The Buckland Review of Autism Employment is published.* 2024.

6. Procyshyn TL, Moseley RL, Marsden SJ, et al. 'I did not think they could help me': UK-based autistic adults' reasons for not seeking public healthcare when experiencing suicidality.

7. Moseley R, Marsden S, Allison C, et al. “A combination of everything”: a mixed-methods approach to the factors which autistic people consider important in suicidality *Autism in Adulthood.*

8. O'Connor RC, Worthman CM, Abanga M, et al. Gone Too Soon: priorities for action to prevent premature mortality associated with mental illness and mental distress. *The Lancet Psychiatry.* 2023;10(6):452-464.

9. Pirkis J, Dandona R, Silverman M, Khan M, Hawton K. Preventing suicide: a public health approach to a global problem. *The Lancet Public Health.* 2024;9(10):e787-e795.

10. Hawton K, Pirkis J. Preventing suicide: a call to action. *The Lancet Public Health.* 2024;9(10):e825-e830.

11. Grant A, Williams G, Williams K, Woods R. Unmet need, epistemic injustice and early death: how social policy for Autistic adults in England and Wales fails to slay Beveridge’s Five Giants. *Social Policy Review.* 2023;35:239-257.

12. Marsden S, Eastham R, Kaley A. (Re)thinking about self-harm and autism: Findings from an online qualitative study on self-harm in autistic adults. *Autism.* 2024;0(0).

13. Gray E, Rumball F, Happé F, Quinton AM, Spain D. “Why does someone need to have a crisis before anyone will do anything?”: Perspectives of Autism Intensive Support Team staff. *Research in Autism.* 2025;123:202545.

14. McKinlay J, Wilson C, Hendry G, Ballantyne C. “It feels like sending your children into the lions’ den”–A qualitative investigation into parental attitudes towards ASD inclusion, and the impact of mainstream education on their child. *Res Dev Disabil.* 2022;120:104128.

15. Billington J, Loucas T, Knott F. “I liked school, but school didn’t like me”: Autistic young adults’ reflections on their mainstream primary school experiences. *Neurodiversity.* 2024;2:27546330241310174.

1. While the survey requested participants be from the UK, we did not disbar international participation; however, given the UK-centric nature of Phase 2 ideas and the questions analysed herein, we excluded these participants from present analyses. Notably, the diversity of the international sample also precluded their inclusion as a separate, meaningful group. The majority were from the USA (31%), followed by Canada (14.2%), Australia or New Zealand (8.8%), South Africa (3.5%), Asia (3.5%), South America (1.8%), or diverse European countries (37.2%). [↑](#footnote-ref-1)
2. Those who consented to participate but withdrew *before* the first round of prioritising ideas (n=353) included 301 autistic people (74 of whom were also supporters/allies) and 52 non-autistic supporters/allies (2 of whom had been bereaved by the suicide of an autistic person); these autistic participants had slightly lower levels of lifetime suicidality than the group retained (F [1, 3174] = 9.84, p = .002), but unfortunately no more is known about them, as they did not complete the demographic questions which were situated at the end of the survey. [↑](#footnote-ref-2)
3. NHS: National Health Service, the publicly-funded health system of the UK. [↑](#footnote-ref-3)
4. CAMHS: Child and Adolescent Mental Health Service, an NHS service branch for children and young people under age 18. [↑](#footnote-ref-4)
5. Integrated Care Boards, local NHS organisations responsible for planning, managing and funding regional NHS offerings. [↑](#footnote-ref-5)
6. PIP: Personal Independence Payment, one form of benefit that is administered by the Department of Work and Pensions (DWP). [↑](#footnote-ref-6)
7. SLT (or SALT): Speech and Language Therapists. [↑](#footnote-ref-7)
